# Stereoselective Synthesis of Spiro-Decalin Oxindole Derivatives via Sequential Organocatalytic Michael–Domino Michael/Aldol Reaction

**DOI:** 10.1021/acs.joc.2c01046

**Published:** 2022-07-25

**Authors:** Leonardo Straminelli, Francesco Vicentini, Antonio Di Sabato, Carmela Maria Montone, Chiara Cavaliere, Kari Rissanen, Francesca Leonelli, Fabrizio Vetica

**Affiliations:** † Department of Chemistry, Sapienza University of Rome, Piazzale Aldo Moro 5, 00185 Rome, Italy; ‡ Department of Chemistry, University of Jyväskylä, 40014 Jyväskylä, Finland

## Abstract

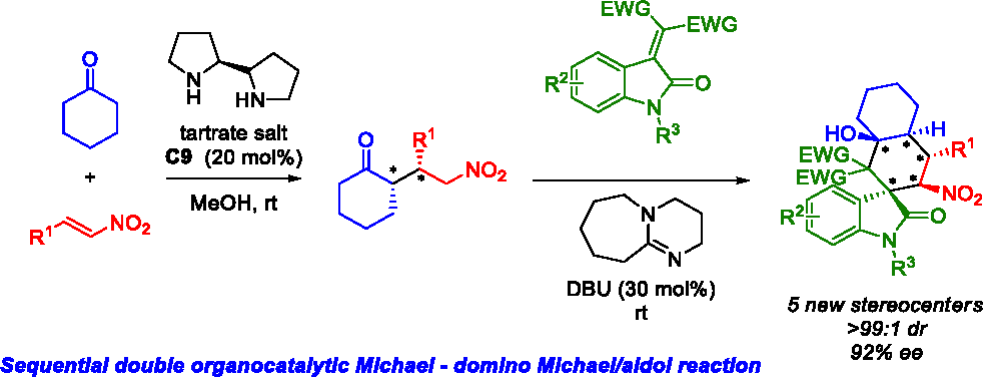

A highly stereoselective procedure for the synthesis of spiro-polycyclic oxindoles bearing five contiguous stereogenic centers including two tetrasubstituted carbons has been developed. Under sequential organocatalysis performed by a pyrrolidine-based organocatalyst and DBU, a highly atom-economical Michael–domino Michael/aldol reaction sequence was optimized, yielding variously functionalized spiro-decalin oxindoles with excellent stereoselectivity (>99:1 dr, up to 92% ee).

The asymmetric synthesis of complex heterocyclic scaffolds with multiple stereocenters in a stereoselective fashion represents one of the major challenges in modern organic chemistry. In the past two decades, many research groups have devoted their efforts in the exponential development of asymmetric organocatalysis, as an environmentally friendly and robust approach to achieve this aim.^1−8^ In fact, organocatalysts are usually stable in air and moisture and are characterized by a variety of possible activation modes of different functional groups.

In combination with the always-growing necessity for practically simple and eco-friendly synthetic procedures, asymmetric organocatalytic one-pot sequential reactions proved to be an effective and efficient approach toward the generation of structurally diverse molecular architectures from readily available starting materials.^9−11^

The oxindoles framework is a common scaffold in a plethora of natural and synthetic substances with various biological activities.^12−15^ In the realm of oxindole derivatives, of particular interest are spirocyclohexane oxindoles, which exhibit potential pharmaceutical applicability as, for instance, gelsamin (**A**) a glycine receptor agonist,^16^ anticancer compound **B** discovered by Hoffman-La Roche,^17^ or Satavaptan (**C**), a vasopressin-2-receptor agonist.^18^

Within this context, synthetic organic chemists have studied and optimized many protocols for the stereoselective organocatalytic synthesis of oxindoles derivatives.^19−24^ The most common route to achieve spirocyclic oxindoles relies on the use of 3-alkylidene-oxindoles, prepared straightforwardly by olefination of isatins, often commercially available 3-oxo-oxindoles.^23^

Because of our continuing interest in asymmetric organocatalytic methodologies toward valuable chiral building blocks and structurally diverse heterocycles,^25−29^ we envisioned the possibility to design a sequential organocatalytic protocol toward polyfunctionalized spiro-decalin oxindoles derivatives, starting from cyclohexanone (**1**), nitrostyrenes **2**, and 3-alkylideneoxindoles **3** (Figure 1, b). Indeed, the activation of cyclohexanone (**1**) via enamine formation to undergo Michael addition to an electron-poor olefin has become a key starting point for cascade reactions. Having selected two different Michael acceptors, such as **2** and **3**, we opted for a one-pot protocol, in order to control the regioselectivity of the reaction sequence by simple operational setup. Subsequently, the initial conjugate addition could be followed by a second Michael addition on the 3-alkylidene oxindoles **3**, which would then lead to a ring-closing aldol reaction and generate the spirodecalin moiety bearing 5 contiguous stereocenters, two of which are quaternary.

**Figure 1:**
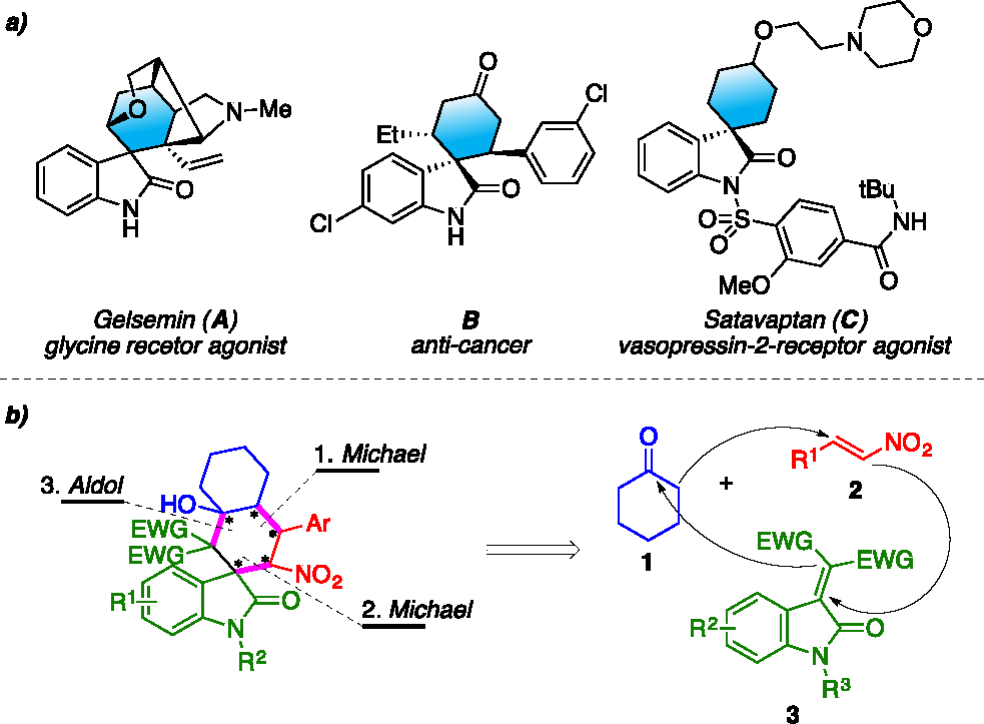
(a) Examples of bioactive spirocyclic oxindoles. (b) Our retrosynthetic analysis toward spiro-decalinoxindoles.

Our investigation started with the preparation of the Michael adduct **4a**, employing l-proline (**C1**, 50 mol %) as catalyst and equimolar amounts of substrates **1** and **2a** on a 3 mmol scale.^30^ Indeed, the organocatalyzed Michael reaction between these substrates has been largely investigated,^30−34^ and on the basis of the literature reports, we initially opted for an economical and operationally simple proline-catalyzed Michael reaction to get a considerable amount of product **4a**, to further investigate the envisioned reaction sequence. Intermediate **4a** was isolated and purified with 81% yield, >99:1 dr, and 52% ee.

Afterward, it was used in an initial test reaction with substrate **3a** in the presence of DBU (30 mol %) as basic organocatalyst in MeOH, to promote the domino Michael/aldol reaction (Table 1, entry 1).

**Table 1:**
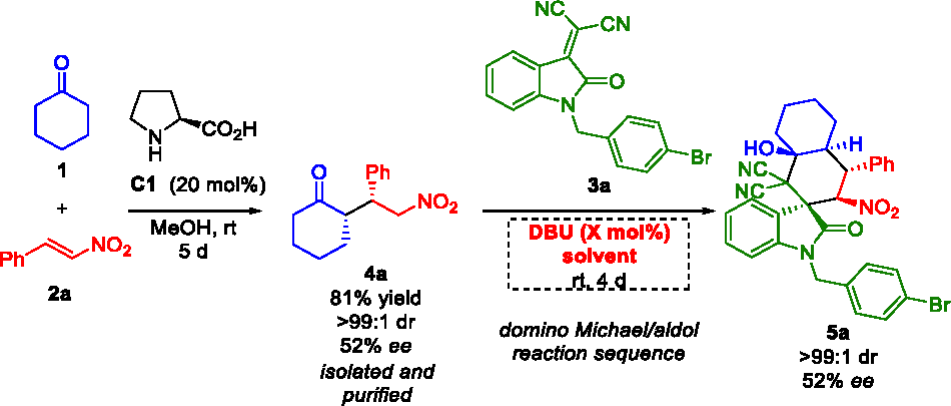
Step-by-Step Approach: Optimization of the Reaction Conditions for the Domino Michael/Aldol Reaction

entry^a^	solvent	DBU [mol %]	yield [%]^b^
1	MeOH	30	6 (25)
2	CH_2_Cl_2_	30	58 (74)
3	iPrOH	30	33 (57)
4	CHCl_3_	30	41 (64)
5	Hexane	30	10 (32)
6	Et_2_O	30	45 (67)
7	CAN	30	18 (42)
8	THF	30	25 (50)
9	CH_2_Cl_2_	20	51 (71)
10	CH_2_Cl_2_	40	16 (40)
11	CH_2_Cl_2_	50	16 (40)
12	CH_2_Cl_2_	100	4 (20)

a Unless otherwise stated, a solution of **1** (3 mmol, 1.0 equiv) and **2a** (3 mmol, 1.0 equiv) in MeOH (8 mL, 0.125 M) was stirred at rt in the presence of l-proline (50 mol %) for the indicated time. Product **4a** was isolated, and afterward, to a solution of **4a** (0.1 mmol), **3a** (0.1 mmol) and DBU (30 mol %) were added, and the reaction mixture was stirred for 4 d. In all experiments the dr values were determined via ^1^H NMR analysis, and the ee values via HPLC analysis on a chiral stationary phase.

b Isolated yields after flash column chromatography. Values in brackets correspond to the average yield per step.

Despite product **5a** being isolated with a poor yield of 6%, this initial outcome was extremely promising. Indeed, not only did it confirm the possibility to achieve the desired spiro-decalin oxindole scaffold, with the envisaged reaction sequence, but also it could be observed that the domino Michael/aldol reaction was extremely diastereoselective, leading to the final product with 5 contiguous stereocenters as a single diastereoisomer (>99:1 dr). With these outcomes in hand, we focused on the optimization of the reaction conditions by varying the solvent and the amount of base used (Table 1, entries 2–12). With our delight, we were able to increase the yield of the reaction to 58% (74% average yield considering the two reaction steps) while maintaining the same level of diastereocontrol, by using CH_2_Cl_2_ as solvent and 30 mol % of DBU (Table 1, entry 2).

It is worth mentioning that in all the tested cases we observed complete retention of the enantiomeric excesses (ee) from substrate **4a**, excluding any possible base-promoted racemization. With this in mind, we decided to focus our efforts on the optimization of the reaction conditions in the synthesis of intermediate **4a** to maximize the enantioselectivity, which would then be retained in the subsequent synthesis of the final product. As mentioned above, many organocatalytic protocols to achieve **4a** have been reported, furnishing excellent diastereo- and enantiocontrol. Nevertheless, in many cases the catalysts’ synthesis requires numerous reaction steps and tedious and harsh reaction conditions. Moreover, in order to reach high yields, 3 to 10 equiv of cyclohexanone are used.^31^ Consequently, envisioning an economical and environmentally friendlier synthetic procedure, we started a thorough screening of various readily available secondary amine-based organocatalysts, using methanol as solvent and keeping the substrate ratio to 1:1 equiv (Table 2, entries 1–11). The best results were obtained by using commercially available (2*S*,2′*S*)-2,2′-bipyrrolidine **C9**, used directly as tartrate salt (Table 2, entry 10). To our delight, the Michael adduct **4a** was isolated with 35% yield, >99:1 dr and 92% ee. Due to the chirality of l-tartaric acid, we decided to evaluate a possible involvement of tartrate counterions in the enantiocontrol. Thus, an additional trial was performed by initial treatment of the **C9** salt with base, to remove the tartrate. Probing the newly obtained secondary amine catalyst, we observed the formation of the desired product with a lower yield, but with a similar level of enantiomeric excess, confirming that only the bis-pyrrolidine scaffold is responsible for the enantiocontrol (92% ee, 16% yield, Table 2, entry 11). Additionally, these results corroborate the many literature findings in which an acidic additive increases the reactivity of cyclohexanone, favoring the enamine formation. Therefore, the simultaneous presence of the active chiral bipyrrolidine core and the acid additive in this commercially available catalyst represents a practical and economic advantage.

**Table 2:**
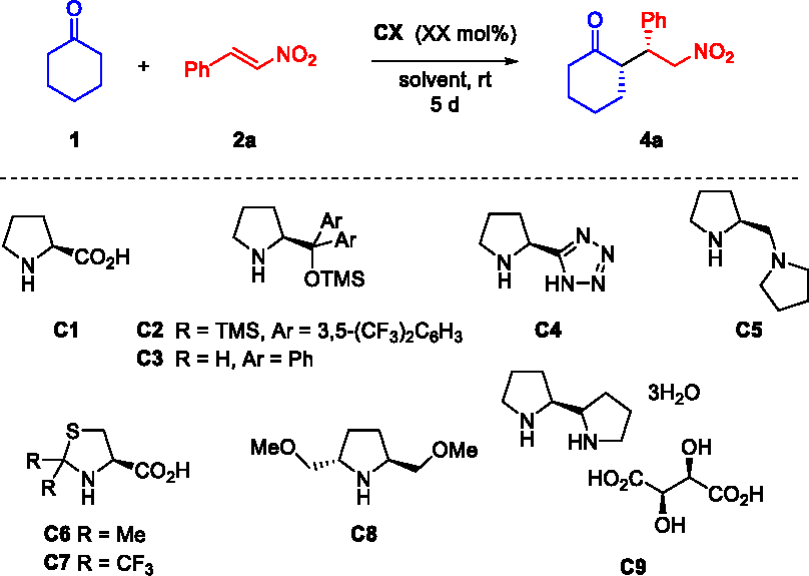
Catalyst Screening and Reaction Condition Optimization for the Michael Addition of Cyclohexanone to Nitrostyrene^a^

entry	cat.	solvent	cat. loading [mol %]	yield [%]^b^	dr^c^	ee [%]^d^
1	C1	MeOH	20	81	>99:1	52
2	C2	MeOH	20	n.r.	–	–
3	C3	MeOH	20	n.r.	–	–
4^e^	C3	MeOH	20	n.r.	–	–
5	C4	MeOH	20	99	>99:1	62
6	C5	MeOH	20	25	>99:1	82
7	C6	MeOH	20	n.r.	–	–
8	C7	MeOH	20	n.r.	–	–
9	C8	MeOH	20	n.r.	–	–
10	C9	MeOH	20	35	>99:1	92
11^f^	C9	MeOH	20	16	>99:1	92
12	C9	iPrOH	20	12	>99:1	90
13	C9	ACN	20	20	>99:1	60
14	C9	THF	20	8	>99:1	16
15	C9	CH_2_Cl_2_	20	16	>99:1	72
16	C9	CHCl_3_	20	4	n.d.	n.d.
17	C9	Et_2_O	20	8	>99:1	6
18	C9	hexane	20	6	n.d.	n.d.
19	C9	MeOH	30	34	>99:1	92
20	C9	MeOH	50	36	>99:1	92

a A solution of **1** (0.1 mmol, 1.0 equiv) and **2a** (0.1 mmol, 1.0 equiv) in MeOH (1 mL) was stirred at rt in the presence of the specified catalyst for the indicated time.

b Isolated yields after flash column chromatography.

c Determined via ^1^H NMR analysis.

d Determined via HPLC analysis on a chiral stationary phase.

e Reaction carried out in the presence of 20 mol % PhCO_2_H.

f Catalyst was pretreated with base and extracted to neutralize the ammonium salt and remove tartrate.

Further optimization involving catalyst **C9** has been carried out by analyzing the effect of both solvents and catalyst loading on the reaction outcomes (Table 2, entries 12–20). By changing from the initially used MeOH to iPrOH or CH_2_Cl_2_, we did not observe significant changes in the diastereoselectivity (dr >99:1); nevertheless, both the yield and enantioselectivity decreased considerably (Table 2, entries 12 and 15). Subsequently we tested the effects of less polar solvents, but in almost all cases both yields and enantiocontrol were significantly lowered. Further screening of the catalyst loading led to the identification of the best reaction conditions using 20 mol % of **C9** to provide 35% yield, >99:1 dr, and 92% ee (Table 2, entry 10).

Once we identified the optimal conditions for both the initial Michael reaction between **1** and **2** and the subsequent DBU-promoted domino Michael/aldol sequence, we envisioned the possibility of performing the three steps in a one-pot protocol.

Since the two sequential reactions are optimized in different solvents, we tested the possibility of replacing the solvent after the first Michael reaction, followed by the addition of **3** and DBU (30 mol %). Unfortunately, the desired final compound **5a** was isolated, while a complex mixture of products was detected. We performed some more trials varying the amount of DBU, to neutralize the 20 mol % tartaric acid present in the reaction mixture due to the catalyst; however, this strategy was also unsuccessful.

We believe that the presence of unreacted starting materials (**1** and **2**) could interfere with the domino Michael/aldol sequence and lead to the formation of other side-products. Consequently, to better control the reaction pathway toward the desired spiro-decalin oxindoles **5**, we opted for the initial isolation and purification of the Michael adduct **4**, prior to starting the following DBU-promoted diastereoselective domino Michael/aldol reaction.

Thus, we initially scaled-up the enantioselective **C9**-catalyzed Michael reaction using equimolar amounts of **1** and **2** (3.0 mmol). Comparable reaction results were obtained by simply prolonging the reaction time, isolating the intermediate with 38% yield, >99:1 dr, and 92% ee, confirming the applicability of this protocol also to a larger scale (Scheme 1). Moreover, no side-product formation was detected, and the unreacted starting material could be completely recovered, confirming a high-atom economy and complete conversion in favor of the desired product **4a**.

**Scheme 1:**
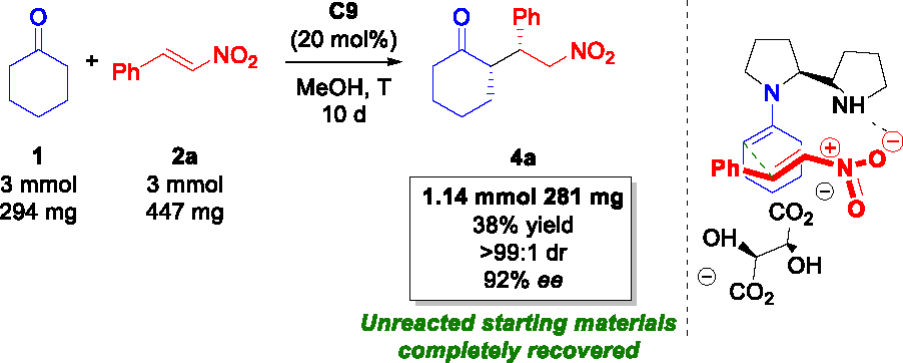
Scale-up Synthesis of the Michael Adduct **4**^,^^,^^,^ and Postulated Transition State See the Experimental Section for reaction conditions. Isolated yields after flash column chromatography. dr values determined via ^1^H NMR analysis. ee values determined via HPLC analysis on a chiral stationary phase.

On the basis of literature reports for analogous organocatalysts used in this Michael reaction^35^ and on the obtained absolute configuration, we proposed a possible transition state (Scheme 1). The tartaric acid assists the enamine formation, while the pyrrolidine free NH could coordinate the nitro group of **2a**, directing the nucleophilic attack on the β-position *Re* face, leading to the *syn* diastereoisomer.

Subsequently, compound **4a** was used as substrate for the following domino Michael/aldol sequence with alkylidene oxindole **3a** under the optimized reaction conditions. The final product **5a** was obtained with a good yield of 58%, which corresponded to an average yield per step of 76% (considering 2 steps), complete diastereoselectivity of >99:1 in the insertion of the 3 new stereocenters, and complete retention of the enantioselectivity (92% ee, Scheme 2). Additionally, comparable excellent results could be obtained by scaling up the synthetic procedure to 1.0 mmol scale.

**Scheme 2:**
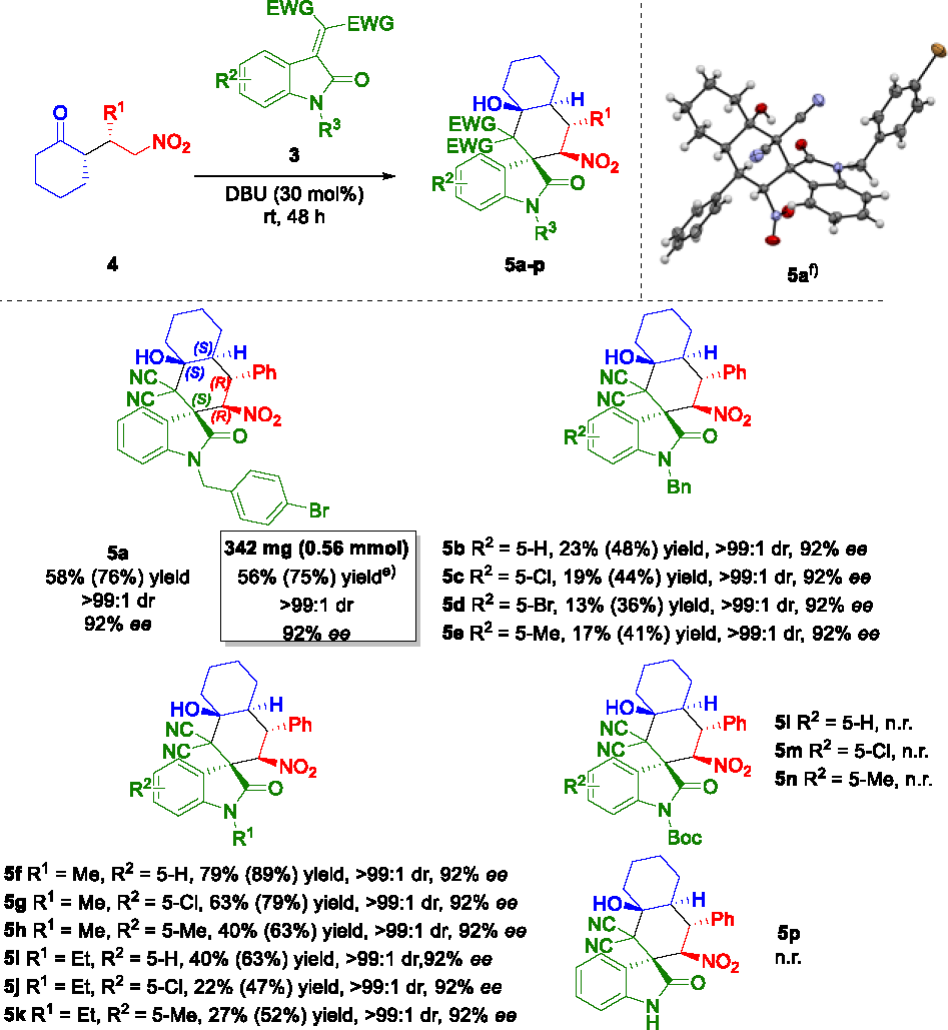
Substrate Scope of the Sequential Michael–Domino Michael/Aldol Reaction^,^^,^^,^ See the Experimental Section for reaction conditions. Isolated yields after flash column chromatography. Values in brackets correspond to the average yield per step. dr values determined via ^1^H NMR analysis. ee values determined via HPLC analysis on a chiral stationary phase. Reaction performed on a 1.0 mmol scale. X-ray crystal structure of compound **5a**(36)

Afterward, in order to demonstrate the general applicability of the developed procedure, variously substituted alkylideneoxindoles were probed under optimized reaction conditions (Scheme 2). By replacing the *N*-substituent with Bn and adding aromatic substituents on **3**, products **5b**–**e** were isolated with lower yields, while maintaining a high level of stereocontrol (>99:1 dr, 92% ee). Specifically, the yield was lowered in the presence of sterically hindering groups in position 5, such as Br or Me. On the contrary, improved yields were observed by changing the *N*-protection with a methyl group for products **5f**–**h**, with outcomes up to 79% yield (89% average yield per step) and the same high stereoselectivities.

Subsequently, the possibility of using alkylidene oxindoles bearing a free NH on the lactam moiety were unsuccessful. This outcome could be explained by the acidity of this site, which would interfere with the action of the DBU in promoting the domino reaction sequence. Nevertheless, even performing the reaction with an excess of DBU did not lead to the detection of the final product. Thus, we opted for a further variation of the *N*-protection with a Boc group. Unfortunately the presence of this protecting group did not lead to the final product. A complex mixture of products of difficult identification was isolated instead, presumably due to an in situ deprotection of the Boc group or by decomposition of the alkylidene/intermediated by lactam ring opening.

Finally, an additional functional group variation was considered; particularly, we introduced an N–Et group instead. With our delight, the reaction also worked straightforwardly with this alkyl substitution, with similar excellent results to the N–Me ones in terms of stereoselectivity, despite slightly lower yields.

The absolute configuration was unambiguously determined by X-ray crystal structure analysis of compound **5a**, and by analogy the configuration of all other products was assigned accordingly (Scheme 2).^36^

In conclusion, we developed an efficient and atom-economical methodology for the synthesis of highly functionalized spiro-decalin oxindole derivatives employing a stereoselective organocatalytic Michael–domino Michael/aldol reaction sequence. We observed good yields for a domino transformation (13–79%), which correspond to an average yield per step ranging from 36 to 89%. During this process, 5 new stereocenters are generated with virtually complete diastereoselectivities (>99:1 dr) and excellent enantioselectivities (92% ee), under mild and practically simple reaction conditions. Due to the importance of spiro-oxindoles in medicinal chemistry, the presence of easily modifiable groups and the high enantioselectivity could lead the way for late-stage functionalization in the search of potentially bioactive compounds.

## Experimental Section

### General Information

Unless otherwise noted, all commercially available compounds were used without further purification. For preparative column chromatography SIL G-25 UV252 from Macherey-Nagel, particle size 0.040–0.063 nm (230–240 mesh. flash) was used. Visualization of the developed TLC plates was performed with UV irradiation (254 nm). Optical rotations were measured on a DIP-370 Jasco polarimeter. GC-MS analyses were performed using an Agilent HP 5892 series II GC (phenyl silicone column 30 m × 0.25 mm × 25 mm) coupled with a mass spectrometer HP 5972 MSD operating at 70 eV. Elution program: initial *T* = 100 °C for 4 min, increasing the *T* at 10 °C/min rate, up to 250 °C. HRMS were by directly infusing solutions with a concentration of 1 ng μL^–1^ prepared in methanol at a flow rate of 10 μL min^–1^. MS was performed using hybrid quadrupole-Orbitrap mass spectrometer Q Exactive (Thermo Fisher Scientific) with a heated ESI source, operating in both positive and negative ion modes. For both ion modes, the full-scan MS acquisition range was 130–1000 *m*/*z*, the resolution was set to 35 000 (full width at half-maximum, fwhm, @200 *m*/*z*). The mass spectrometer was externally calibrated every 48 h, within a mass accuracy of 1 ppm, using the commercial Pierce positive and negative calibration solutions (Thermo Fisher Scientific). Raw data files were acquired by Xcalibur software (version 3.1, Thermo Fisher Scientific). ^1^H and ^13^C{1H} spectra were recorded at ambient temperature on Bruker Avance 400 or 300 spectrometers. Analytical HPLC was performed on a Shimadzu LC-20AD HPLC instrument equipped with a PDA detector (Shimadzu SPD-M20A) and a refractive index detector (Shimadzu RID-20A), using chiral stationary phases (Chiralpak IA). For the preparation of racemic compounds, a mixture of l-proline and d-proline was used.

### General Procedure for the Synthesis of *N*-Benzylisatins

To a solution of isatin (1.0 mmol, 1.0 equiv) and Na_2_CO_3_ (3.0 mmol, 3.0 equiv) in 8 mL of acetonitrile (0.125 M), benzyl bromide was added (1.0 mmol, 1.0 equiv). The mixture was stirred under reflux for 24 h using an oil bath and afterward allowed to cool to room temperature. Then, the solvent was evaporated under reduced pressure, and the crude was dissolved in AcOEt and extracted with a basic aqueous solution of Na_2_CO_3_. The combined organic phases were dried (MgSO_4_ an.), filtered, and then concentrated under reduced pressure. The product was isolated after flash chromatography on silica gel. Unless otherwise stated, the compounds were prepared following this procedure and all the analytical data were in accordance with the literature reports.^37,38^

#### 1-(4-Bromobenzyl)indoline-2,3-dione

Prepared from the general procedure using 4-bromobenzyl bromide as substrate. The analytical data are in accordance with the literature data.^39,40^ The product was isolated after flash chromatography on silica gel (hexane/EtOAc 6:4) as an orange solid (190 mg, 60%). ^1^H NMR (400 MHz, CDCl_3_) δ = 7.60 (d, *J* = 7.5 Hz, 1H, CH_arom_), 7.52–7.41 (m, 3H, CH_arom_), 7.24–7.15 (m, 2H, CH_arom_), 7.10 (t, *J* = 7.7 Hz, 1H, CH_arom_), 6.74 (d, *J* = 7.9 Hz, 1H, CH_arom_), 4.87 (s, 2H, CH_2_) ppm. ^13^C{1H}NMR (101 MHz, CDCl_3_) δ = 183.1, 158.3, 150.5, 138.5, 133.7, 132.3, 129.2, 125.7, 124.2, 122.3, 117.8, 110.9, 43.5 ppm. GC-MS (EI^+^, 70 eV) *m*/*z* (%) 316.9 (47), 314.9 (47), 259.9 (12), 257.9 (12), 180.0 (19), 170.9 (19), 168.9 (19), 145.9 (100), 90.0 (34), 89.0 (14).

### General Procedure for the Synthesis of *N*-Methylisatins

Prepared following a reported procedure starting from commercially available isatins.^41^ Unless otherwise stated, the compounds were prepared following this procedure and all the analytical data were in accordance with the literature reports.^37,42−44^

### General Procedure for the Synthesis of *N*-Ethylisatins

Prepared following a reported procedure starting from commercially available isatins.^45^ Unless otherwise stated, the compounds were prepared following this procedure and all the analytical data were in accordance with the literature reports. To a solution of isatin (1.0 mmol, 1.0 equiv) and K_2_CO_3_ (1.5 mmol, 1.5 equiv) in 3 mL of DMF (1 M), ethyl bromide was added (1.1 mmol, 1.1 equiv). The mixture was stirred for 12 h at room temperature. After TLC monitoring, cold water was added (20 mL) and a red suspension was formed. Then, after filtration and washing with water, a red solid is obtained. Unless otherwise stated, the compounds were prepared following this procedure and all the analytical data were in accordance with the literature reports.

### General Procedure for the Synthesis of 2-(2-Oxoindolin-3-ylidene)malononitriles (**3**)

To a solution of *N*-protected isatin (1.0 mmol, 1.0 equiv) in 10 mL EtOH (0.1 M), malononitrile (1.0 mmol, 1.0 equiv) was added and the mixture was stirred at reflux for 3 h using an oil bath. Afterward, the formed suspension was filtered to isolate a deep red/purple solid. The solid product was washed with cold ethanol and then dried. No further purification was needed. Unless otherwise stated, the compounds were prepared following this procedure and all the analytical data were in accordance with the literature reports.^46−50^

#### 2-(1-(4-Bromobenzyl)-2-oxoindolin-3-ylidene)malononitrile (**3a**)

The product has never been reported before so a complete characterization is reported. Prepared on a 3.0 mmol scale following the general procedure. Purple solid (901 mg, 95%). mp 212–214 °C. Molecular formula: C_18_H_10_BrN_3_O. Molecular mass: 364.20 g mol^–1^. ^1^H NMR (600 MHz, CDCl_3_) δ = 8.14 (d, *J* = 7.7 Hz, 1H, CH_arom_), 7.54–7.41 (m, 3H, CH_arom_), 7.24–7.09 (m, 3H, CH_arom_), 6.75 (d, *J* = 7.9 Hz, 1H, CH_arom_), 4.87 (s, 2H, CH_2_) ppm. ^13^C{1H} NMR (151 MHz, CDCl_3_) δ = 164.7, 162.7, 149.1, 146.0, 137.8, 133.3, 132.4, 129.4, 127.1, 124.3, 122.6, 118.5, 112.3, 110.7, 110.5, 43.8 ppm. GC-MS (EI^+^, 70 eV) *m*/*z* (%) 170.9 (96), 168.9 (100), 90.0 (27), 89.0 (20). HR-MS (ESI^+^) *m*/*z* calcd. for [M]^+^ = [C_18_H_10_BrN_3_O]^+^: 363.0007, found 364.0094.

### Asymmetric Scaled-up Organocatalytic Synthesis of Michael Intermediate **4a**

To a solution of cyclohexanone 1 (3.0 mmol, 1.0 equiv) in MeOH (8.0 mL, 0.125 M) were added the nitrostyrene **2a** (3.0 mmol, 1.0 equiv) and the catalyst **C9** (0.6 mmol, 20 mol %), and the mixture was stirred at room temperature for 10 days. After the elapsed time, the reaction mixture was concentrated under reduced pressure. The crude product was purified by flash chromatography to give the desired (*S*)-2-((*R*)-2-nitro-1-phenylethyl)cyclohexan-1-one **4a** as a colorless solid. 281 mg of the final product were isolated (38% yield, 92% ee, >99:1). The analytical data are in accordance with literature reports.^30^ HPLC: IA, hexane/iPrOH 95:5, 1.0 mL/min, dr >99:1, *τ*_minor_ = 11.2 min *τ*_major_ = 13.8 min.

### General Procedure for the Organocatalytic Stereoselective Domino Michael/Aldol Reaction (**5**)

To a solution of **4** (0.1 mmol, 1.0 equiv) in CH_2_Cl_2_ (1.0 mL, 0.1 M) were added the alkylidene **3** (0.1 mmol, 1.0 equiv) and DBU (4.5 μL, 0.03 mmol, 30 mol %), and the mixture was stirred for 4 d. After the elapsed time, the solvent was evaporated under reduced pressure. The crude product was purified by flash chromatography to give the desired product **5**.

#### (3*S*,3′*R*,4′*R*,4a′*S*,8a′*S*)-1-(4-Bromobenzyl)-8a′-hydroxy-3′-nitro-2-oxo-4′-phenyl-3′,4′,4a′,5′,6′,7′,8′,8a′-octahydro-1′*H*-spiro[indoline-3,2′-naphthalene]-1′,1′-dicarbonitrile (**5a**)

The product **5a** was isolated after flash chromatography on silica gel (H/EtOAc 7:3) as a colorless solid (53 mg, 58% yield, >99:1 dr, 92% ee). Molecular formula: C_32_H_27_BrN_4_O_4_. Molecular mass: 611.50 g mol^–1^. HPLC: IA, hexane/iPrOH 95:5, 1.0 mL/min, dr >99:1, τ_minor_ = 11.5 min τ_major_ = 15.3 min; OR: [α]^20^_D_ = +7.19 (c 1.90, CH_2_Cl_2_) mp 196–198 °C ^1^H NMR (400 MHz, CDCl_3_) δ = 7.91 (ddd, *J* = 7.8, 1.2, 0.5 Hz, 1H, CH_arom_), 7.55–7.21 (m, 9H, CH_arom_), 7.11 (dt, *J* = 7.0, 1.8 Hz, 1H, CH_arom_), 6.93 (d, *J* = 3.0 Hz, 1H, CH_arom_), 6.79 (ddd, *J* = 8.0, 1.1, 0.5 Hz, 1H, CH_arom_), 5.52 (d, *J* = 11.9 Hz, 1H, CHNO_2_), 5.06 (d, *J* = 15.8 Hz, 1H, CH_2_Ph), 4.96 (d, *J* = 15.8 Hz, 1H, CH_2_Ph), 4.18 (t, *J* = 12.0 Hz, 1H, CHPh), 2.41 (td, *J* = 11.9, 3.1 Hz, 1H, CHCHPh), 2.29–2.21 (m, 1H, CHH), 2.00 (tt, *J* = 12.1, 3.5 Hz, 1H, CHH), 1.95–1.83 (m, 1H, CHH), 1.78–1.52 (m, 4H, CHH, CH_2_, OH), 1.33–1.15 (m, 2H, CH_2_) ppm. ^13^C{1H} NMR (101 MHz, CDCl_3_) δ = 173.5, 142.2, 134.9, 132.7, 132.4, 131.4, 129.6, 129.4, 129.3, 128.9, 125.6, 125.1, 124.8, 122.5, 121.8, 113.0, 111.1, 110.5, 92.5, 78.6, 54.8, 50.5, 46.0, 45.1, 43.2, 37.3, 25.4, 25.4, 21.0 ppm. HR-MS (ESI^+^) *m*/*z* calcd. for [M + H]^+^ = [C_32_H_26_N_4_O_4_Br]^+^: 611.1288, found 611.1284.

#### (3*S*,3′*R*,4′*R*,4a′*S*,8a′*S*)-1-Benzyl-8a′-hydroxy-3′-nitro-2-oxo-4′-phenyl-3′,4′,4a′,5′,6′,7′,8′,8a′-octahydro-1′*H*-spiro[indoline-3,2′-naphthalene]-1′,1′-dicarbonitrile (**5b**)

The product **5b** was isolated after flash chromatography on silica gel (H/EtOAc 6:4) as a colorless solid (12 mg, 23% yield, >99:1 dr, 92% ee). Molecular formula: C_32_H_28_N_4_O_4_. Molecular mass: 532.60 g mol^–1^. HPLC: IA, hexane/iPrOH 95:5, 1.0 mL/min, dr >99:1, τ_minor_ = 8.9 min τ_major_ = 10.9 min; OR: [α]^20^_D_ = +15.2 (c 1.20, CH_2_Cl_2_) mp 116–118 °C ^1^H NMR (400 MHz, CDCl_3_) δ = 7.87 (dd, *J* = 7.8, 0.7, 1H, CH_arom_), 7.48–7.19 (m, 10H, CH_arom_), 7.09 (dt, *J* = 6.2, 1.4, 1H, CH_arom_), 7.00 (d, *J* = 3.0, 1H, CH_arom_), 6.82–6.77 (m, 1H, CH_arom_), 5.51 (d, *J* = 11.9, 1H, CHNO_2_), 5.10 (d, *J* = 15.7, 1H, CHHPh), 5.00 (d, *J* = 15.7, 1H, CHHPh), 4.18 (t, *J* = 12.0, 1H, CHPh), 2.38 (td, *J* = 11.9, 3.0, 1H, CHCHPh), 2.27–2.18 (m, 1H, CHH), 1.97 (tt, *J* = 12.1, 3.4 Hz, 1H, CHH) 1.93–1.80 (m, 1H, CHH), 1.76–1.49 (m, 4H, CHH, CH_2_, OH), 1.32–1.15 (m, 2H, CH_2_) ppm. ^13^C{1H} NMR (101 MHz, CDCl_3_) δ = 173.3, 142.3, 134.9, 133.5, 132.2, 131.3, 129.5, 129.2, 129.1, 128.7, 128.3, 127.4, 125.3, 124.8, 124.6, 121.6, 113.0, 111.2, 110.3, 92.4, 78.5, 54.7, 53.4, 50.4, 45.9, 45.6, 43.1, 37.1, 25.2, 20.8 ppm. HR-MS (ESI^+^) *m*/*z* calcd. for [M + H]^+^ = [C_32_H_29_N_4_O_4_]^+^: 533.2183, found 533.2182.

#### (3*S*,3′*R*,4′*R*,4a′*S*,8a′*S*)-1-Benzyl-5-chloro-8a′-hydroxy-3′-nitro-2-oxo-4′-phenyl-3′,4′,4a′,5′,6′,7′,8′,8a′-octahydro-1′*H*-spiro[indoline-3,2′-naphthalene]-1′,1′-dicarbonitrile (**5c**)

The product **5c** was isolated after flash chromatography on silica gel (H/EtOAc 6:4) as a colorless solid (11 mg, 19% yield, >99:1 dr, 92% ee). Molecular formula: C_32_H_27_ClN_4_O_4_. Molecular mass: 567.04 g mol^–1^. HPLC: IA, hexane/iPrOH 95:5, 1.0 mL/min, dr >99:1, τ_minor_ = 10.5 min τ_major_ = 12.8 min; OR: [α]^20^_D_ = +8.5 (c 1.1, CH_2_Cl_2_) mp 190–192 °C ^1^H NMR (400 MHz, CDCl_3_) δ = 7.87 (d, *J* = 2.0, 1H, CH_arom_), 7.49–7.26 (m, 9H, CH_arom_), 7.11–7.07 (m, 1H, CH_arom_), 6.86 (d, *J* = 3.1, 1H, CH_arom_), 6.71 (d, *J* = 8.5, 1H, CH_arom_), 5.46 (d, *J* = 11.9, 1H, CHNO_2_), 5.07 (d, *J* = 15.7, 1H, CHHPh), 4.99 (d, *J* = 15.7, 1H, CHHPh), 4.16 (t, *J* = 11.9, 1H, CHPh), 2.38 (td, *J* = 11.9, 3.0, 1H, CHCHPh), 2.27–2.19 (m, 1H, CHH), 1.98 (tt, *J* = 12.1, 3.6 Hz, 1H, CHH), 1.93–1.80 (m, 1H, CHH), 1.74–1.50 (m, 4H, CHH, CH_2_, OH), 1.32–1.15 (m, 2H, CH_2_) ppm. ^13^C{1H} NMR (101 MHz, CDCl_3_) δ = 172.9, 140.9, 134.6, 133.1, 132.3, 131.3, 130.5, 129.5, 129.3, 129.2, 128.8, 128.5, 125.8, 124.6, 123.2, 112.7, 112.3, 110.1, 92.2, 92.2, 78.6, 54.7, 50.2, 45.8, 45.7, 43.0, 37.1, 29.7, 25.2, 25.2, 20.8 ppm. HR-MS (ESI^+^) *m*/*z* calcd. for [M – H]^−^ = [C_32_H_26_N_4_O_4_Cl]^−^: 565.1648, found 565.1648.

#### (3*S*,3′*R*,4′*R*,4a′*S*,8a′*S*)-1-Benzyl-5-bromo-8a′-hydroxy-3′-nitro-2-oxo-4′-phenyl-3′,4′,4a′,5′,6′,7′,8′,8a′-octahydro-1′*H*-spiro[indoline-3,2′-naphthalene]-1′,1′-dicarbonitrile (**5d**)

The product **5d** was isolated after flash chromatography on silica gel (H/EtOAc 6:4) as a colorless solid (8 mg, 13% yield, >99:1 dr, 92% ee). Molecular formula: C_32_H_27_BrN_4_O_4_. Molecular mass: 611.50 g mol^–1^. HPLC: IA, hexane/iPrOH 95:5, 1.0 mL/min, dr >99:1, τ_minor_ = 12.0 min τ_major_ = 13.9 min; OR: [α]^20^_D_ = +6.9 (c 0.8, CH_2_Cl_2_) mp 194–196 °C ^1^H NMR (400 MHz, CDCl_3_) δ = 8.00 (d, *J* = 1.9, 1H, CH_arom_), 7.50–7.25 (m, 9H, CH_arom_), 7.08 (dd, *J* = 5.2, 3.6, 1H, CH_arom_), 6.84 (d, *J* = 3.1, 1H, CH_arom_), 6.66 (d, *J* = 8.5, 1H, CH_arom_), 5.46 (d, *J* = 11.9, 1H, CHNO_2_), 5.06 (d, *J* = 15.7, 1H, CH_2_Ph), 4.99 (d, *J* = 15.7, 1H, CH_2_Ph), 4.15 (t, *J* = 12.0, 1H, CHPh), 2.38 (td, *J* = 11.9, 3.0, 1H, CHCHPh), 2.26–2.19 (m, 1H, CHH), 1.97 (tt, *J* = 12.2, 3.6 Hz, 1H, CHH), 1.92–1.80 (m, 1H, CHH), 1.76–1.49 (m, 4H, CHH, CH_2_, OH), 1.31–1.16 (m, 2H, CH_2_) ppm. ^13^C{1H} NMR (101 MHz, CDCl_3_) δ = 172.8, 141.4, 135.2, 134.6, 133.0, 131.3, 129.5, 129.3, 128.8, 128.5, 128.5, 127.4, 124.6, 123.5, 117.6, 112.7, 112.7, 110.0, 92.2, 78.6, 54.6, 50.2, 50.2, 45.8, 45.7, 43.0, 37.1, 25.2, 25.1, 20.8 ppm. HR-MS (ESI^+^) *m*/*z* calcd. for [M – H]^−^ = [C_32_H_26_N_4_O_4_Cl]^−^: 565.1648, found 565.1648.

#### (3*S*,3′*R*,4′*R*,4a′*S*,8a′*S*)-1-Benzyl-8a′-hydroxy-5-methyl-3′-nitro-2-oxo-4′-phenyl-3′,4′,4a′,5′,6′,7′,8′,8a′-octahydro-1′*H*-spiro[indoline-3,2′-naphthalene]-1′,1′-dicarbonitrile (**5e**)

The product **5e** was isolated after flash chromatography on silica gel (H/EtOAc 6:4) as a colorless solid (9 mg, 17% yield, >99:1 dr, 92% ee). Molecular formula: C_33_H_30_N_4_O_4_. Molecular mass: 546.63 g mol^–1^. HPLC: IA, hexane/iPrOH 95:5, 1.0 mL/min, dr >99:1, τ_minor_ = 8.8 min τ_major_ = 10.8 min; OR: [α]^20^_D_ = +2 (c 1.00, CH_2_Cl_2_) mp 110–112 °C ^1^H NMR (300 MHz, CDCl_3_) δ = 7.67 (d, *J* = 1.4 Hz, 1H), 7.47–7.27 (m, 13H), 7.16–7.05 (m, 3H), 6.67 (d, *J* = 8.1 Hz, 1H), 5.49 (d, *J* = 12.0 Hz, 1H), 5.07 (d, *J* = 15.8 Hz, 1H), 4.98 (d, *J* = 15.6 Hz, 1H), 4.18 (t, *J* = 11.9 Hz, 1H), 2.36 (s, 4H), 2.22 (d, *J* = 11.0 Hz, 1H), 2.04–1.81 (m, 2H), 1.66 (d, *J* = 15.0 Hz, 2H), 1.28 (s, 3H) ppm. ^13^C{1H} NMR (101 MHz, CDCl_3_) δ = 173.2, 139.9, 135.0, 134.8, 133.7, 132.5, 131.3, 129.4, 129.0, 128.7, 128.2, 127.4, 127.1, 125.9, 124.6, 121.6, 111.0, 110.4, 92.4, 82.3, 78.4, 54.7, 50.5, 47.3, 45.9, 45.5, 43.1, 37.1, 31.6, 29.7, 25.2, 21.3, 20.8 ppm. HR-MS (ESI^+^) *m*/*z* calcd. for [M + H]^+^ = [C_33_H_31_N_4_O_4_]^+^: 547.2340, found 547.2338.

#### (3*S*,3′*R*,4′*R*,4a′*S*,8a′*S*)-8a′-Hydroxy-1-methyl-3′-nitro-2-oxo-4′-phenyl-3′,4′,4a′,5′,6′,7′,8′,8a′-octahydro-1′*H*-spiro[indoline-3,2′-naphthalene]-1′,1′-dicarbonitrile (**5f**)

The product **5f** was isolated after flash chromatography on silica gel (H/EtOAc 6:4) as a colorless solid (36 mg, 79% yield, >99:1 dr, 92% ee). Molecular formula: C_26_H_24_N_4_O_4_. Molecular mass: 456.50 g mol^–1^. HPLC: IA, hexane/iPrOH 95:5, 1.0 mL/min, dr >99:1, τ_minor_ = 10.2 min τ_major_ = 12.5 min; OR: [α]^20^_D_ = −18.6 (c 1.0, CH_2_Cl_2_) mp 166–168 °C ^1^H NMR (300 MHz, CDCl_3_) δ = 7.90–7.85 (m, 1H, CH_arom_), 7.53–7.23 (m, 5H, CH_arom_), 7.06 (dt, *J* = 6.6, 1.6, 1H, CH_arom_), 7.00 (d, *J* = 2.9, 1H, CH_arom_), 6.95 (d, *J* = 7.9, 1H, CH_arom_), 5.47 (d, *J* = 11.9, 1H, CHNO_2_), 4.13 (t, *J* = 12.0, 1H, CHPh), 3.36 (s, 3H, CH_3_), 2.35 (td, *J* = 11.7, 3.0, 1H, CHCHPh), 2.24–2.15 (m, 1H, CHH), 2.01–1.90 (m, 1H, CHH), 1.89–1.77 (m, 2H, CH_2_), 1.75–1.46 (m, 7H, 3xCH_2_, OH) ppm. ^13^C{1H} NMR (75 MHz, CDCl_3_) δ = 172.5, 142.5, 134.4, 131.8, 130.8, 129.0, 128.8, 128.2, 124.9, 124.4, 124.2, 121.2, 112.5, 109.7, 109.7, 109.5, 91.9, 77.9, 54.3, 49.8, 45.4, 42.5, 36.7, 31.5, 29.3, 24.8, 22.3, 20.4, 13.7 ppm. HR-MS (ESI^+^) *m*/*z* calcd. for [M + H]^+^ = [C_26_H_25_N_4_O_4_]^+^: 457.1870, found 457.1874.

#### (3*S*,3′*R*,4′*R*,4a′*S*,8a′*S*)-5-Chloro-8a′-hydroxy-1-methyl-3′-nitro-2-oxo-4′-phenyl-3′,4′,4a′,5′,6′,7′,8′,8a′-octahydro-1′*H*-spiro[indoline-3,2′-naphthalene]-1′,1′-dicarbonitrile (**5g**)

The product **5g** was isolated after flash chromatography on silica gel (H/EtOAc 6:4) as a colorless solid (30 mg, 63% yield, >99:1 dr, 92% ee). Molecular formula: C_26_H_23_ClN_4_O_4_. Molecular mass: 490.94 g mol^–1^. HPLC: IA, hexane/iPrOH 95:5, 1.0 mL/min, dr >99:1, τ_minor_ = 12.9 min τ_major_ = 15.0 min; OR: [α]^20^_D_ = −10.3 (c 1.2, CH_2_Cl_2_) mp 120–122 °C ^1^H NMR (300 MHz, CDCl_3_) δ = 7.87 (d, *J* = 1.9, 1H, CH_arom_), 7.51–7.24 (m, 4H, CH_arom_), 7.05 (dt, *J* = 7.2, 1.7, 1H, CH_arom_), 6.90 (d, *J* = 8.4, 1H, CH_arom_), 6.85 (d, *J* = 3.0, 1H, CH_arom_), 5.42 (d, *J* = 11.9, 1H, CHNO_2_), 4.10 (t, *J* = 12.0, 1H, CHPh), 3.35 (s, 3H, CH_3_), 2.34 (td, *J* = 11.9, 2.9, 1H, CHCHPh), 2.24–2.15 (m, 1H, CHH), 2.01–1.90 (m, 1H, CHH), 1.88–1.77 (m, 2H, CH_2_), 1.75–1.45 (m, 7H, 3xCH_2_, OH) ppm. ^13^C{1H} NMR (75 MHz, CDCl_3_) δ = 172.1, 141.1, 134.2, 131.9, 130.8, 130.1, 129.1, 128.8, 128.4, 125.4, 124.2, 122.8, 112.3, 110.6, 109.5, 91.8, 78.0, 54.3, 49.6, 45.4, 42.5, 36.7, 29.3, 27.3, 24.7, 24.7, 20.3 ppm. HR-MS (ESI^+^) *m*/*z* calcd. for [M + H]^+^ = [C_26_H_24_N_4_O_4_Cl]^+^: 491.1481, found 491.1481.

#### (3*S*,3′*R*,4′*R*,4a′*S*,8a′*S*)-8a′-Hydroxy-1,5-dimethyl-3′-nitro-2-oxo-4′-phenyl-3′,4′,4a′,5′,6′,7′,8′,8a′-octahydro-1′*H*-spiro[indoline-3,2′-naphthalene]-1′,1′-dicarbonitrile (**5h**)

The product **5h** was isolated after flash chromatography on silica gel (H/EtOAc 6:4) as a colorless solid (19 mg, 40% yield, >99:1 dr, 92% ee). Molecular formula: C_27_H_26_N_4_O_4_. Molecular mass: 470.53 g mol^–1^. HPLC: IA, hexane/iPrOH 95:5, 1.0 mL/min, dr >99:1, τ_minor_ = 9.2 min τ_major_ = 10.5 min; OR: [α]^20^_D_ = −6.2 (c 0.9, CH_2_Cl_2_) mp 108–110 °C ^1^H NMR (300 MHz, CDCl_3_) δ = 7.71–7.64 (m, 1H, CH_arom_), 7.50–7.28 (m, 4H, CH_arom_), 7.11–7.00 (m, 2H, CH_arom_), 6.83 (d, *J* = 8.0 Hz, 1H, CH_arom_), 5.45 (d, *J* = 11.9 Hz, 1H, CHNO_2_), 4.13 (t, *J* = 12.0 Hz, 1H, CHPh), 3.33 (s, 3H, NMe), 2.44–2.29 (m, 4H, Me - CHH), 2.19 (d, *J* = 10.7 Hz, 1H, CHH), 2.04–1.79 (m, 2H, CH_2_), 1.75–1.46 (m, 6H, 2xCH_2_ and OH) ppm. ^13^C{1H} NMR (75 MHz, CDCl_3_) δ = 172.4, 140.1, 134.6, 134.4, 132.2, 130.8, 129.0, 128.8, 128.5, 128.2, 125.5, 124.2, 121.2, 112.5, 109.8, 109.4, 92.0, 77.9, 54.3, 49.9, 45.4, 42.6, 36.6, 29.3, 27.2, 24.8, 20.9, 20.4 ppm. HR-MS (ESI^+^) *m*/*z* calcd. for [M + H]^+^ = [C_27_H_27_N_4_O_4_]^+^: 471.2027, found 471.2016.

#### (3*S*,3′*R*,4′*R*,4a′*S*,8a′*S*)-1-Ethyl-8a′-hydroxy-3′-nitro-2-oxo-4′-phenyl-3′,4′,4a′,5′,6′,7′,8′,8a′-octahydro-1′*H*-spiro[indoline-3,2′-naphthalene]-1′,1′-dicarbonitrile (**5i**)

The product **5i** was isolated after flash chromatography on silica gel (H/EtOAc 6:4) as a white/rose solid (38 mg, 40% yield, >99:1 dr, 92% ee). Molecular formula: C_27_H_26_N_4_O_4_. Molecular mass: 470.53 g mol^–1^. HPLC: IB, hexane/iPrOH 95:5, 1.0 mL/min, dr >99:1, τ_minor_ = 8.2 min τ_major_ = 9.1 min; OR: [α]^20^_D_ = −10.7 (c 0.75, CH_2_Cl_2_) mp 218–220 °C. ^1^H NMR (300 MHz, CDCl3) δ = 7.87 (d, *J* = 7.7, 1H), 7.53–7.36 (m, 3H), 7.33–7.22 (m, 3H), 7.11–7.04 (m, 2H), 6.96 (d, *J* = 8.0, 1H), 5.47 (d, *J* = 11.9, 1H), 4.13 (t, *J* = 12.0, 1H), 3.88 (dt, *J* = 11.8, 7.1, 2H), 2.35 (td, *J* = 11.9, 2.9, 1H), 2.19 (d, *J* = 10.6, 1H), 2.03–1.75 (m, 2H), 1.67 (t, *J* = 15.7, 3H), 1.54 (d, *J* = 15.2, 1H), 1.33 (t, *J* = 7.2, 3H), 1.26 (s, 1H), 1.17 (d, *J* = 12.0, 2H). ^13^C{1H} NMR (101 MHz, CDCl_3_) δ = 171.4, 146.8, 134.5, 131.8, 130.8, 129.0, 128.7, 128.7, 128.2, 128.1, 125.1, 124.2, 112.5, 109.7, 109.6, 91.9, 77.9, 45.4, 42.5, 36.7, 35.9, 35.3, 32.8, 28.6, 24.8, 20.4, 11.3 ppm. HR-MS (ESI^+^) *m*/*z* calcd. for [M + H]^+^ = [C_27_H_27_N_4_O_4_]^+^: 471.2027, found 471.2010.

#### (3*S*,3′*R*,4′*R*,4a′*S*,8a′*S*)-5-Chloro-1-ethyl-8a′-hydroxy-3′-nitro-2-oxo-4′-phenyl-3′,4′,4a′,5′,6′,7′,8′,8a′-octahydro-1′*H*-spiro[indoline-3,2′-naphthalene]-1′,1′-dicarbonitrile (**5j**)

The product **5j** was isolated after flash chromatography on silica gel (H/EtOAc 6:4) as a white/rose solid (23 mg, 22% yield, >99:1 dr, 92% ee). Molecular formula: C_27_H_25_ClN_4_O_4_. Molecular mass: 504.97 g mol^–1^. HPLC: IB, hexane/iPrOH 95:5, 1.0 mL/min, dr >99:1, τ_minor_ = 7.8 min τ_major_ = 9.8 min; OR: [α]^20^_D_ = −6.0 (c 0.5, CH_2_Cl_2_) mp 222–224 °C. ^1^H NMR (300 MHz, CDCl3) δ = 7.87 (d, *J* = 2.0, 1H), 7.47 (dd, *J* = 8.4, 1.9, 1H), 7.41 (t, *J* = 6.7, 1H), 7.33–7.26 (m, 2H), 7.06 (d, *J* = 7.1, 1H), 6.91 (t, *J* = 6.1, 2H), 5.42 (d, *J* = 11.9, 1H), 4.11 (t, *J* = 12.0, 1H), 3.87 (qd, *J* = 7.1, 3.0, 2H), 2.34 (td, *J* = 11.9, 3.1, 1H), 2.19 (d, *J* = 11.6, 1H), 1.94 (d, *J* = 12.0, 1H), 1.62 (d, *J* = 34.1, 4H), 1.32 (t, *J* = 7.3, 3H), 1.26 (s, 2H), 1.20 (d, *J* = 1.6, 1H) ppm. ^13^C{1H} NMR (101 MHz, CDCl_3_) δ = 171.8, 140.2, 134.2, 131.9, 130.8, 130.3, 129.8, 129.1, 128.8, 128.4, 128.3, 125.6, 124.2, 112.3, 110.6, 91.8, 78.0, 53.9, 49.5, 45.3, 42.5, 36.7, 36.1, 29.2, 24.7, 20.3, 11.2 ppm. HR-MS (ESI^+^) *m*/*z* calcd. for [M + H]^+^ = [C_27_H_26_N_4_O_4_Cl]^+^: 505.1637, found 505.1634.

#### (3*S*,3′*R*,4′*R*,4a′*S*,8a′*S*)-1-Ethyl-8a′-hydroxy-5-methyl-3′-nitro-2-oxo-4′-phenyl-3′,4′,4a′,5′,6′,7′,8′,8a′-octahydro-1′*H*-spiro[indoline-3,2′-naphthalene]-1′,1′-dicarbonitrile (**5k**

The product **5k** was isolated after flash chromatography on silica gel (H/EtOAc 6:4) as a white solid (26 mg, 27% yield, >99:1 dr, 92% ee). Molecular formula: C_28_H_28_N_4_O_4_. Molecular mass: 484.56 g mol^–1^. HPLC: IB, hexane/iPrOH 95:5, 1.0 mL/min, dr >99:1, τ_minor_ = 6.4 min τ_major_ = 7.5 min; OR: [α]^20^_D_ = −15.0 (c 0.73, CH_2_Cl_2_) mp 232–233 °C. ^1^H NMR (300 MHz, CDCl3) δ = 7.67 (s, 1H), 7.42 (s, 2H), 7.36–7.21 (m, 4H), 7.15 (d, *J* = 2.8, 1H), 7.07 (d, *J* = 6.9, 1H), 6.84 (d, *J* = 8.1, 1H), 5.45 (d, *J* = 11.9, 1H), 4.13 (t, *J* = 12.0, 1H), 3.86 (ddd, *J* = 14.2, 7.1, 2.9, 2H), 2.40 (s, 3H), 2.19 (d, *J* = 10.8, 1H), 1.89 (dd, *J* = 25.7, 12.3, 3H), 1.76–1.43 (m, 5H), 1.32 (t, *J* = 7.2, 4H), 1.25 (s, 3H), 1.19 (d, *J* = 2.9, 1H). ^13^C{1H} NMR (75 MHz, CDCl_3_) δ = 172.0, 139.2, 134.6, 134.2, 132.1, 130.8, 129.0, 128.7, 128.2, 125.7, 124.2, 121.5, 112.5, 109.7, 109.6, 109.4, 91.9, 77.9, 53.9, 49.9, 45.4, 42.6, 36.7, 35.9, 29.3, 24.8, 20.9, 20.4, 11.3 ppm. HR-MS (ESI^+^) *m*/*z* calcd. for [M + H]^+^ = [C_28_H_29_N_4_O_4_]^+^: 485.2183, found 485.2171.

## Supporting Information Available

^1^H, ^13^C{1H} NMR spectra for all compounds, ESI-MS spectra, and HPLC chromatograms (PDF)
