# Supplementary material for: Stereoselective Synthesis of Spiro-Decalin Oxindole Derivatives via Sequential Organocatalytic Michael–Domino Michael/Aldol Reaction
Source: J Org Chem. 2022 Jul 25;87(15):10454–61. doi: 10.1021/acs.joc.2c01046 (PMC9365295; doi:10.1021/acs.joc.2c01046)
Supplement: Supplementary file 1 — jo2c01046_si_001.pdf [file jo2c01046_si_001.pdf]

Supplementary Information

Stereoselective synthesis of spiro-decalin oxindole derivatives *via* sequential organocatalytic Michael–domino Michael/aldol reaction

Leonardo Straminelli,[1,§] Francesco Vicentini,[1,§] Antonio di Sabato,[1] Carmela Maria Montone,[1] Chiara Cavaliere,[1] Kari Rissanen,[2] Francesca Leonelli,[1] Fabrizio Vetica[1,*]

[1]*Department of Chemistry, Sapienza University of Rome, Piazzale Aldo Moro 5, 00185 Roma, Italy*

[2]*Department of Chemistry, University of Jyväskylä, 40014 Jyväskylä, Finland*

[§]*These authors contributed equally to the work.*

*email: fabrizio.vetica@uniroma1.it

Table of Contents

NMR SPECTRA, HPLC CHROMATOGRAMS AND HRMS SPECTRA **S2**

(3S,3'R,4'R,4a'S,8a'S)-1-(4-bromobenzyl)-8a'-hydroxy-3'-nitro-2-oxo-4'-phenyl-3',4',4a',5',6',7',8',8a'-octahydro-1'H-spiro[indoline-3,2'-naphthalene]-1',1'-dicarbonitrile (**5a**) S2

(3S,3'R,4'R,4a'S,8a'S)-1-benzyl-8a'-hydroxy-3'-nitro-2-oxo-4'-phenyl-3',4',4a',5',6',7',8',8a'-octahydro-1'H-spiro[indoline-3,2'-naphthalene]-1',1'-dicarbonitrile (**5b**) S6

(3S,3'R,4'R,4a'S,8a'S)-1-benzyl-5-chloro-8a'-hydroxy-3'-nitro-2-oxo-4'-phenyl-3',4',4a',5',6',7',8',8a'-octahydro-1'H-spiro[indoline-3,2'-naphthalene]-1',1'-dicarbonitrile (**5c**) S9

(3S,3'R,4'R,4a'S,8a'S)-1-benzyl-5-bromo-8a'-hydroxy-3'-nitro-2-oxo-4'-phenyl-3',4',4a',5',6',7',8',8a'-octahydro-1'H-spiro[indoline-3,2'-naphthalene]-1',1'-dicarbonitrile (**5d**) S12

(3S,3'R,4'R,4a'S,8a'S)-1-benzyl-8a'-hydroxy-5-methyl-3'-nitro-2-oxo-4'-phenyl-3',4',4a',5',6',7',8',8a'-octahydro-1'H-spiro[indoline-3,2'-naphthalene]-1',1'-dicarbonitrile (**5e**) S15

(3S,3'R,4'R,4a'S,8a'S)-8a'-hydroxy-1-methyl-3'-nitro-2-oxo-4'-phenyl-3',4',4a',5',6',7',8',8a'-octahydro-1'H-spiro[indoline-3,2'-naphthalene]-1',1'-dicarbonitrile (**5f**) S18

(3S,3'R,4'R,4a'S,8a'S)-5-chloro-8a'-hydroxy-1-methyl-3'-nitro-2-oxo-4'-phenyl-3',4',4a',5',6',7',8',8a'-octahydro-1'H-spiro[indoline-3,2'-naphthalene]-1',1'-dicarbonitrile (**5g**) S21

(3S,3'R,4'R,4a'S,8a'S)-8a'-hydroxy-1,5-dimethyl-3'-nitro-2-oxo-4'-phenyl-3',4',4a',5',6',7',8',8a'-octahydro-1'H-spiro[indoline-3,2'-naphthalene]-1',1'-dicarbonitrile (**5h**) S24

(3S,3'R,4'R,4a'S,8a'S)-1-ethyl-8a'-hydroxy-3'-nitro-2-oxo-4'-phenyl-3',4',4a',5',6',7',8',8a'-octahydro-1'H-spiro[indoline-3,2'-naphthalene]-1',1'-dicarbonitrile (**5i**) S27

(3S,3'R,4'R,4a'S,8a'S)-5-chloro-1-ethyl-8a'-hydroxy-3'-nitro-2-oxo-4'-phenyl-3',4',4a',5',6',7',8',8a'-octahydro-1'H-spiro[indoline-3,2'-naphthalene]-1',1'-dicarbonitrile (**5j**) S30

(3S,3'R,4'R,4a'S,8a'S)-1-ethyl-8a'-hydroxy-5-methyl-3'-nitro-2-oxo-4'-phenyl-3',4',4a',5',6',7',8',8a'-octahydro-1'H-spiro[indoline-3,2'-naphthalene]-1',1'-dicarbonitrile (**5k**) S33

NMR Spectra, HPLC Chromatograms and HRMS Spectra

(3S,3'R,4'R,4a'S,8a'S)-1-(4-bromobenzyl)-8a'-hydroxy-3'-nitro-2-oxo-4'-phenyl-3',4',4a',5',6',7',8',8a'-octahydro-1'H-spiro[indoline-3,2'-naphthalene]-1',1'-dicarbonitrile (5a)

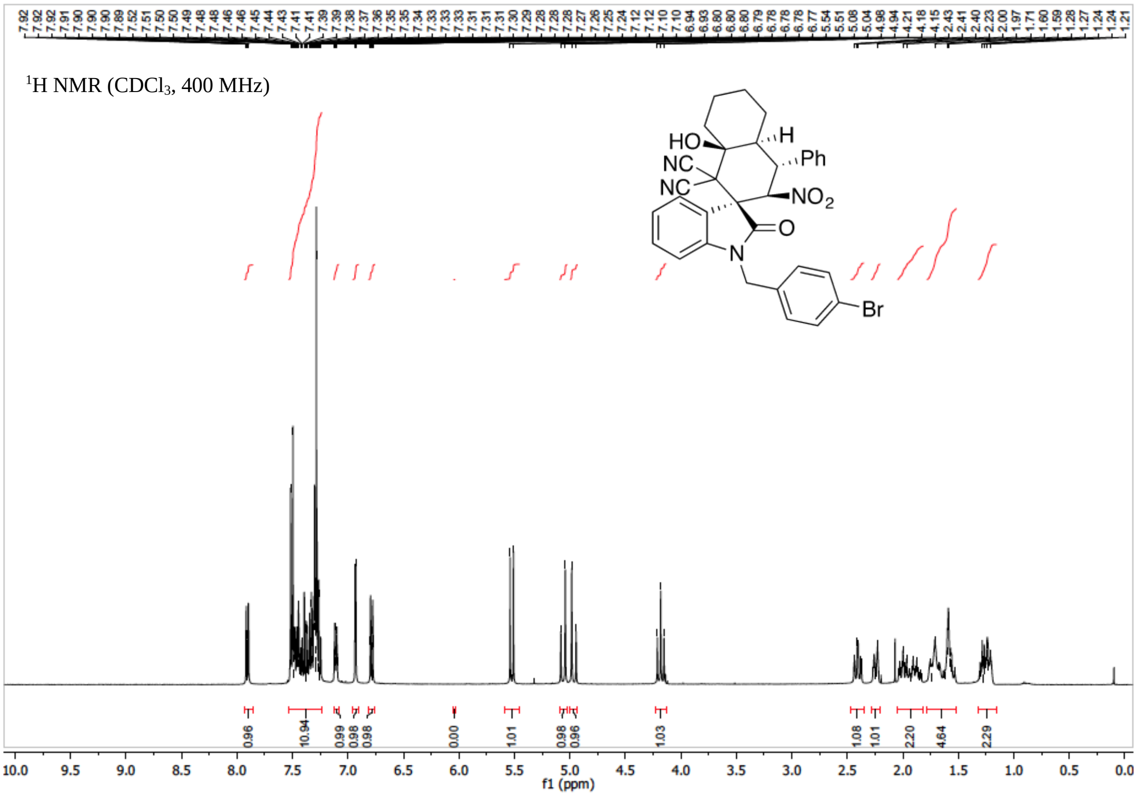

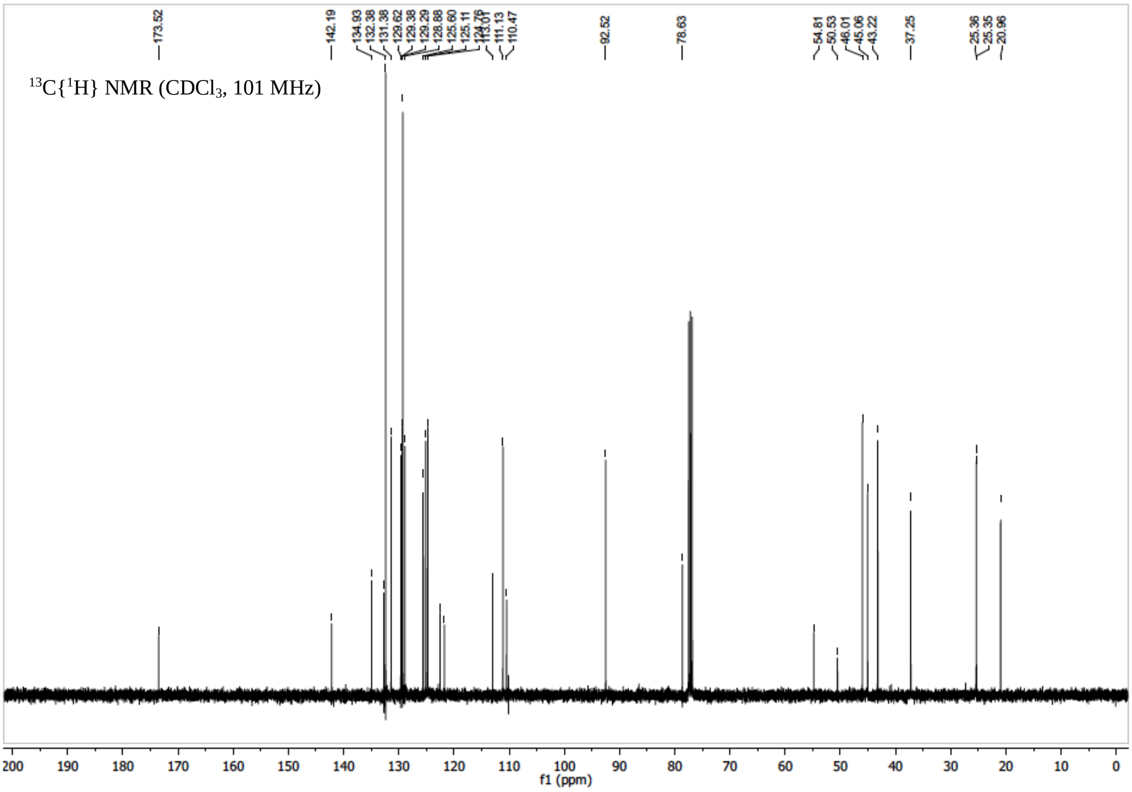

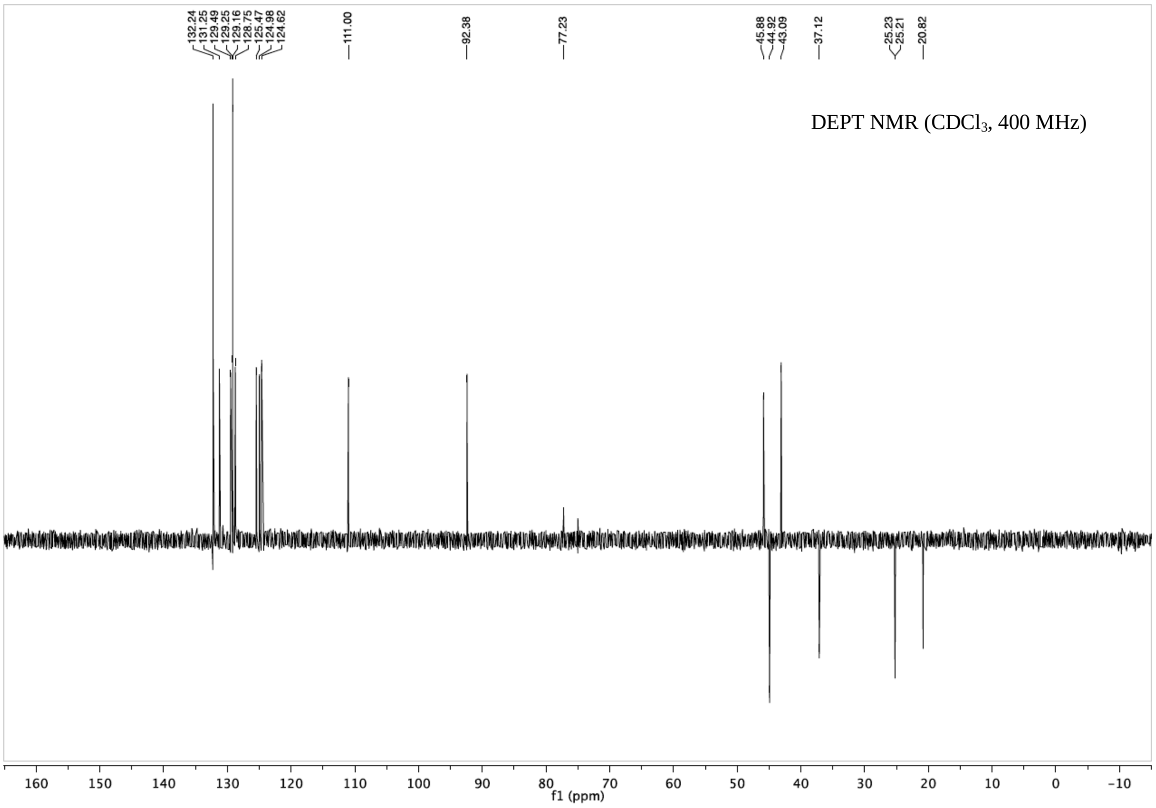
132.24
131.25
129.49
129.25
129.16
128.75
125.47
124.98
124.62
111.00
92.38
77.23
45.88
44.92
43.09
37.12
25.23
25.21
20.82
DEPT NMR ($CDCl_3$, 400 MHz)
160 150 140 130 120 110 100 90 80 70 60 50 40 30 20 10 0 -10
f1 (ppm)

HSQC

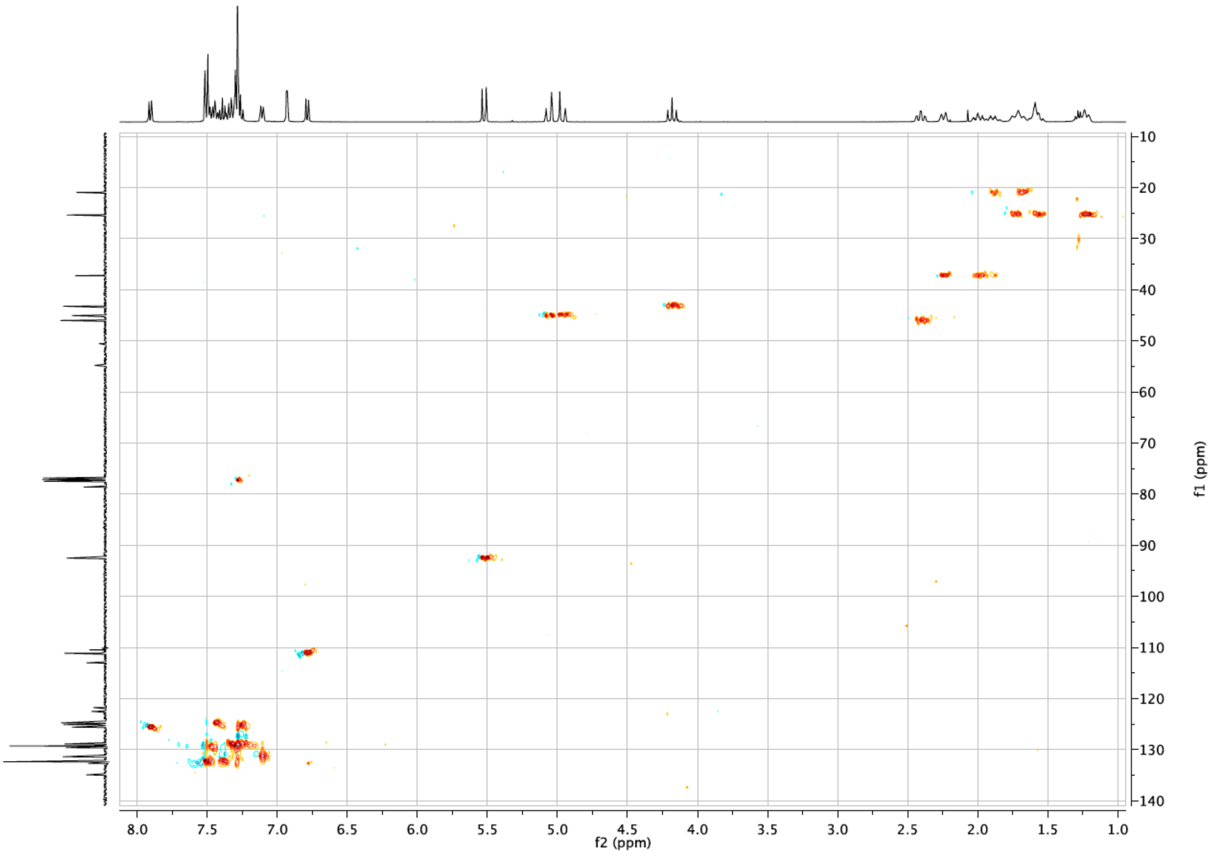
8.0 7.5 7.0 6.5 6.0 5.5 5.0 4.5 4.0 3.5 3.0 2.5 2.0 1.5 1.0
f2 (ppm)
10 20 30 40 50 60 70 80 90 100 110 120 130 140
f1 (ppm)

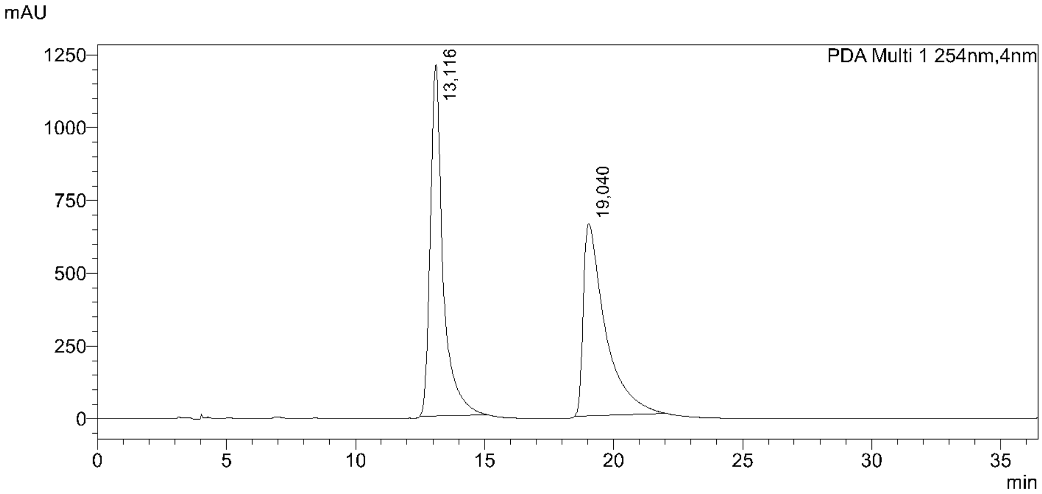

PDA Ch1 254nm

Peak#	Ret. Time	Area	Height	Conc.	Unit	Mark	Name
1	13,116	41243019	1206820	51,187		M	
2	19,040	39329827	660092	48,813		M	
Total		80572846	1866912				

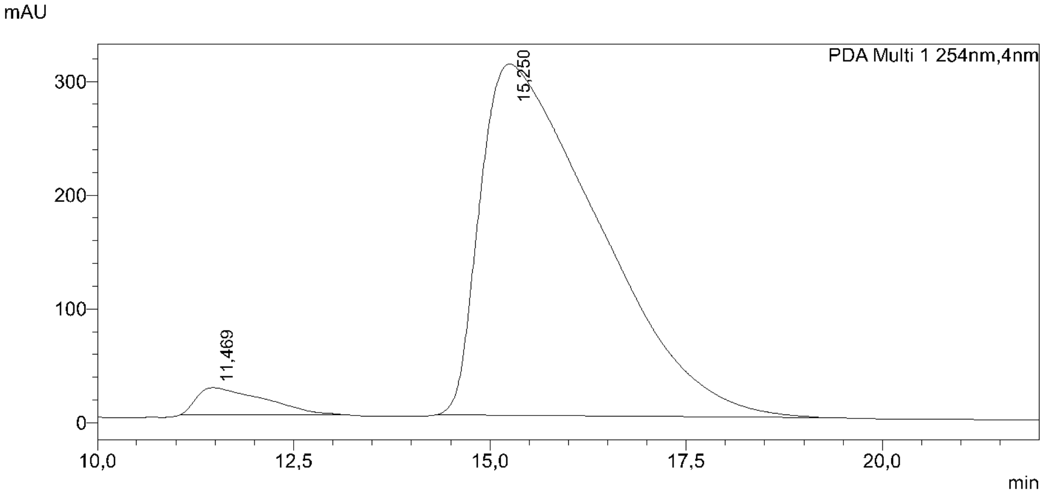

PDA Ch1 254nm

Peak#	Ret. Time	Area	Height	Conc.	Unit	Mark	Name
1	11,469	1387260	23620	4,098		M	
2	15,250	32461677	308595	95,902		M	
Total		33848936	332215				

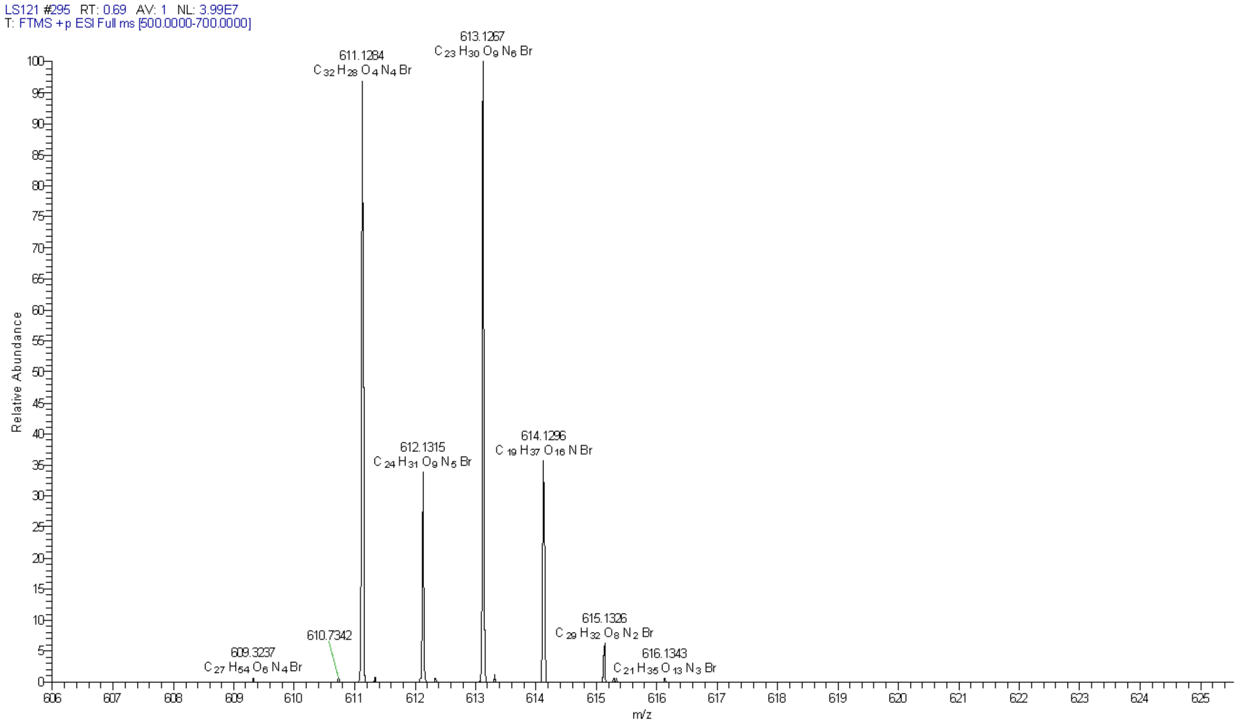

LS121 #295 RT: 0.69 AV: 1 NL: 3.99E7
T: FTMS + p ESI Full ms [500.0000-700.0000]
611.1284
C 32 H 28 O 4 N 4 Br
613.1267
C 23 H 30 O 9 N 6 Br
612.1315
C 24 H 31 O 9 N 5 Br
614.1296
C 19 H 37 O 16 N Br
615.1326
C 29 H 32 O 8 N 2 Br
616.1343
C 21 H 35 O 13 N 3 Br
609.3237
C 27 H 54 O 6 N 4 Br
610.7342
Relative Abundance
m/z

(3S,3'R,4'R,4a'S,8a'S)-1-benzyl-8a'-hydroxy-3'-nitro-2-oxo-4'-phenyl-3',4',4a',5',6',7',8',8a'-octahydro-1'H-spiro[indoline-3,2'-naphthalene]-1',1'-dicarbonitrile (5b)

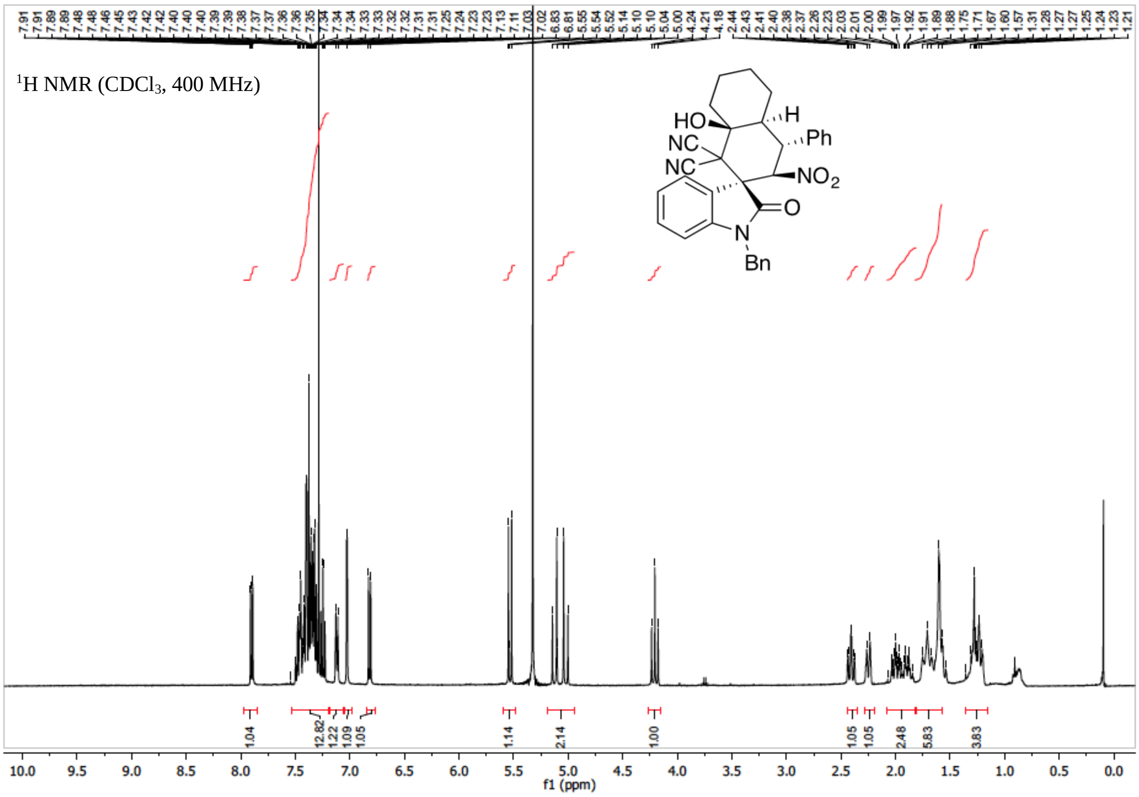

.

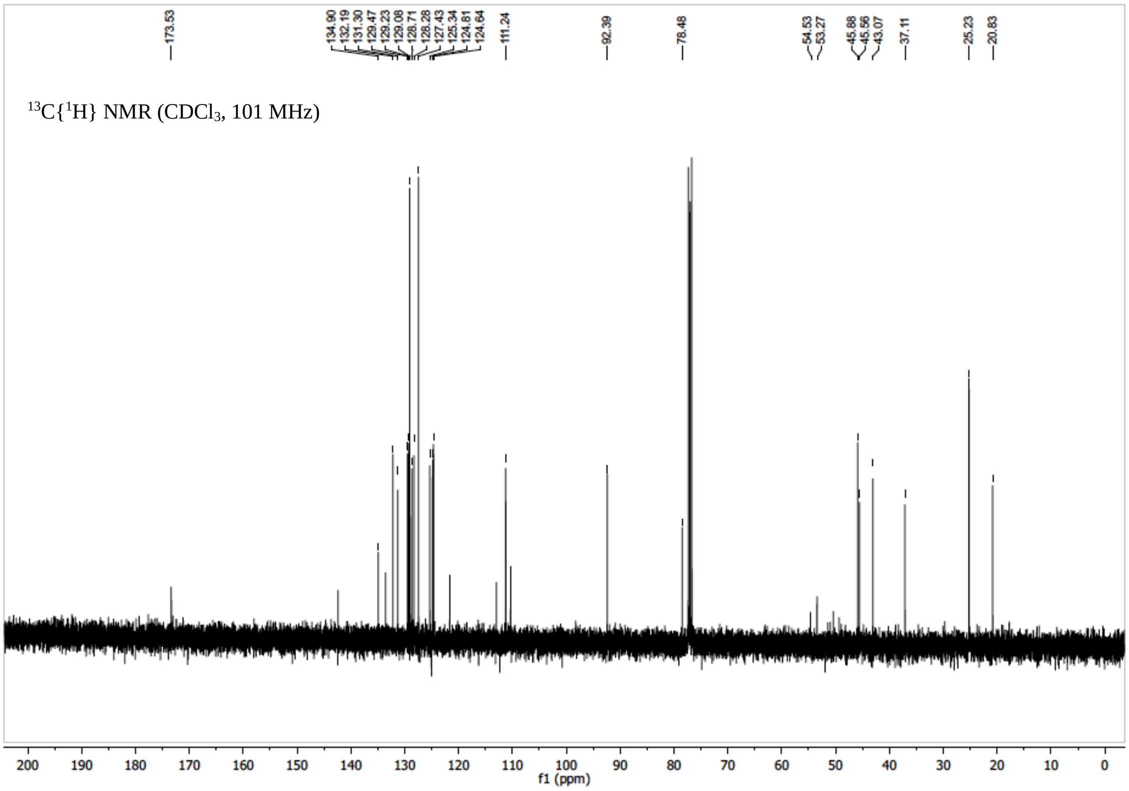

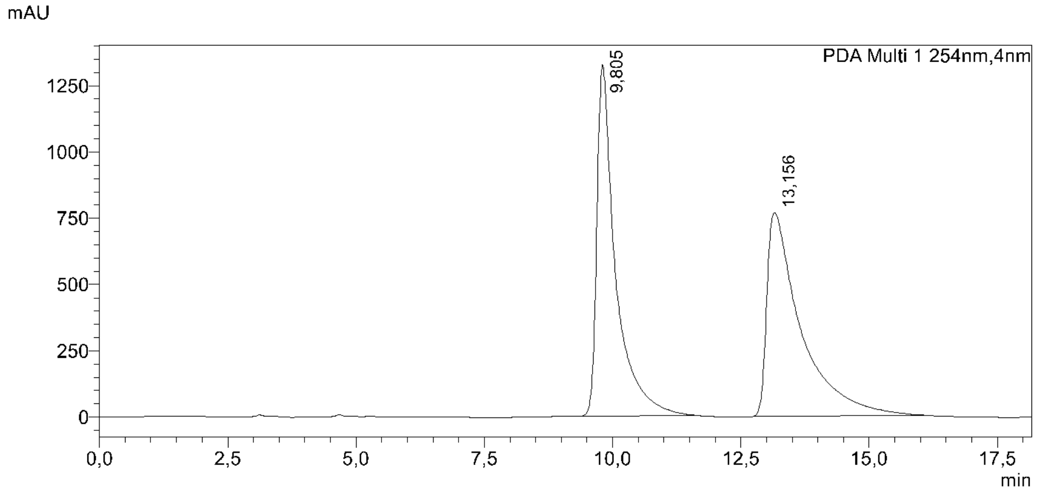

PDA Ch1 254nm

Peak#	Ret. Time	Area	Height	Conc.	Unit	Mark	Name
1	9,805	33585958	1324829	48,597		M	
2	13,156	35525057	769282	51,403		M	
Total		69111015	2094112				

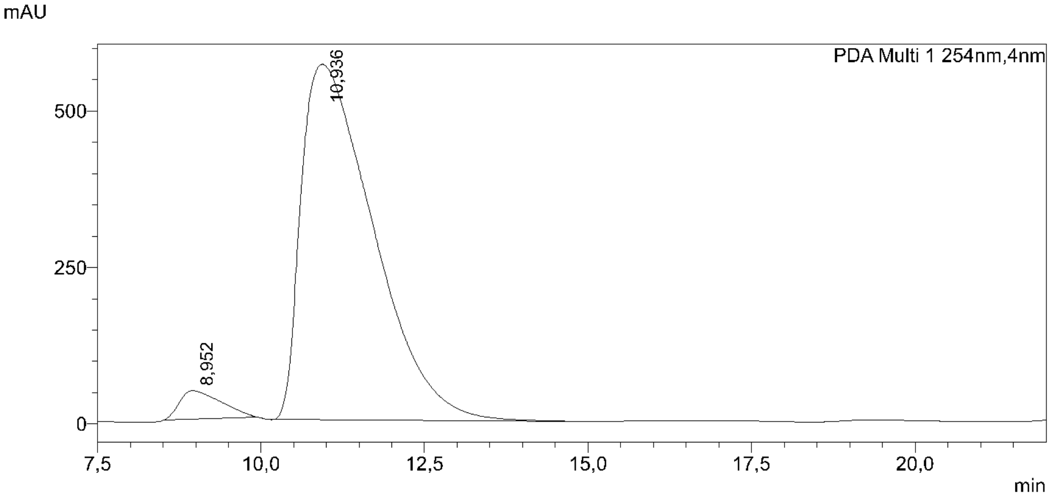

PDA Ch1 254nm

Peak#	Ret. Time	Area	Height	Conc.	Unit	Mark	Name
1	8,952	2024020	45852	4,426		M	
2	10,936	43703724	567601	95,574		M	
Total		45727745	613452				

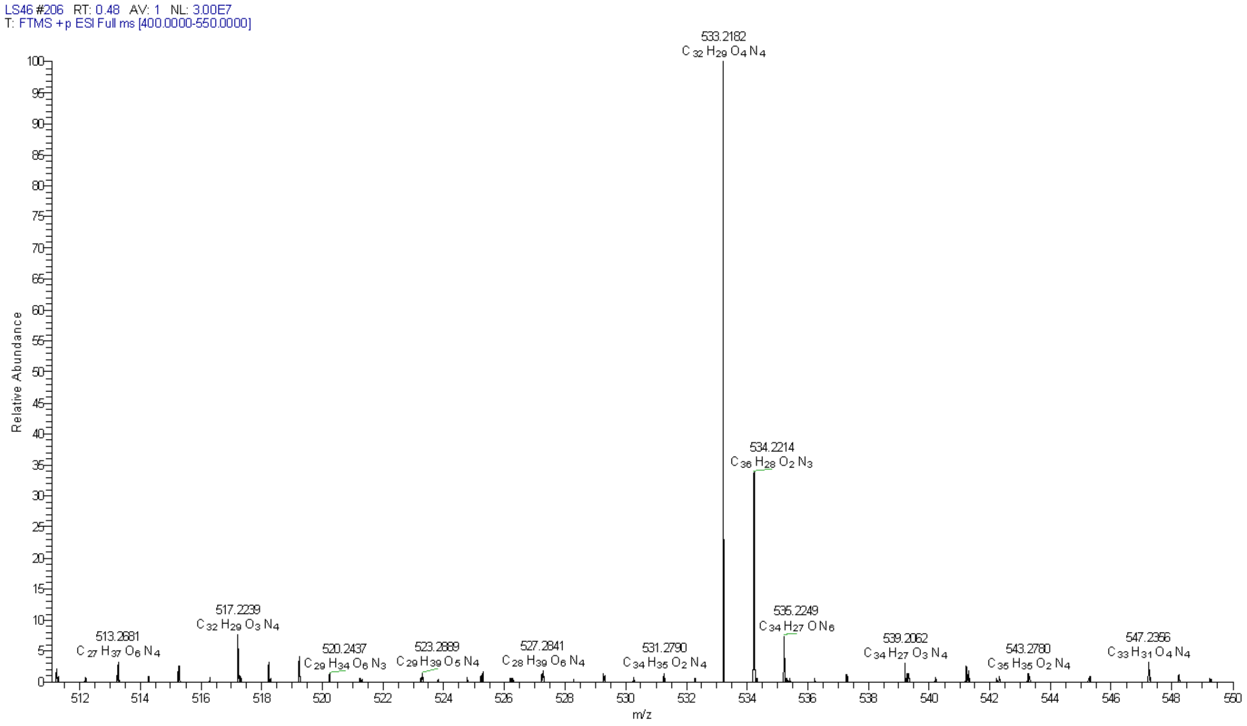
LS46 #206 RT: 0.48 AV: 1 NL: 3.00E7
T: FTMS + p ESI Full ms [400.0000-550.0000]
533.2182
C 32 H29 O4 N4
534.2214
C36 H28 O2 N3
535.2249
C34 H27 O N6
517.2239
C32 H29 O3 N4
513.2681
C27 H37 O6 N4
520.2437
C29 H34 O6 N3
523.2889
C29 H39 O5 N4
527.2841
C28 H39 O6 N4
531.2790
C34 H35 O2 N4
539.2062
C34 H27 O3 N4
543.2780
C35 H35 O2 N4
547.2366
C33 H31 O4 N4
Relative Abundance
m/z

(3S,3'R,4'R,4a'S,8a'S)-1-benzyl-5-chloro-8a'-hydroxy-3'-nitro-2-oxo-4'-phenyl-3',4',4a',5',6',7',8',8a'-octahydro-1'H-spiro[indoline-3,2'-naphthalene]-1',1'-dicarbonitrile (5c)

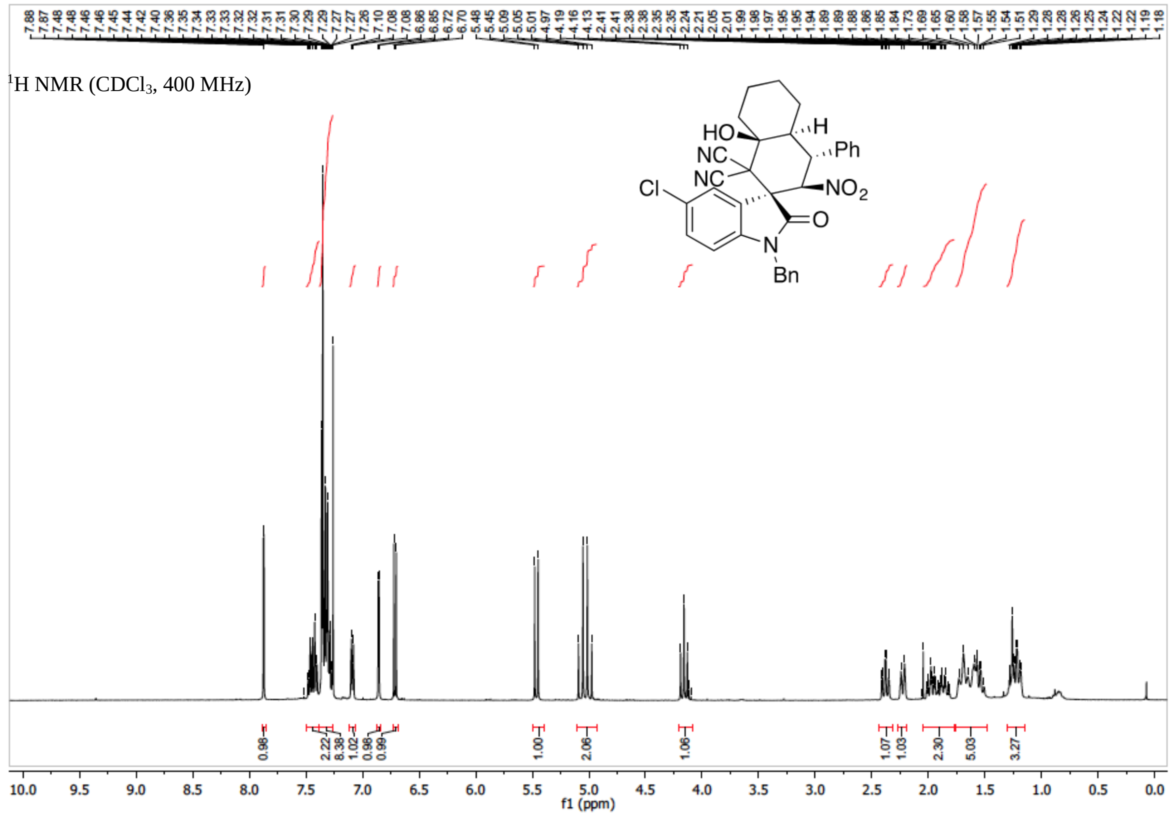

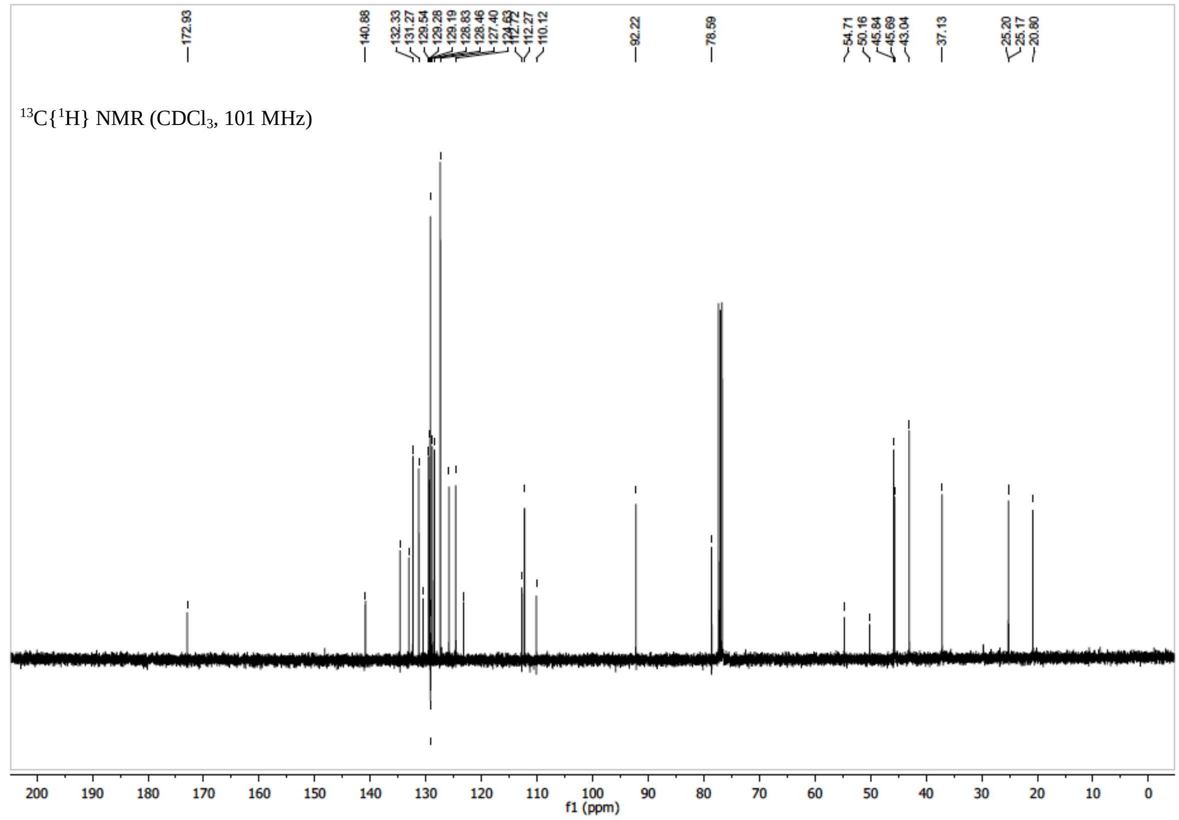

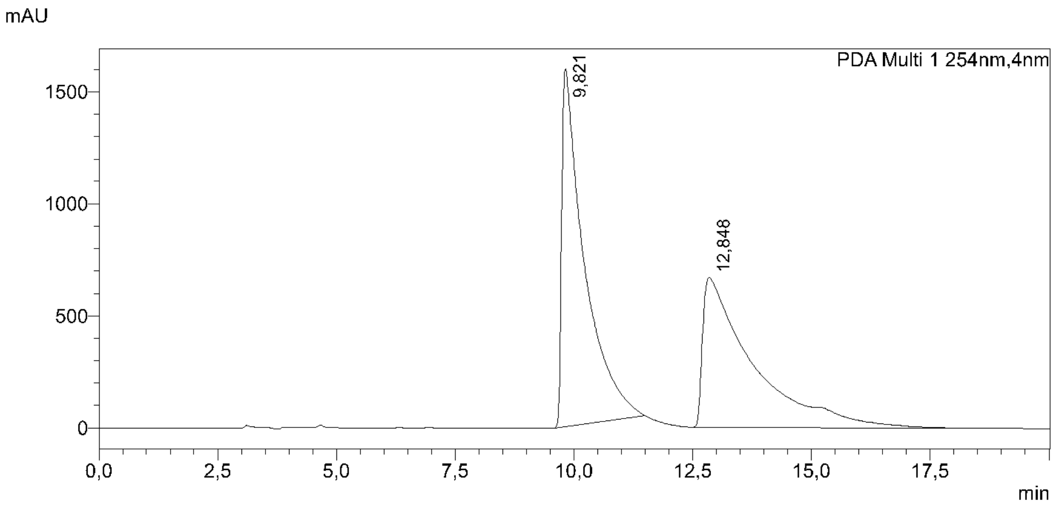

PDA Ch1 254nm

Peak#	Ret. Time	Area	Height	Conc.	Unit	Mark	Name
1	9,821	52685758	1596186	51,850		M	
2	12,848	48925444	667175	48,150		M	
Total		101611202	2263361				

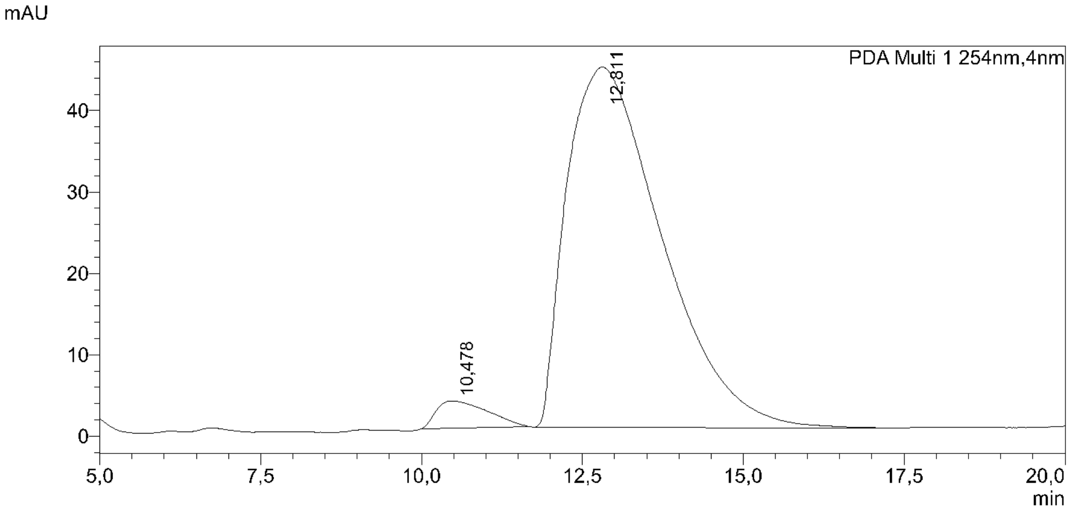

PDA Ch1 254nm

Peak#	Ret. Time	Area	Height	Conc.	Unit	Mark	Name
1	10,478	187162	3360	3,952		M	
2	12,811	4548302	44276	96,048		M	
Total		4735464	47635				

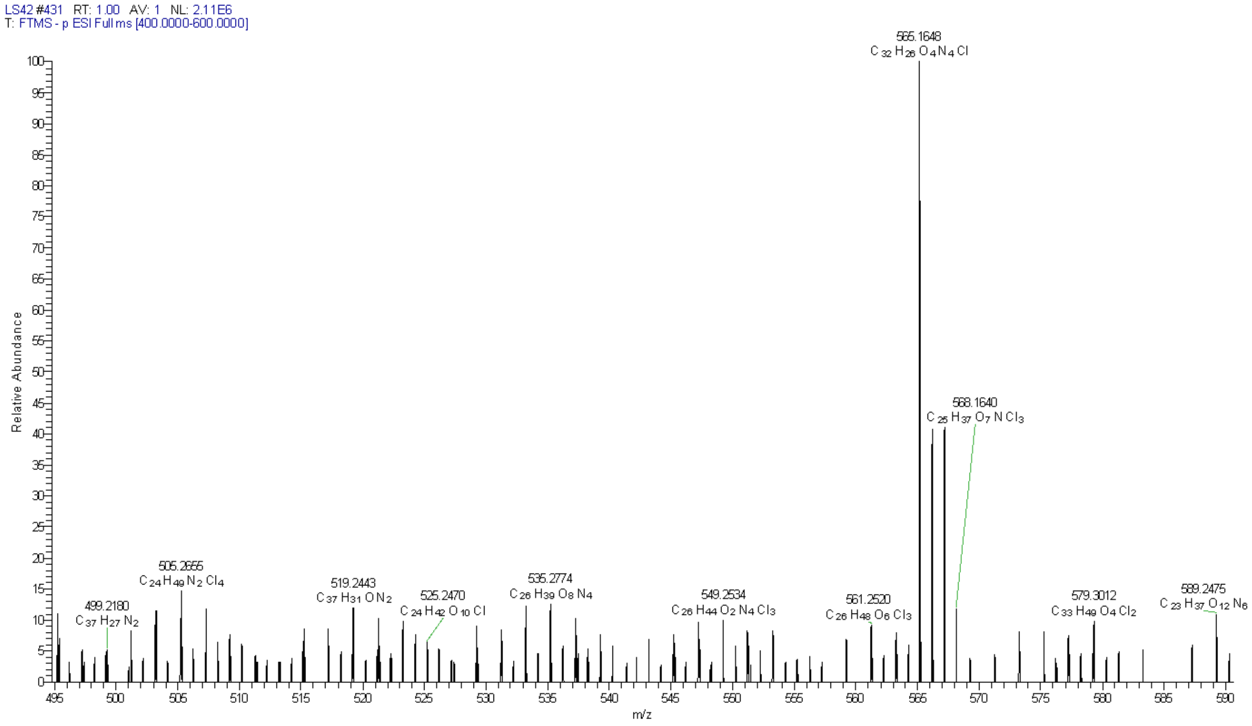
LS42 #431 RT: 1.00 AV: 1 NL: 2.11E6
T: FTMS - p ESI Full ms [400.0000-600.0000]
565.1648
C 32 H 26 O 4 N 4 Cl
568.1640
C 25 H 37 O 7 N Cl 3
505.2655
C 24 H 49 N 2 Cl 4
499.2180
C 37 H 27 N 2
519.2443
C 37 H 31 O N 2
525.2470
C 24 H 42 O 10 Cl
535.2774
C 26 H 39 O 8 N 4
549.2534
C 26 H 44 O 2 N 4 Cl 3
561.2520
C 26 H 48 O 6 Cl 3
579.3012
C 33 H 49 O 4 Cl 2
589.2475
C 23 H 37 O 12 N 6
Relative Abundance
m/z

(3S,3'R,4'R,4a'S,8a'S)-1-benzyl-5-bromo-8a'-hydroxy-3'-nitro-2-oxo-4'-phenyl-3',4',4a',5',6',7',8',8a'-octahydro-1'H-spiro[indoline-3,2'-naphthalene]-1',1'-dicarbonitrile (5d)

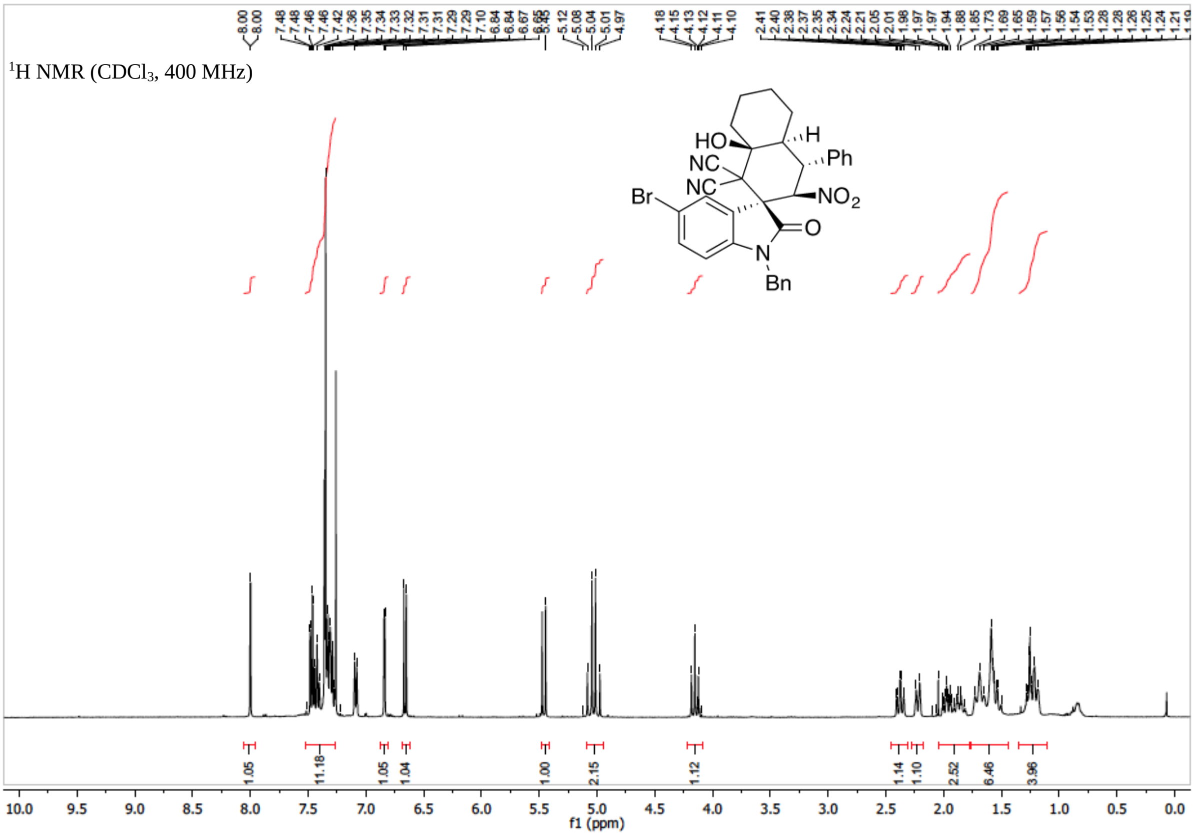

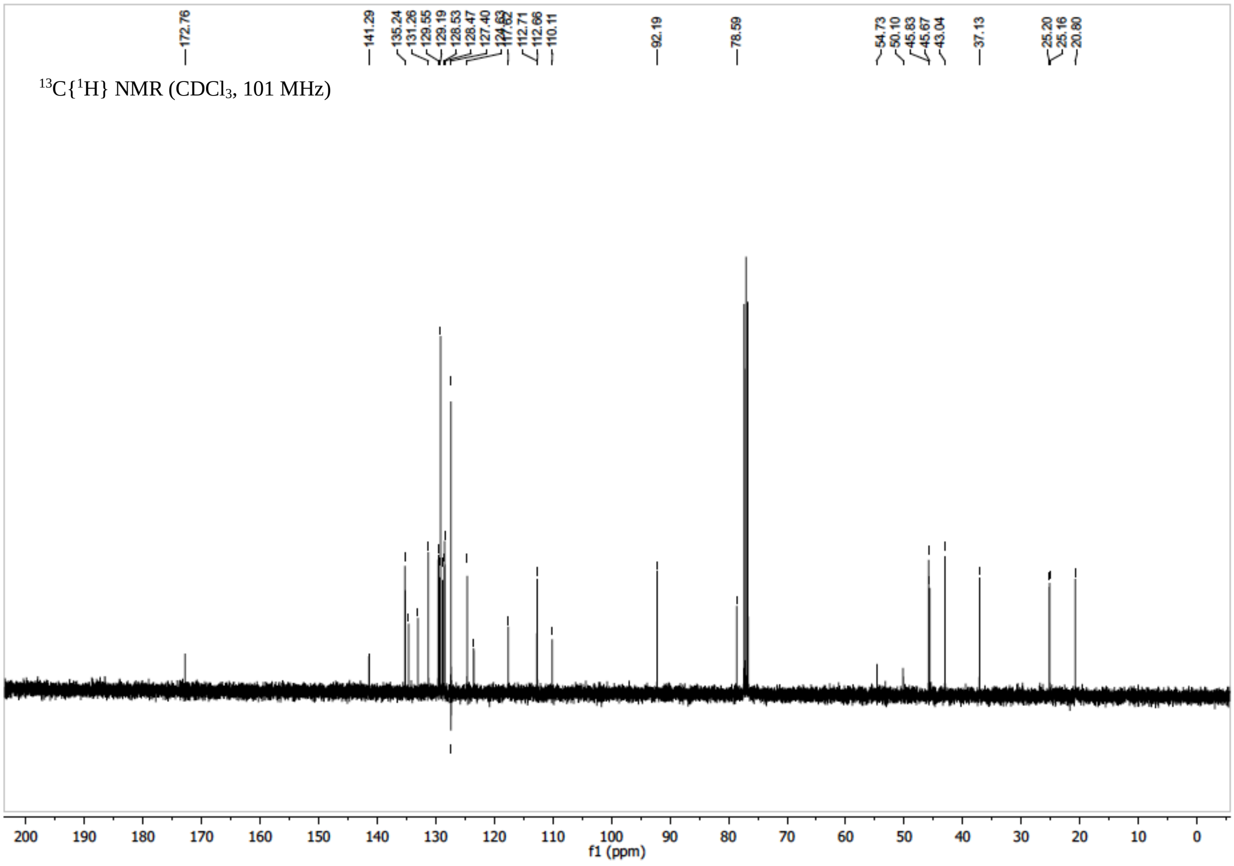

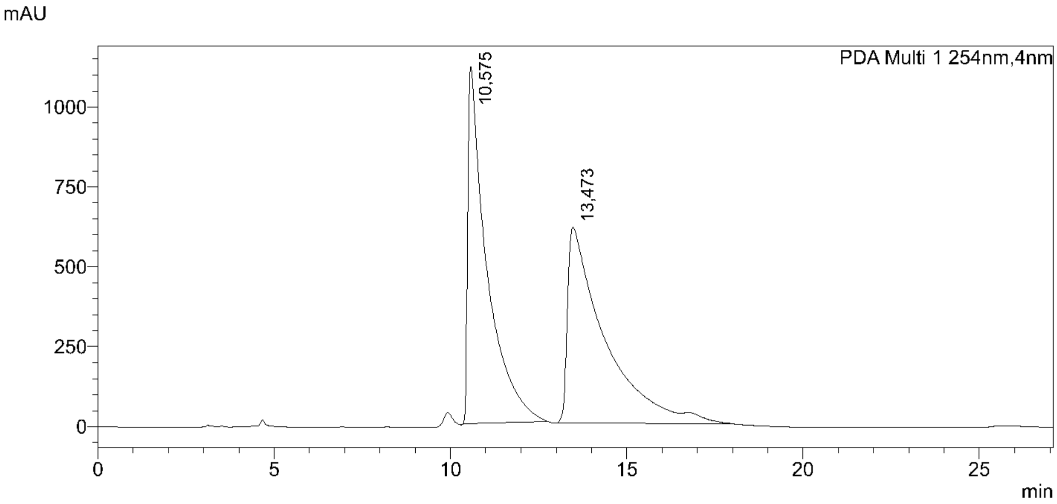

PDA Ch1 254nm

Peak#	Ret. Time	Area	Height	Conc.	Unit	Mark	Name
1	10,575	41662998	1117514	48,076		M	
2	13,473	44996923	612734	51,924		M	
Total		86659921	1730248				

<Chromatogram>

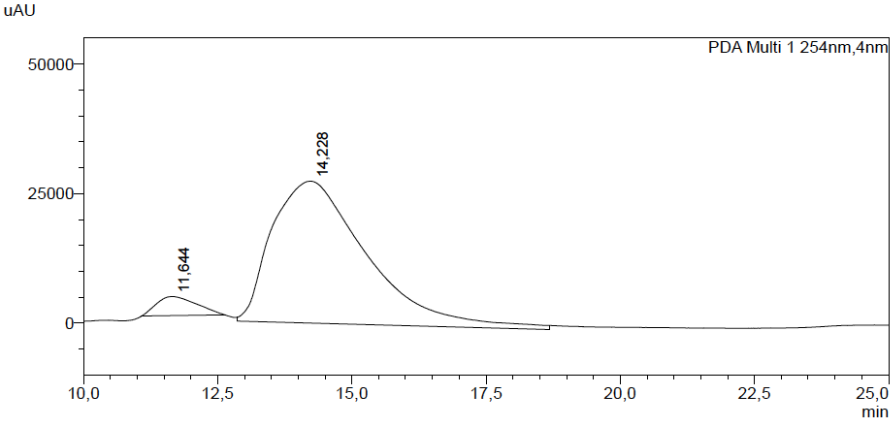

<Peak Table>

PDA Ch1 254nm

Peak#	Ret. Time	Area	Height	Area%
1	11,644	191403	3666	5,353
2	14,228	3384015	27399	94,647
Total		3575418	31066	100,000

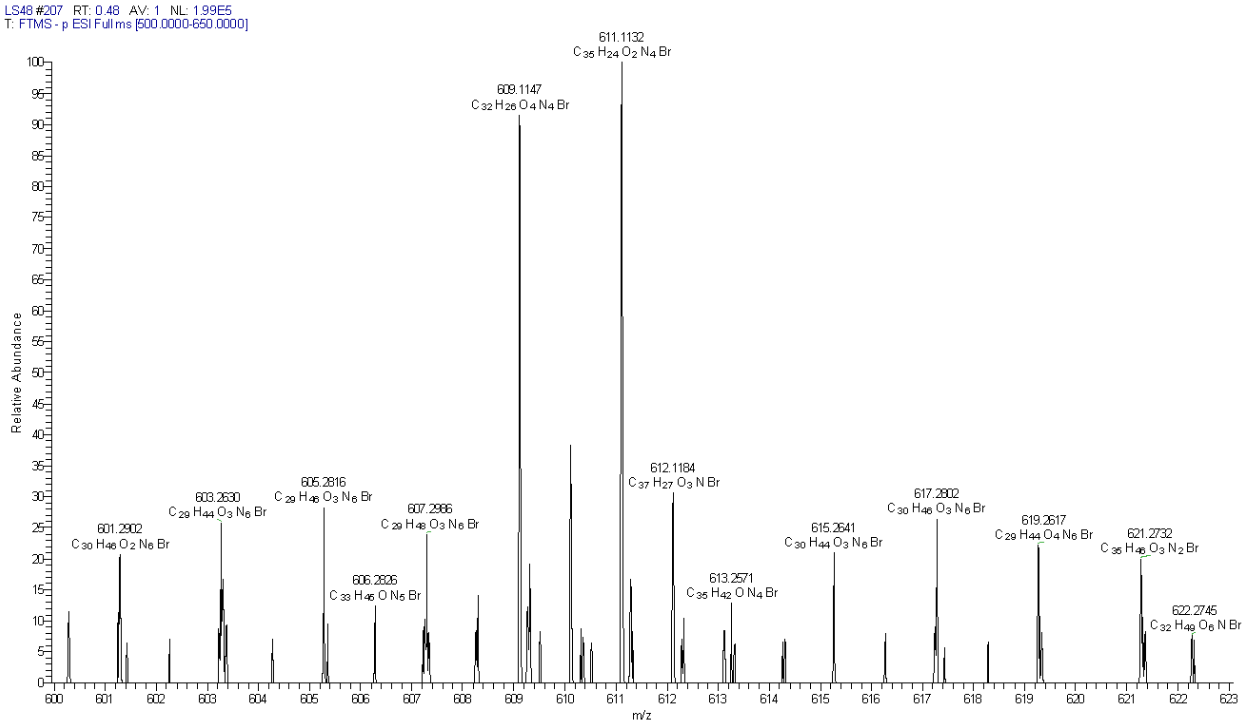

LS48 #207 RT: 0.48 AV: 1 NL: 1.99E5
T: FTMS - p ESI Full ms [500.0000-650.0000]
Relative Abundance
m/z
611.1132
C35 H24 O2 N4 Br
609.1147
C32 H26 O4 N4 Br
612.1184
C37 H27 O3 N Br
605.2816
C29 H46 O3 N6 Br
603.2630
C29 H44 O3 N6 Br
601.2902
C30 H46 O2 N6 Br
607.2986
C29 H48 O3 N6 Br
606.2826
C33 H46 O N5 Br
613.2571
C35 H42 O N4 Br
615.2641
C30 H44 O3 N6 Br
617.2802
C30 H46 O3 N6 Br
619.2617
C29 H44 O4 N6 Br
621.2732
C35 H46 O3 N2 Br
622.2745
C32 H49 O6 N Br

(3S,3'R,4'R,4a'S,8a'S)-1-benzyl-8a'-hydroxy-5-methyl-3'-nitro-2-oxo-4'-phenyl-3',4',4a',5',6',7',8',8a'-octahydro-1'H-spiro[indoline-3,2'-naphthalene]-1',1'-dicarbonitrile (5e)

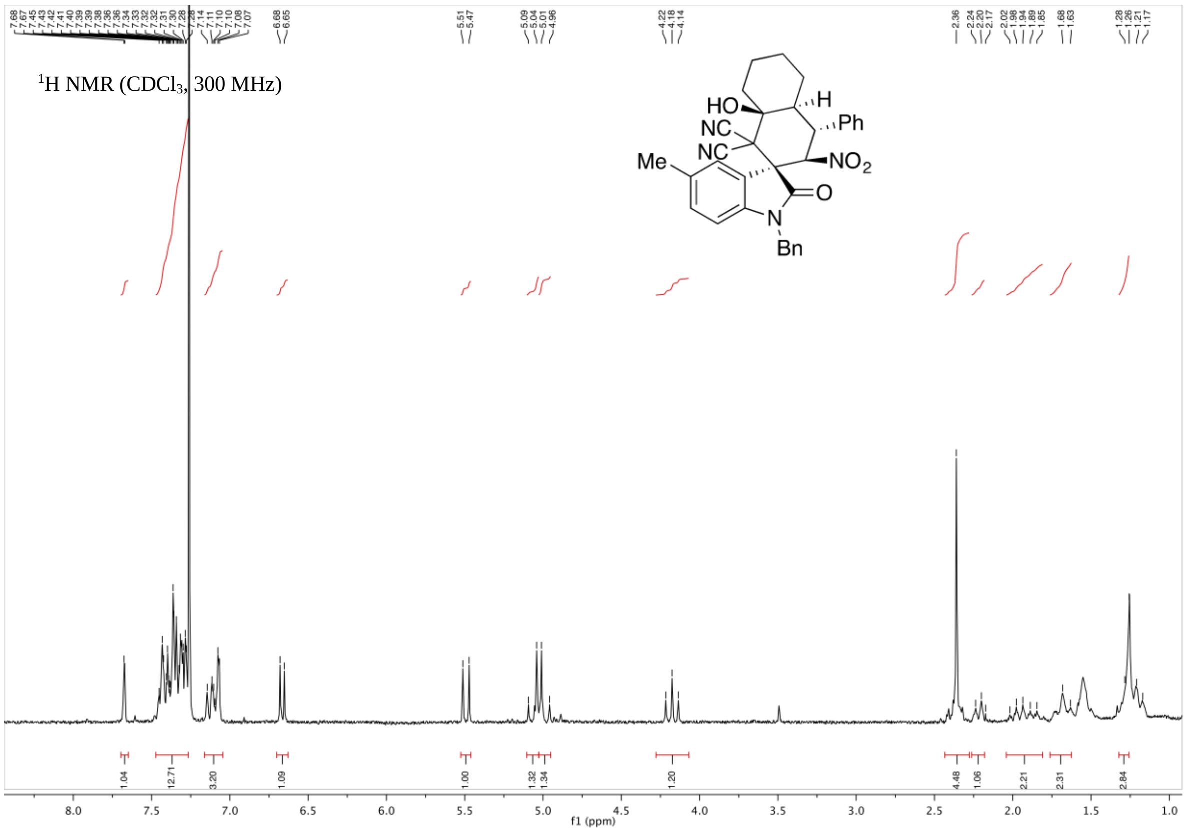

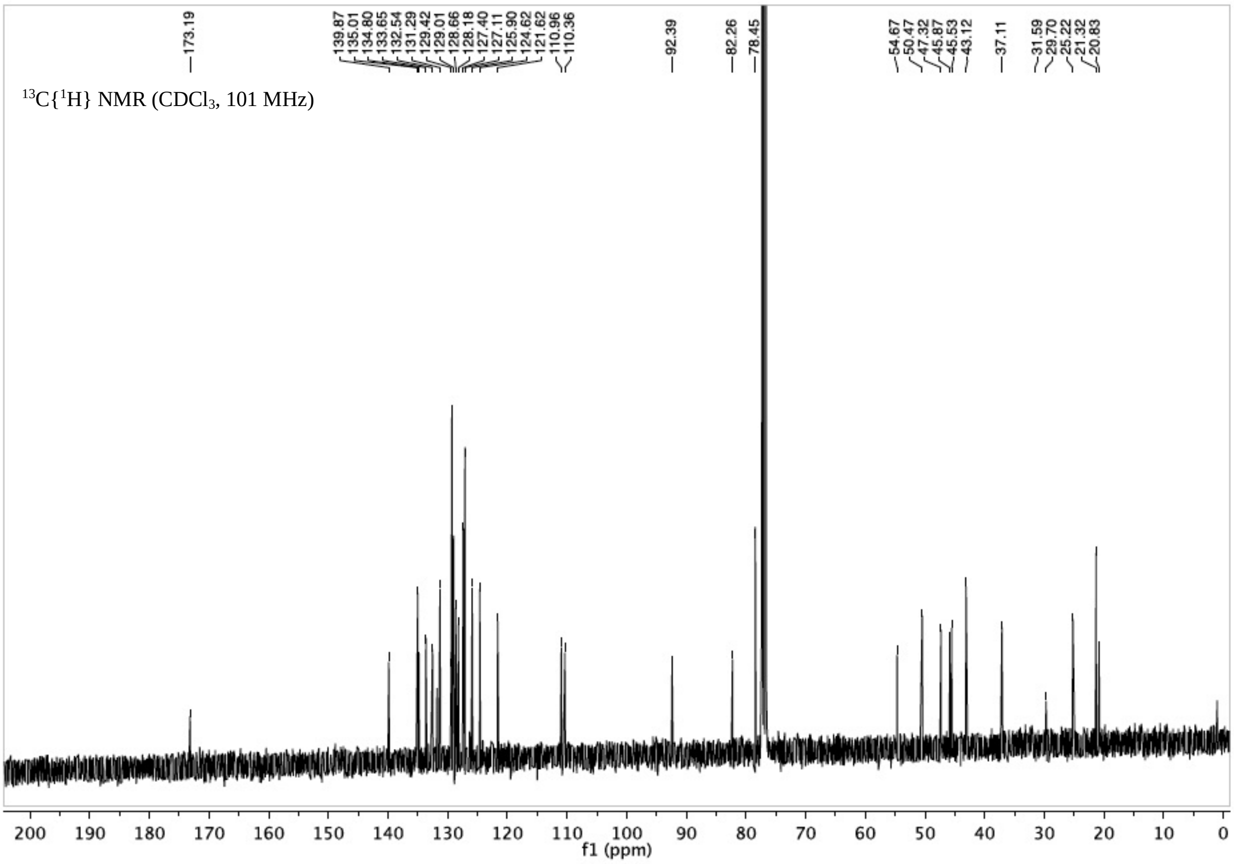

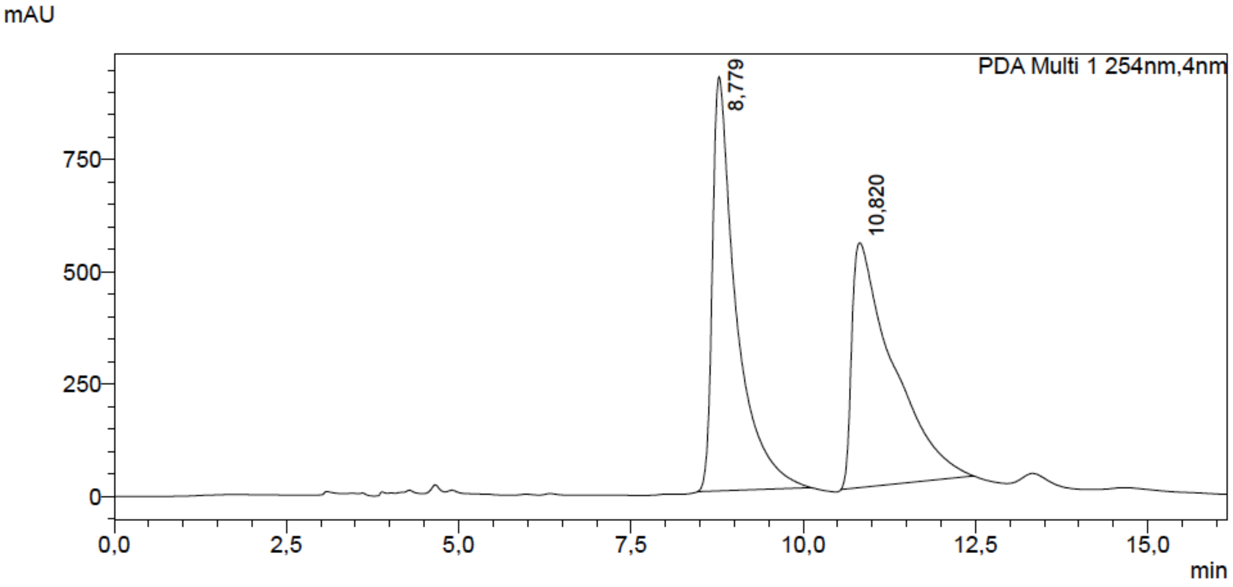

PDA Ch1 254nm

Peak#	Ret. Time	Area	Height	Conc.	Unit	Mark	Name
1	8,779	22163078	922671	49,397		M	
2	10,820	22704417	545795	50,603		M	
Total		44867496	1468466				

<Chromatogram>

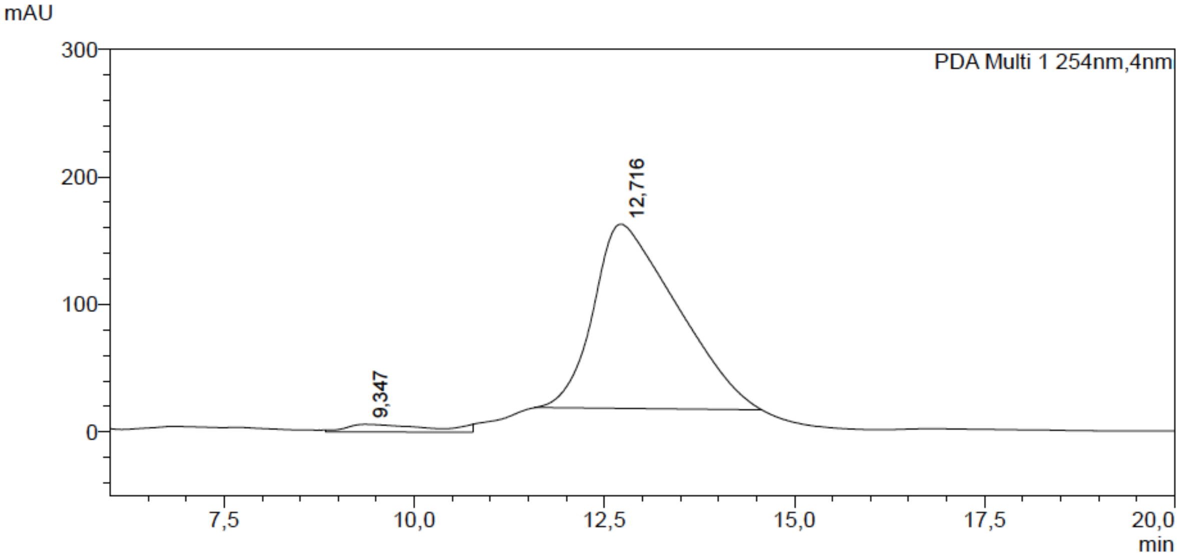

<Peak Table>

PDA Ch1 254nm

Peak#	Ret. Time	Area	Height	Conc.	Unit	Mark	Name
1	9,347	472637	6004	4,089		M	
2	12,716	11085423	144679	95,911		M	
Total		11558060	150683				

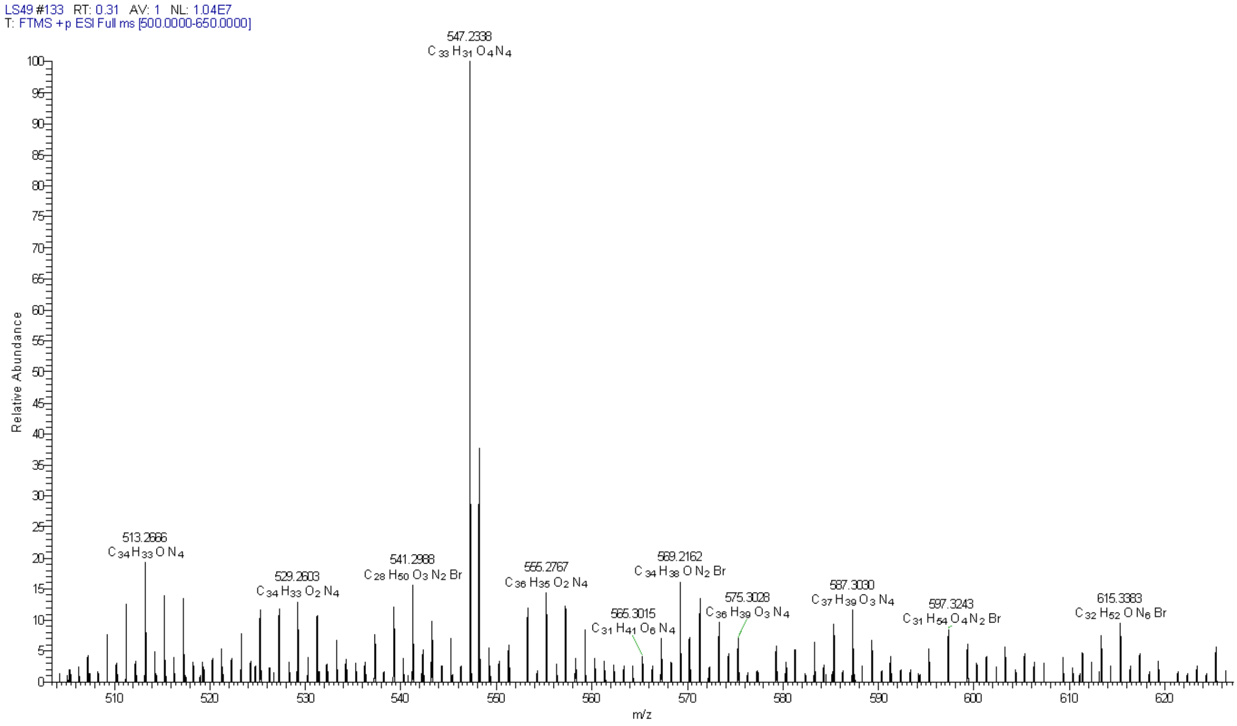
LS49 #133 RT: 0.31 AV: 1 NL: 1.04E7
T: FTMS + p ESI Full ms [500.0000-650.0000]
547.2338
C33 H31 O4 N4
513.2666
C34 H33 O N4
529.2603
C34 H33 O2 N4
541.2988
C28 H50 O3 N2 Br
555.2767
C36 H35 O2 N4
565.3015
C31 H41 O6 N4
569.2162
C34 H38 O N2 Br
575.3028
C36 H39 O3 N4
587.3030
C37 H39 O3 N4
597.3243
C31 H54 O4 N2 Br
615.3383
C32 H52 O N6 Br
Relative Abundance
m/z

(3S,3'R,4'R,4a'S,8a'S)-8a'-hydroxy-1-methyl-3'-nitro-2-oxo-4'-phenyl-3',4',4a',5',6',7',8',8a'-octahydro-1'H-spiro[indoline-3,2'-naphthalene]-1',1'-dicarbonitrile (5f)

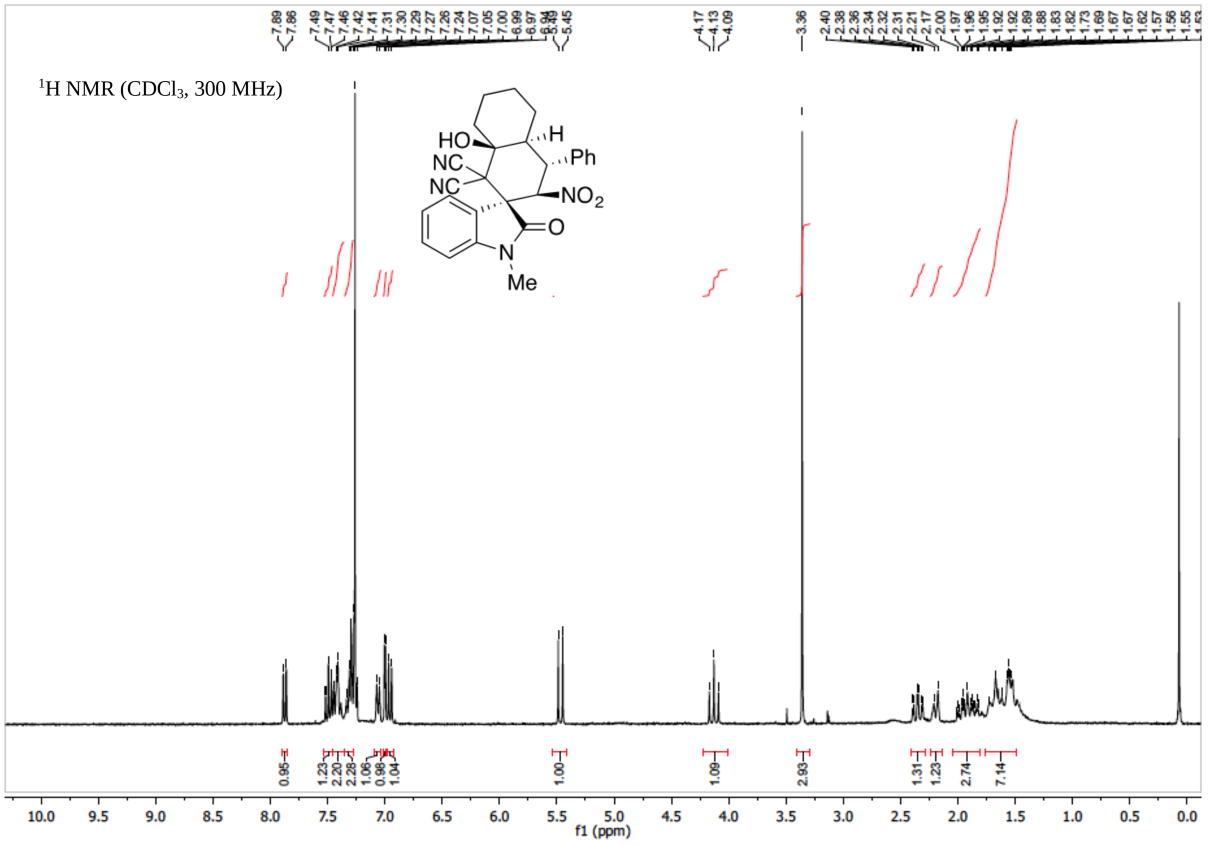

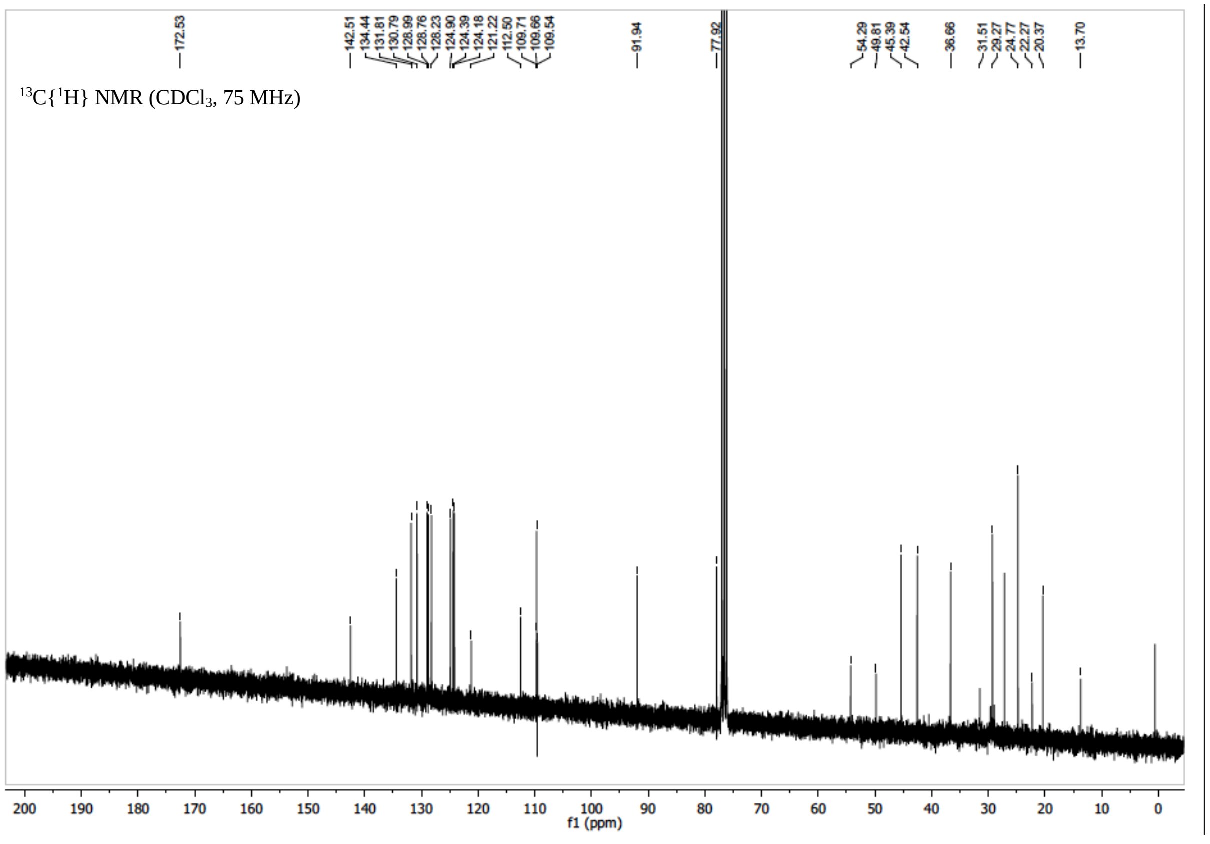

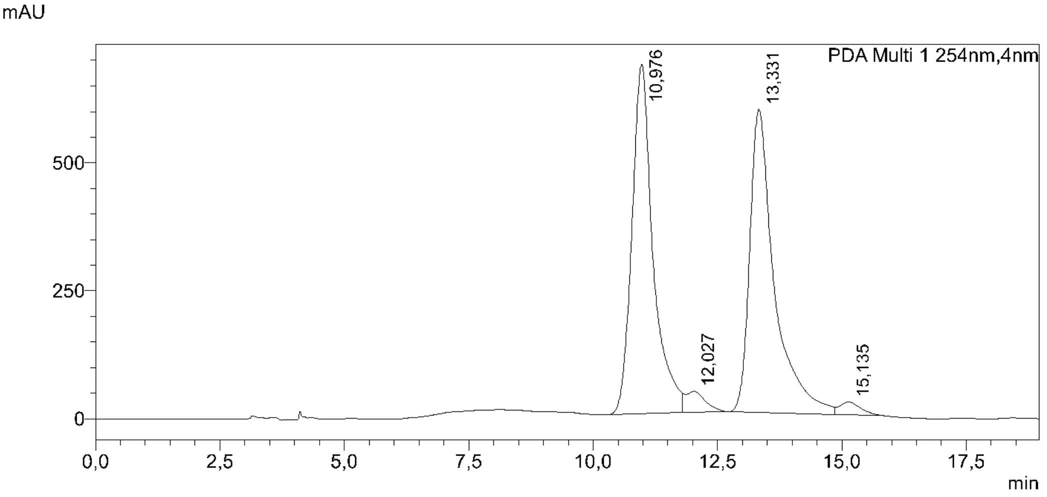

PDA Ch1 254nm

Peak#	Ret. Time	Area	Height	Conc.	Unit	Mark	Name
1	10,976	20193590	682610	47,520		M	
2	12,027	1203268	41767	2,832		V M	
3	13,331	20372904	592141	47,942		M	
4	15,135	725158	24646	1,706		V M	
Total		42494919	1341164				

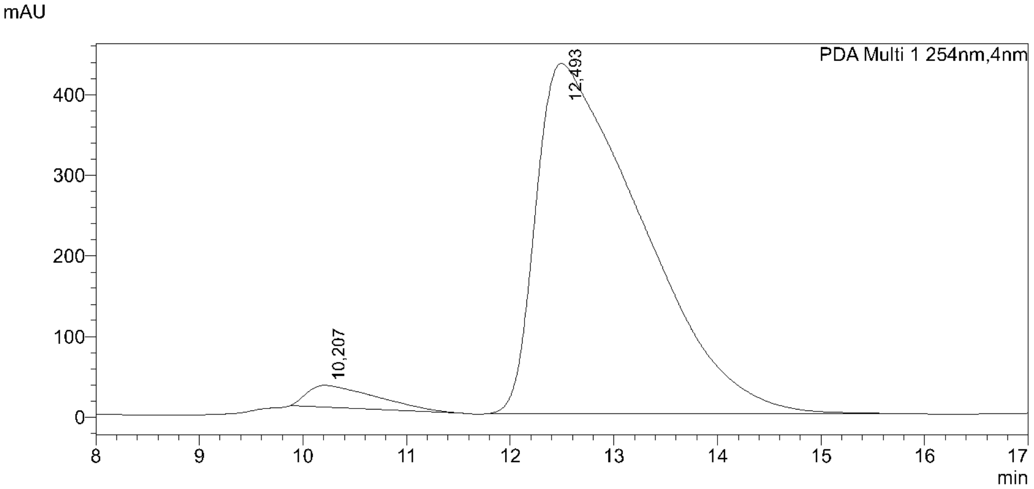

PDA Ch1 254nm

Peak#	Ret. Time	Area	Height	Conc.	Unit	Mark	Name
1	10,207	1259437	26801	3,927		M	
2	12,493	30813386	434631	96,073		M	
Total		32072823	461433				

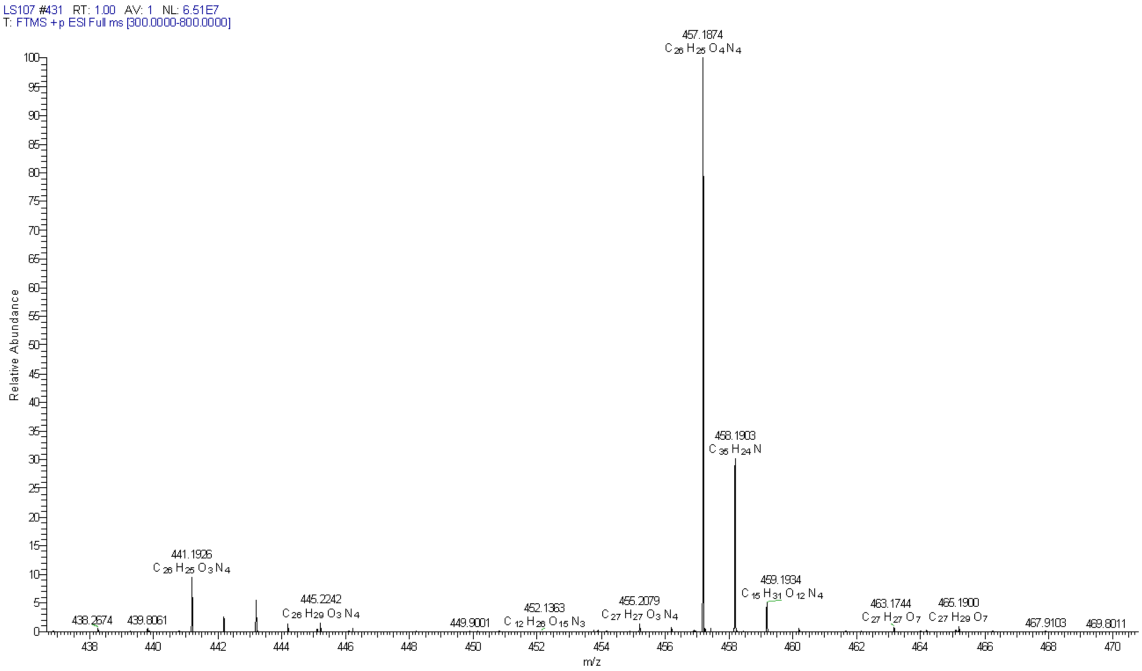
LS107 #431 RT: 1.00 AV: 1 NL: 6.51E7
T: FTMS + p ESI Full ms [300.0000-800.0000]
457.1874
C 26 H 25 O 4 N 4
458.1903
C 35 H 24 N
441.1926
C 26 H 25 O 3 N 4
459.1934
C 15 H 31 O 12 N 4
445.2242
C 26 H 29 O 3 N 4
452.1363
C 12 H 26 O 15 N 3
455.2079
C 27 H 27 O 3 N 4
463.1744
C 27 H 27 O 7
465.1900
C 27 H 29 O 7
438.2674
439.8061
449.9001
467.9103
469.8011
Relative Abundance
m/z

(3S,3'R,4'R,4a'S,8a'S)-5-chloro-8a'-hydroxy-1-methyl-3'-nitro-2-oxo-4'-phenyl-3',4',4a',5',6',7',8',8a'-octahydro-1'H-spiro[indoline-3,2'-naphthalene]-1',1'-dicarbonitrile (5g)

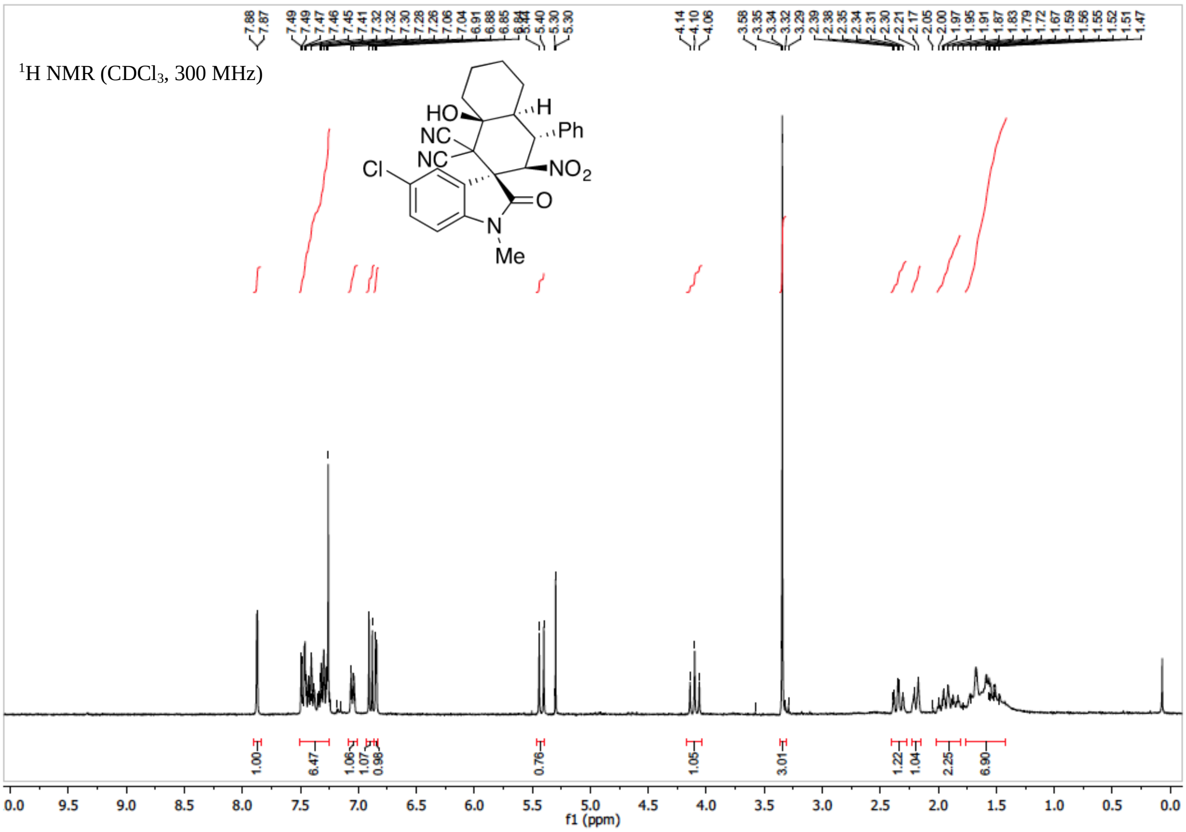

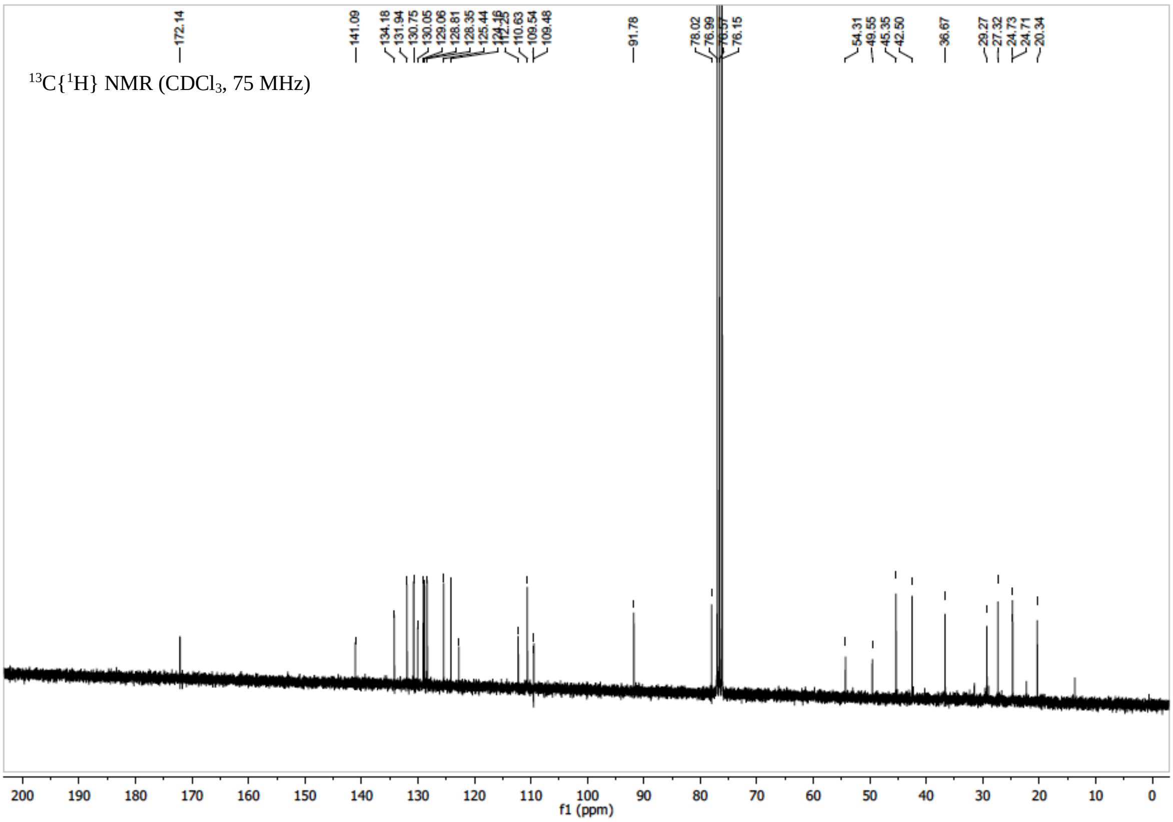

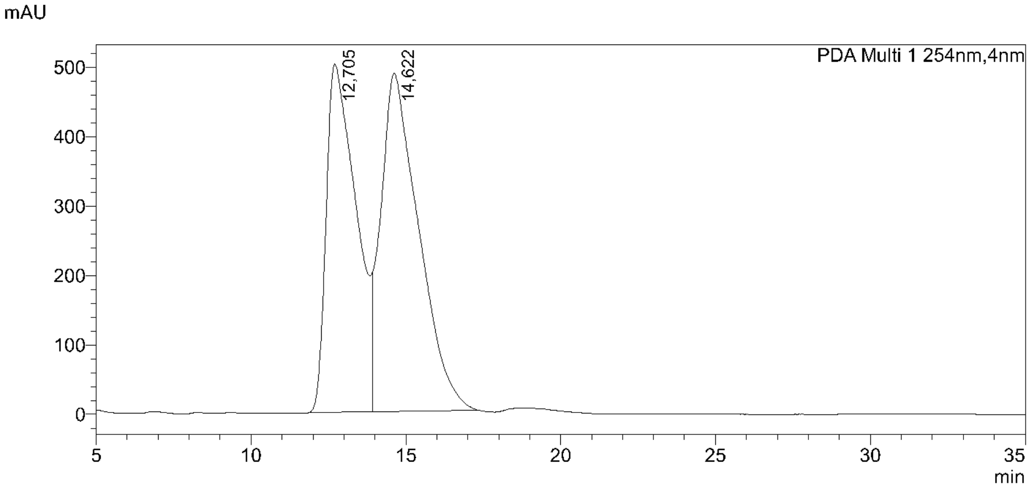

PDA Ch1 254nm

Peak#	Ret. Time	Area	Height	Conc.	Unit	Mark	Name
1	12,705	33985775	501598	45,211		M	
2	14,622	41185706	487092	54,789		V M	
Total		75171481	988690				

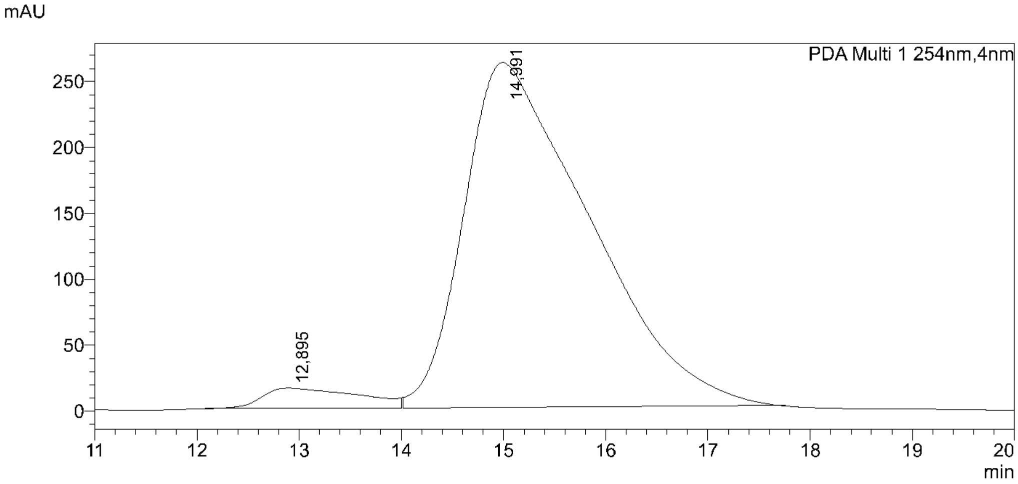

PDA Ch1 254nm

Peak#	Ret. Time	Area	Height	Conc.	Unit	Mark	Name
1	12,895	971148	14945	4,161		M	
2	14,991	22367689	261478	95,839		M	
Total		23338837	276423				

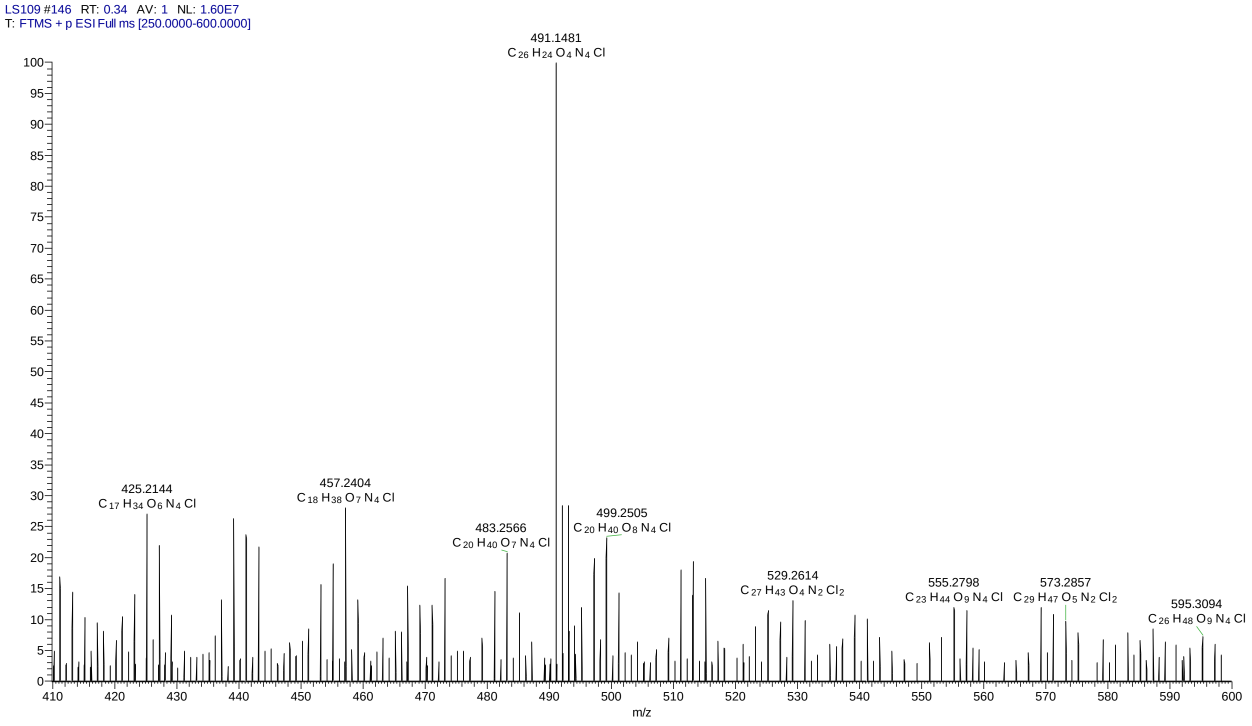

LS109 #146 RT: 0.34 AV: 1 NL: 1.60E7
T: FTMS + p ESI Full ms [250.0000-600.0000]
491.1481
C 26 H 24 O 4 N 4 Cl
425.2144
C 17 H 34 O 6 N 4 Cl
457.2404
C 18 H 38 O 7 N 4 Cl
483.2566
C 20 H 40 O 7 N 4 Cl
499.2505
C 20 H 40 O 8 N 4 Cl
529.2614
C 27 H 43 O 4 N 2 Cl 2
555.2798
C 23 H 44 O 9 N 4 Cl
573.2857
C 29 H 47 O 5 N 2 Cl 2
595.3094
C 26 H 48 O 9 N 4 Cl
m/z

(3S,3'R,4'R,4a'S,8a'S)-8a'-hydroxy-1,5-dimethyl-3'-nitro-2-oxo-4'-phenyl-3',4',4a',5',6',7',8',8a'-octahydro-1'H-spiro[indoline-3,2'-naphthalene]-1',1'-dicarbonitrile (5h)

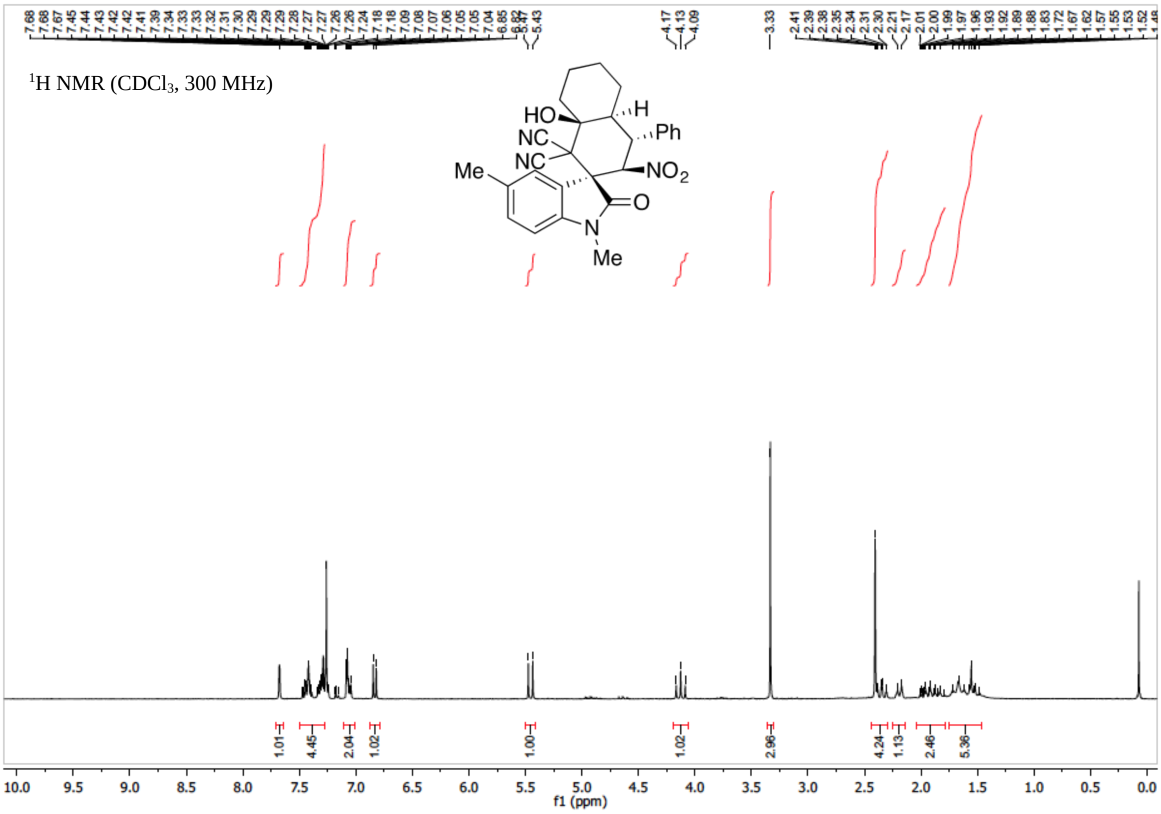

.

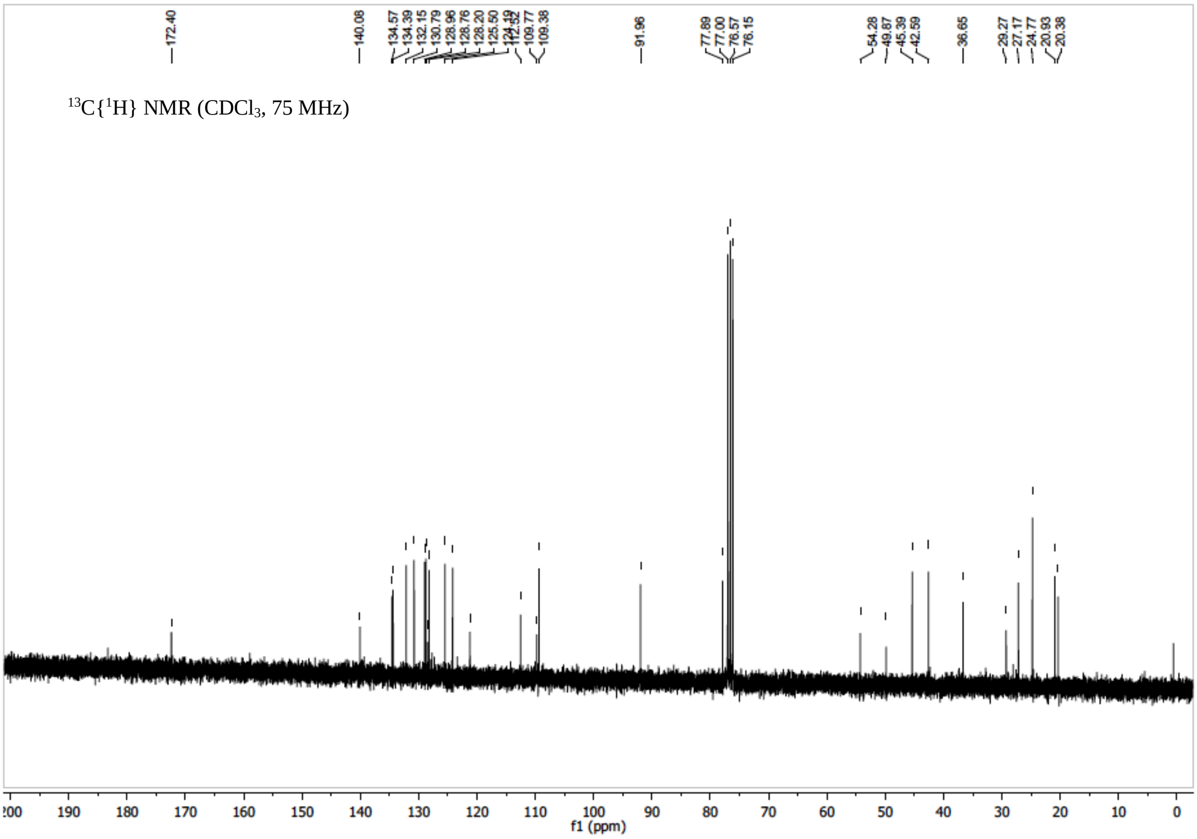

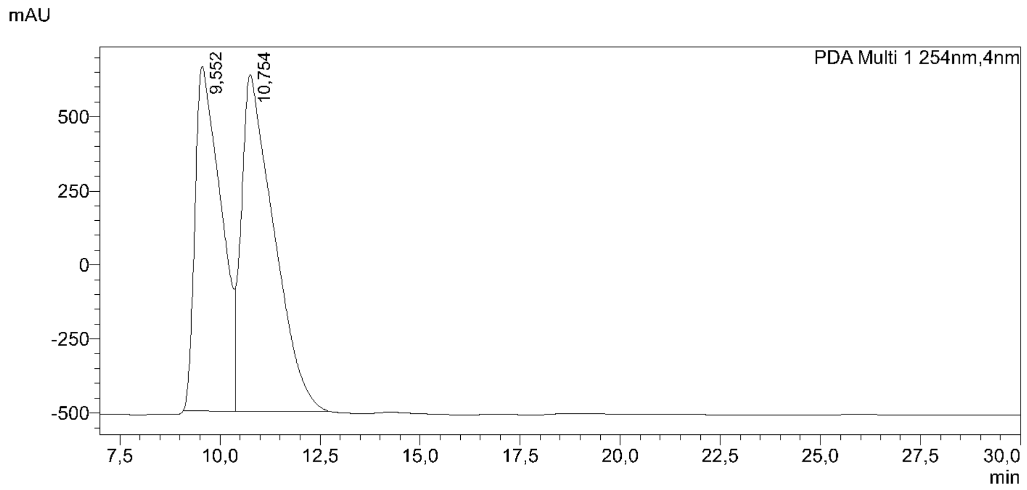

PDA Ch1 254nm

Peak#	Ret. Time	Area	Height	Conc.	Unit	Mark	Name
1	9,552	52886476	1164120	45,199		M	
2	10,754	64121295	1136693	54,801		V M	
Total		117007771	2300813				

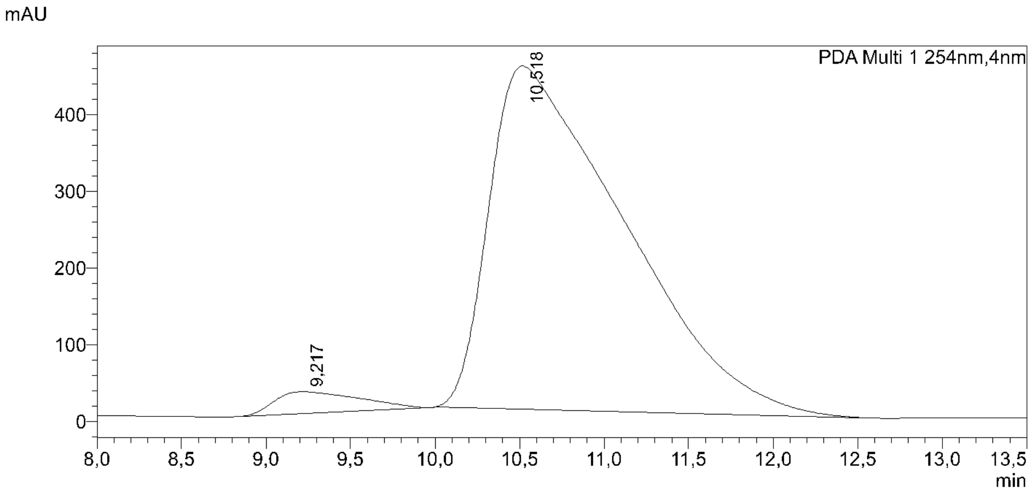

PDA Ch1 254nm

Peak#	Ret. Time	Area	Height	Conc.	Unit	Mark	Name
1	9,217	973022	28347	3,758		M	
2	10,518	24918183	447555	96,242		M	
Total		25891206	475902				

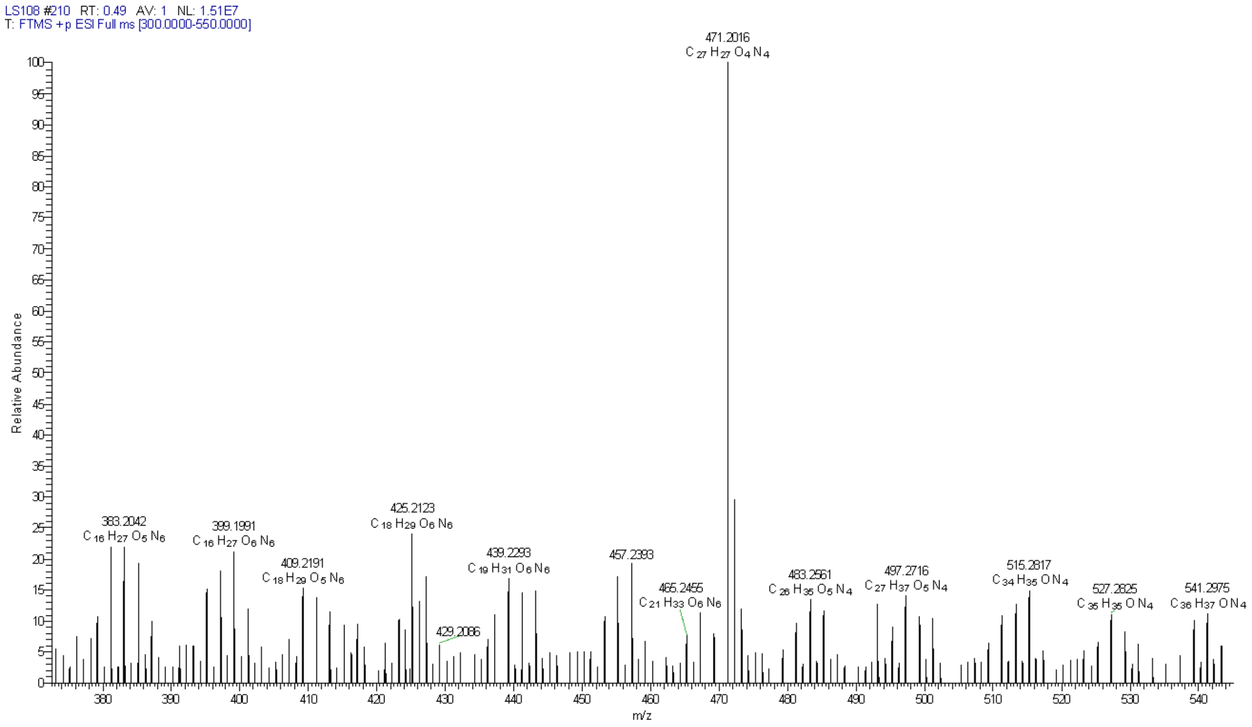
LS108 #210 RT: 0.49 AV: 1 NL: 1.51E7
T: FTMS + p ESI Full ms [300.0000-550.0000]
471.2016
C 27 H 27 O 4 N 4
383.2042
C 16 H 27 O 5 N 6
399.1991
C 16 H 27 O 6 N 6
409.2191
C 18 H 29 O 5 N 6
425.2123
C 18 H 29 O 6 N 6
429.2086
439.2293
C 19 H 31 O 6 N 6
457.2393
465.2455
C 21 H 33 O 6 N 6
483.2561
C 26 H 35 O 5 N 4
497.2716
C 27 H 37 O 5 N 4
515.2817
C 34 H 35 O N 4
527.2825
C 35 H 35 O N 4
541.2975
C 36 H 37 O N 4
Relative Abundance
m/z

(3S,3'R,4'R,4a'S,8a'S)-1-ethyl-8a'-hydroxy-3'-nitro-2-oxo-4'-phenyl-3',4',4a',5',6',7',8',8a'-octahydro-1'H-spiro[indoline-3,2'-naphthalene]-1',1'-dicarbonitrile (5i)

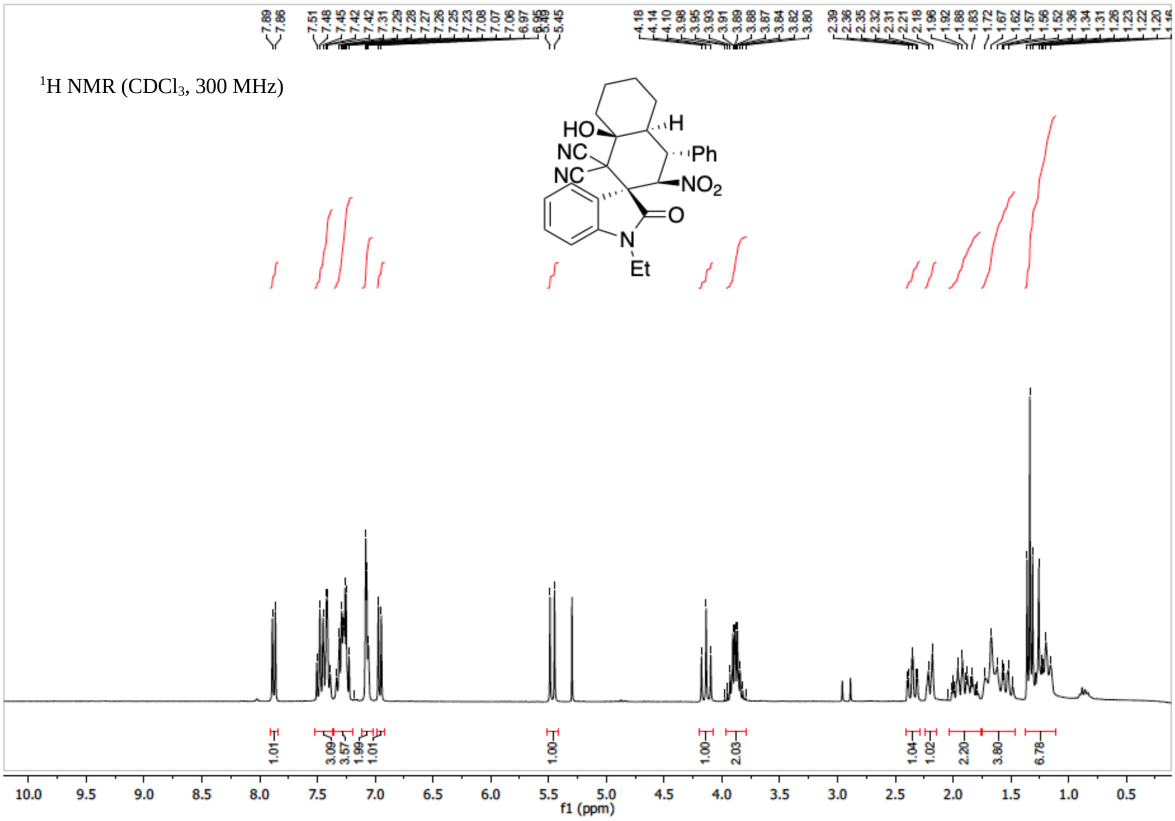

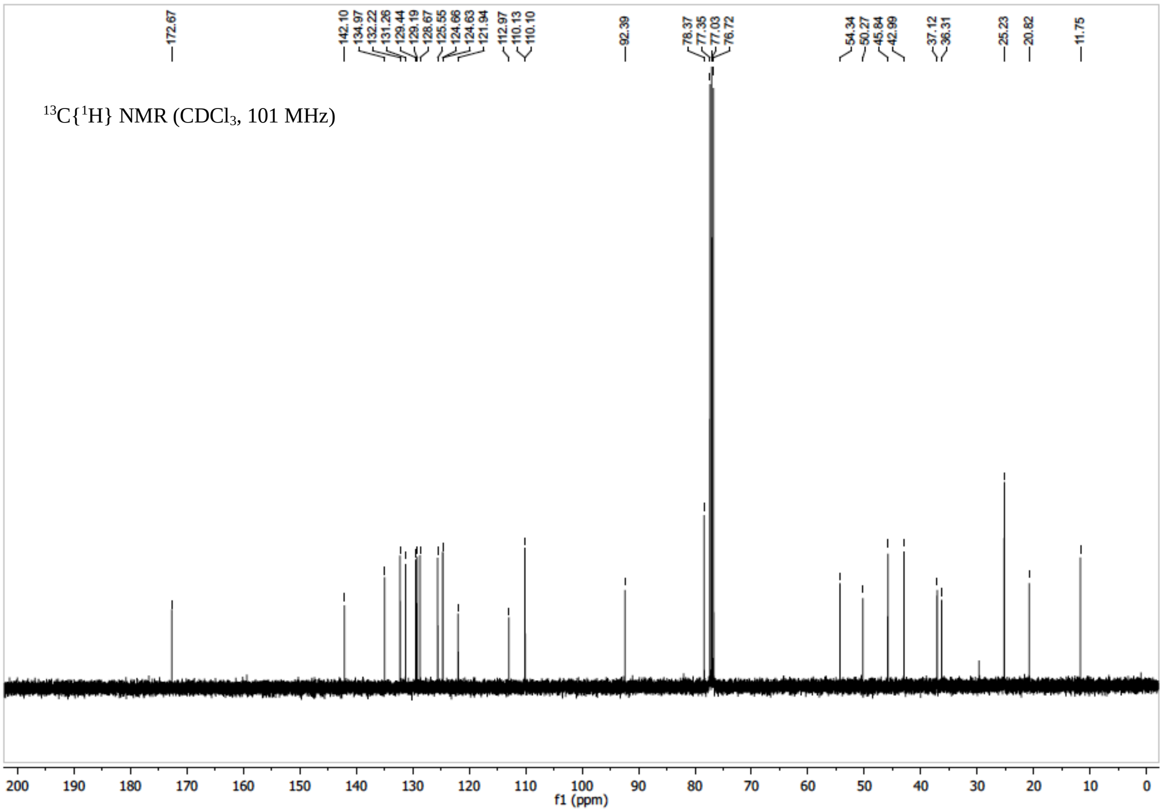

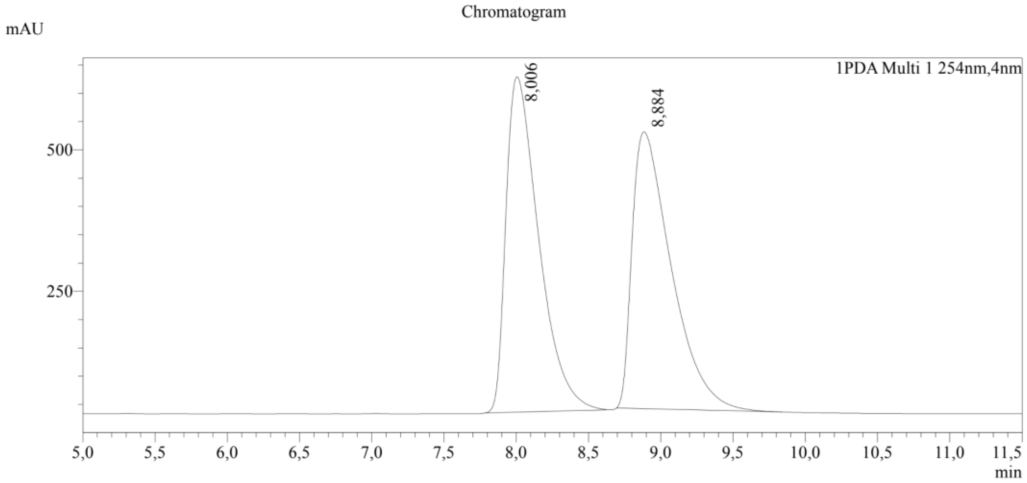

<Peak Table>

PDA Ch1 254nm

Peak#	Ret. Time	Area	Height	Conc.	Unit	Mark	Name
1	8,006	10422698	658312	50,378		M	
2	8,884	10266235	543883	49,622		M	
Total		20688933	1202195				

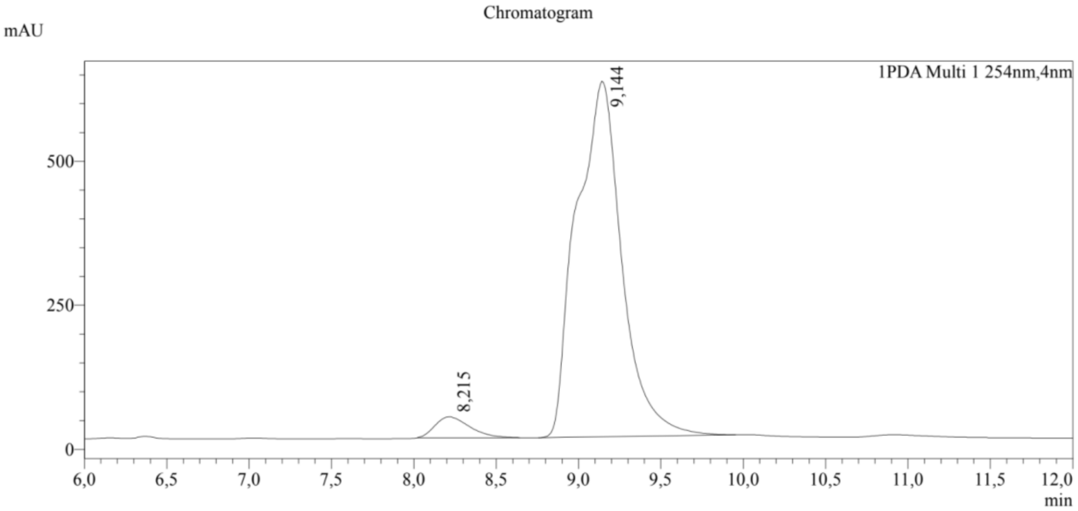

<Peak Table>

PDA Ch1 254nm

Peak#	Ret. Time	Area	Height	Conc.	Unit	Mark	Name
1	8,215	582040	40088	4,238		M	
2	9,144	13151724	685933	95,762		M	
Total		13733764	726022				

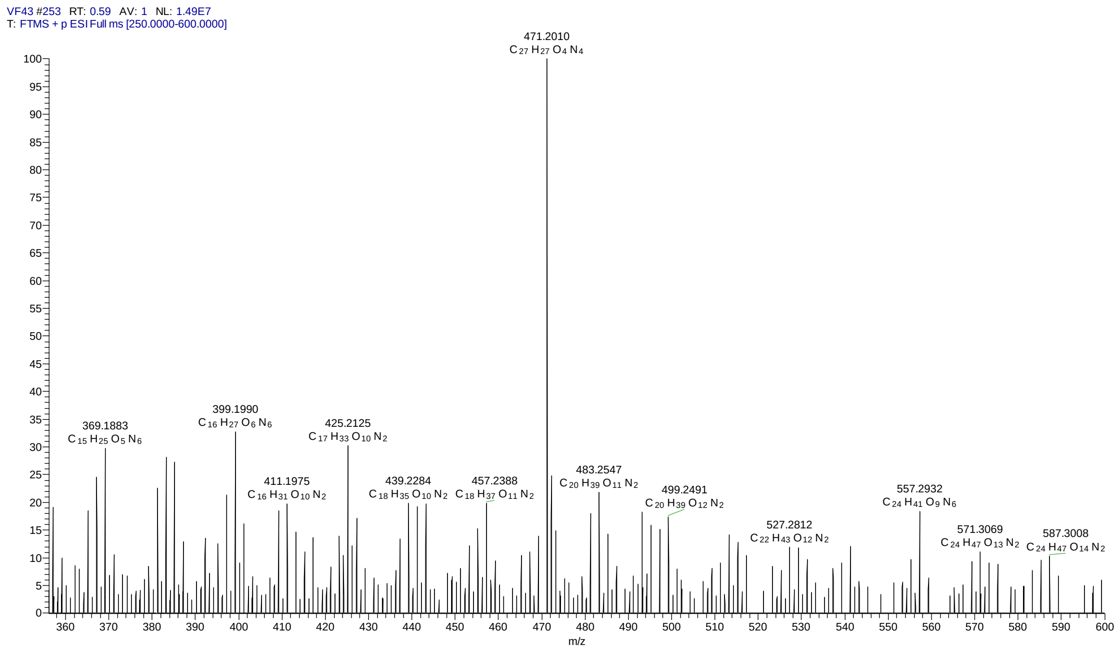

VF43 #253 RT: 0.59 AV: 1 NL: 1.49E7
T: FTMS + p ESI Full ms [250.0000-600.0000]
471.2010
C 27 H 27 O 4 N 4
369.1883
C 15 H 25 O 5 N 6
399.1990
C 16 H 27 O 6 N 6
411.1975
C 16 H 31 O 10 N 2
425.2125
C 17 H 33 O 10 N 2
439.2284
C 18 H 35 O 10 N 2
457.2388
C 18 H 37 O 11 N 2
483.2547
C 20 H 39 O 11 N 2
499.2491
C 20 H 39 O 12 N 2
527.2812
C 22 H 43 O 12 N 2
557.2932
C 24 H 41 O 9 N 6
571.3069
C 24 H 47 O 13 N 2
587.3008
C 24 H 47 O 14 N 2
m/z

(3S,3'R,4'R,4a'S,8a'S)-5-chloro-1-ethyl-8a'-hydroxy-3'-nitro-2-oxo-4'-phenyl-3',4',4a',5',6',7',8',8a'-octahydro-1'H-spiro[indoline-3,2'-naphthalene]-1',1'-dicarbonitrile (5j)

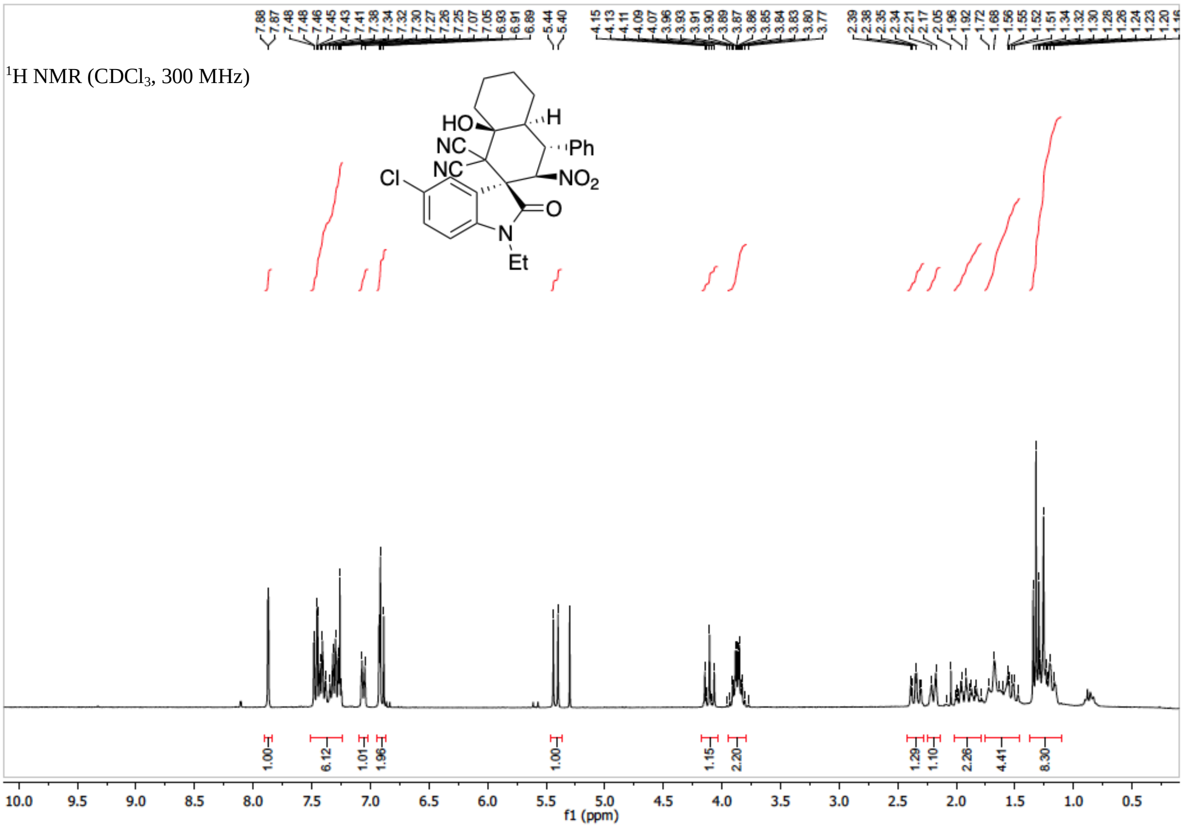

.

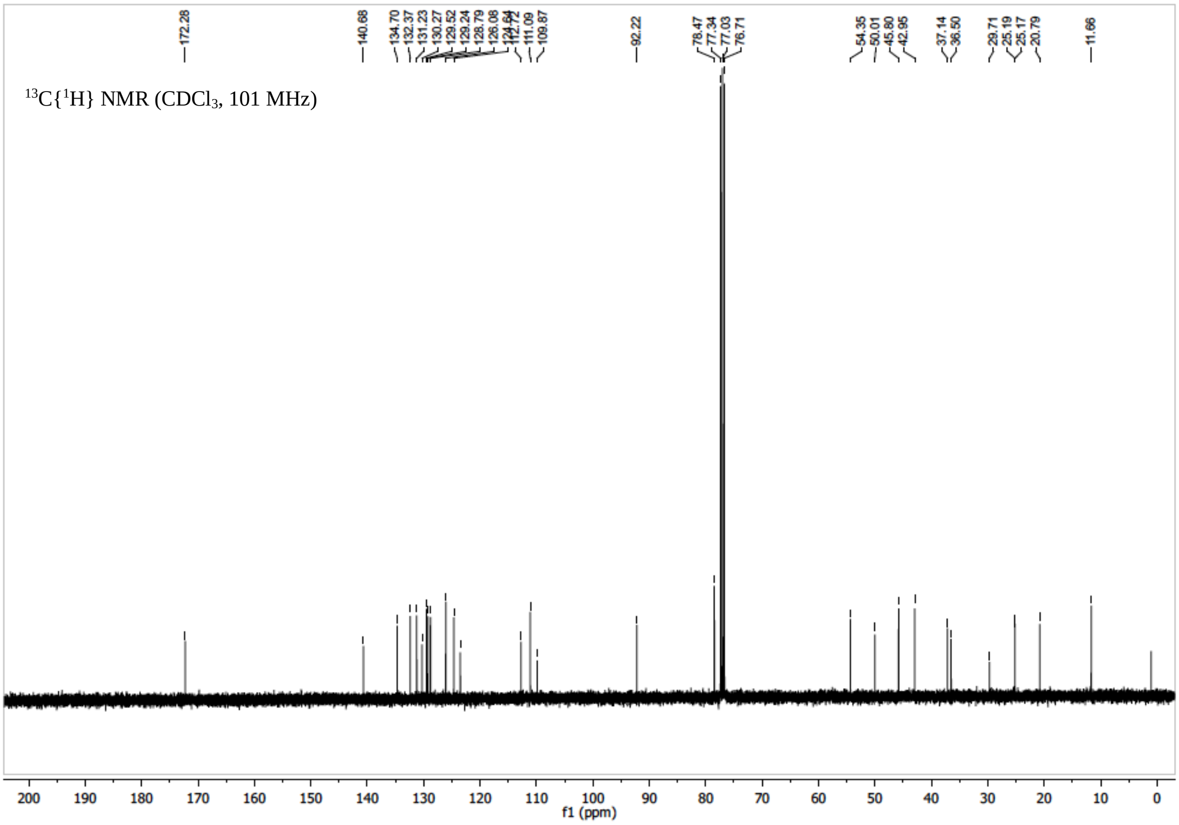

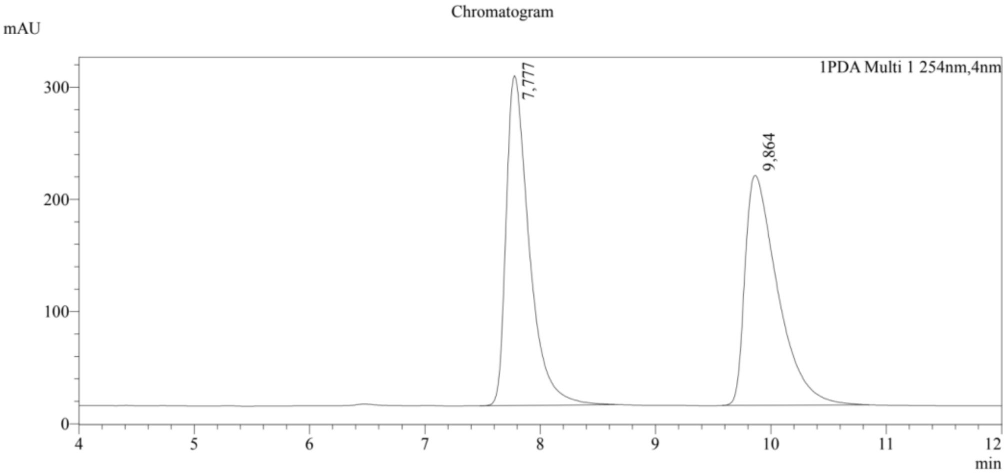

<Peak Table>

PDA Ch1 254nm

Peak#	Ret. Time	Area	Height	Conc.	Unit	Mark	Name
1	7,777	4539867	326794	50,291		M	
2	9,864	4487340	227804	49,709		M	
Total		9027207	554598				

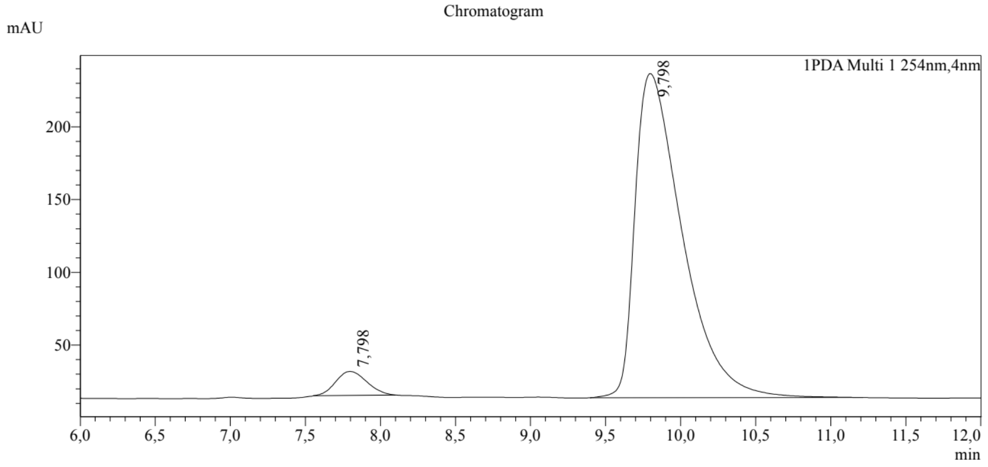

<Peak Table>

PDA Ch1 254nm

Peak#	Ret. Time	Area	Height	Conc.	Unit	Mark	Name
1	7,798	265651	18356	4,662		M	
2	9,798	5432799	247416	95,338		M	
Total		5698450	265772				

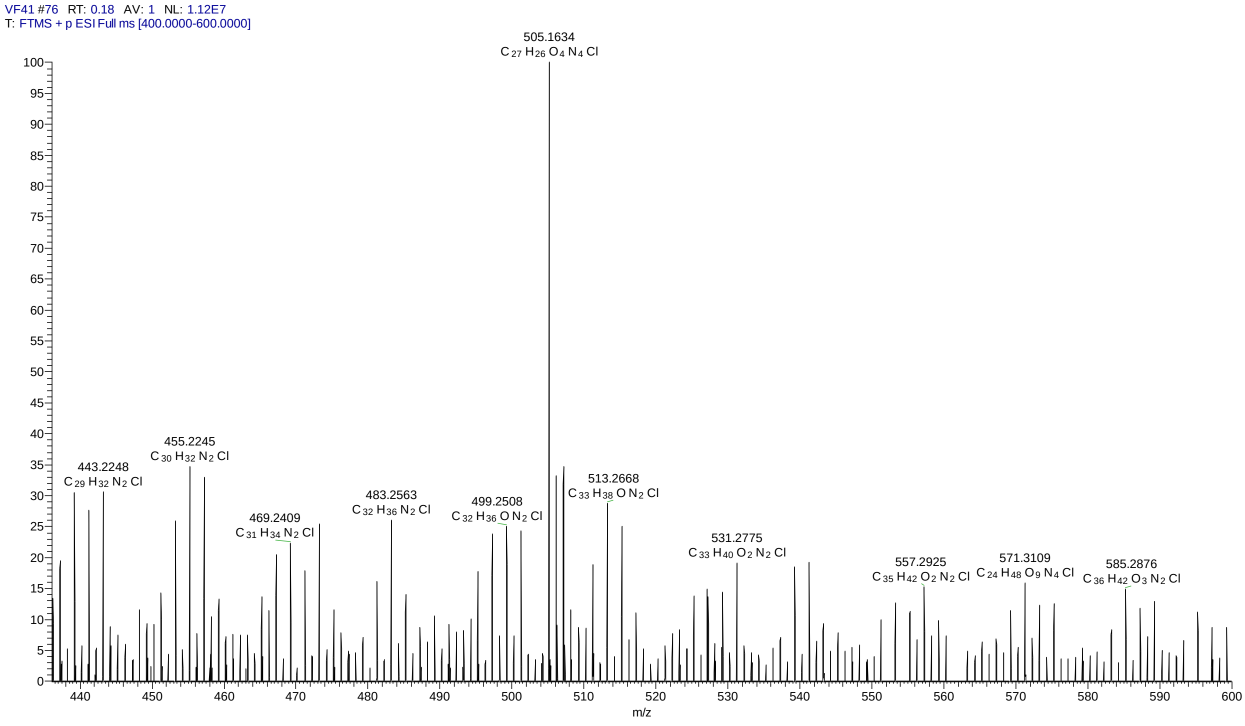

VF41 #76 RT: 0.18 AV: 1 NL: 1.12E7
T: FTMS + p ESI Full ms [400.0000-600.0000]
505.1634
C 27 H 26 O 4 N 4 Cl
455.2245
C 30 H 32 N 2 Cl
443.2248
C 29 H 32 N 2 Cl
469.2409
C 31 H 34 N 2 Cl
483.2563
C 32 H 36 N 2 Cl
499.2508
C 32 H 36 O N 2 Cl
513.2668
C 33 H 38 O N 2 Cl
531.2775
C 33 H 40 O 2 N 2 Cl
557.2925
C 35 H 42 O 2 N 2 Cl
571.3109
C 24 H 48 O 9 N 4 Cl
585.2876
C 36 H 42 O 3 N 2 Cl
m/z

(3S,3'R,4'R,4a'S,8a'S)-1-ethyl-8a'-hydroxy-5-methyl-3'-nitro-2-oxo-4'-phenyl-3',4',4a',5',6',7',8',8a'-octahydro-1'H-spiro[indoline-3,2'-naphthalene]-1',1'-dicarbonitrile (5k)

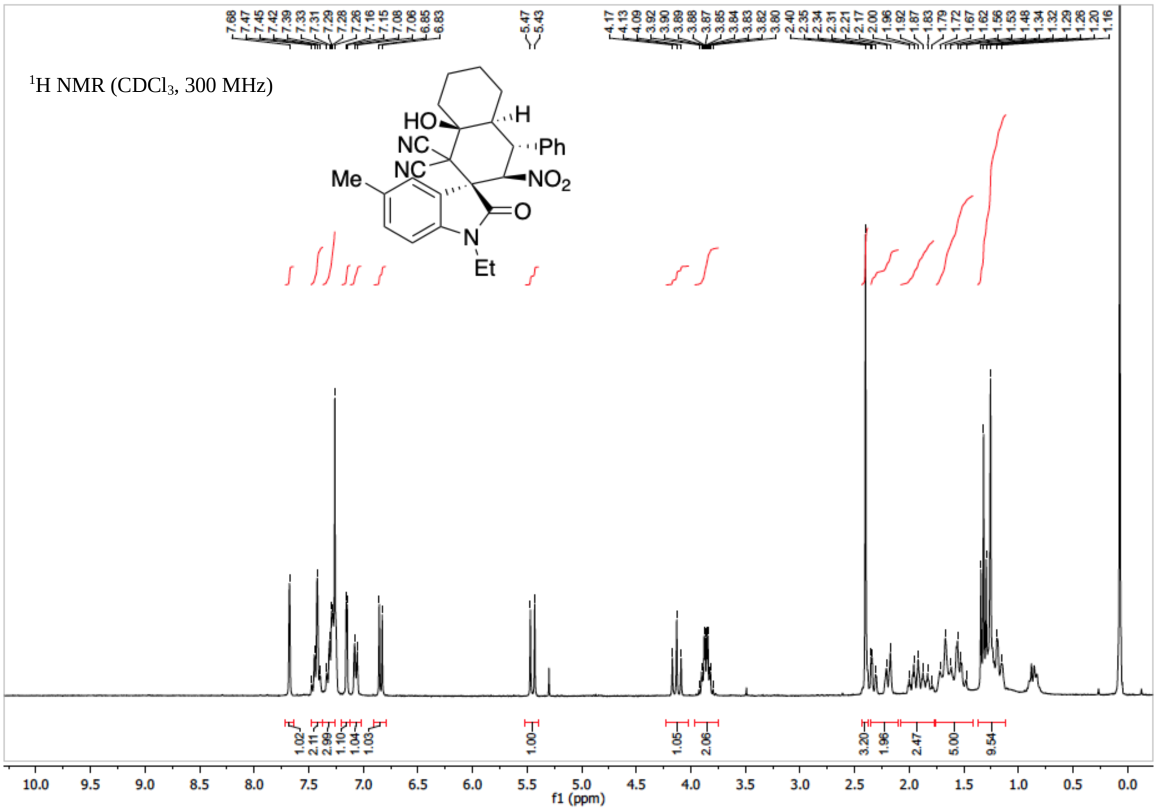

.

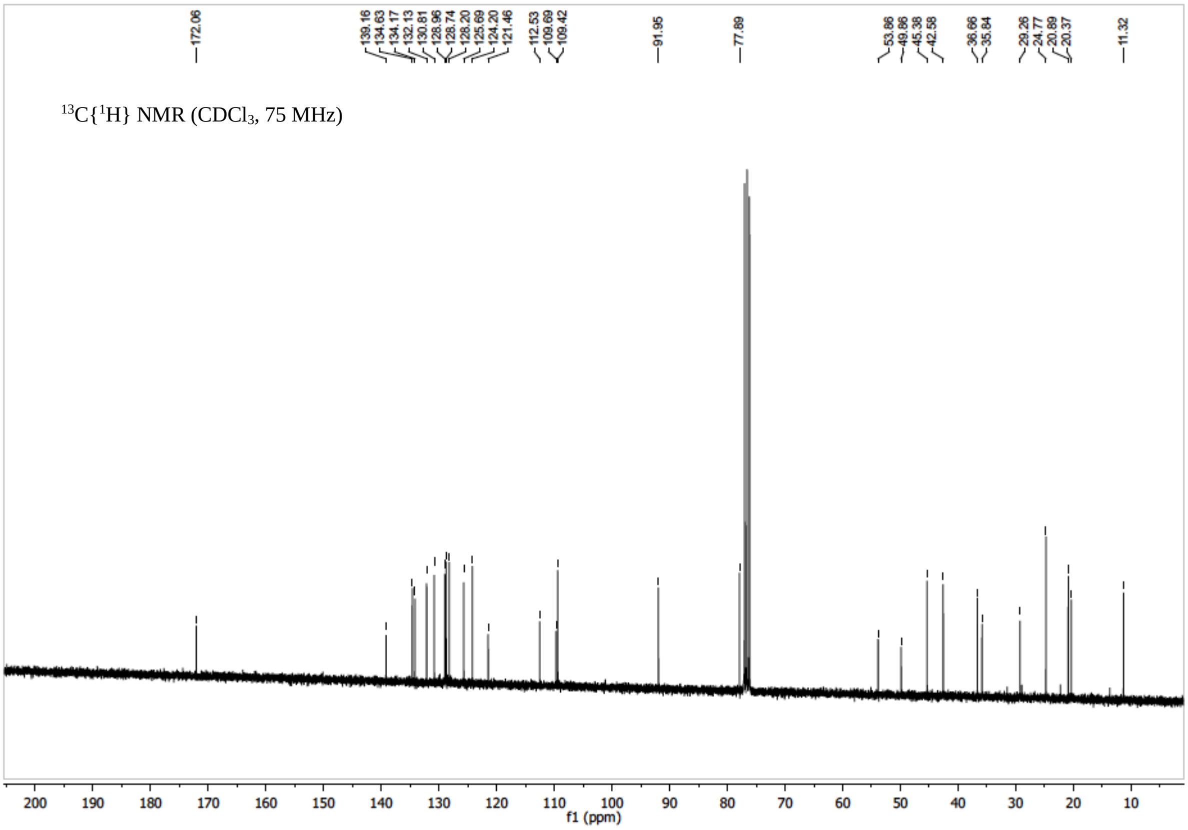

Chromatogram

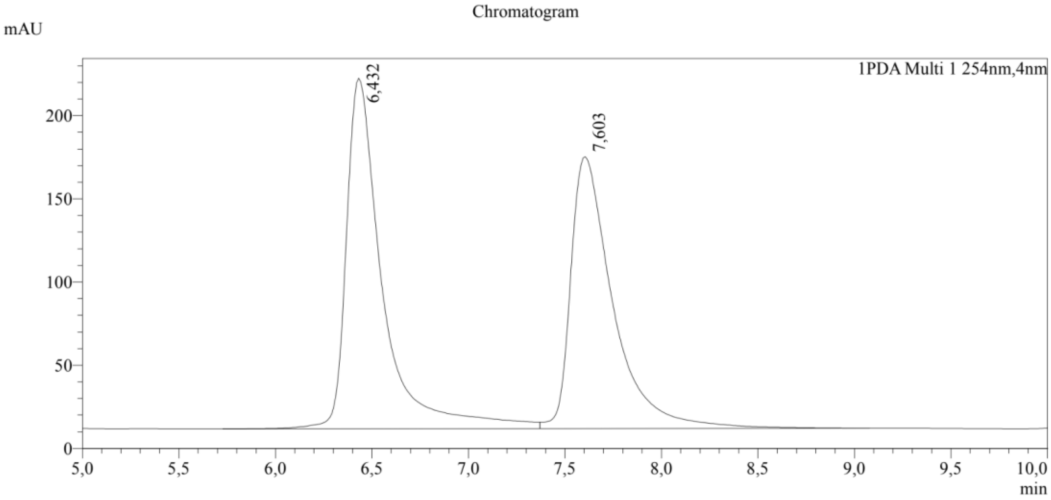

<Peak Table>

PDA Ch1 254nm

Peak#	Ret. Time	Area	Height	Conc.	Unit	Mark	Name
1	6,432	3063889	234266	52,467			
2	7,603	2775764	181635	47,533		V	
Total		5839653	415901				

<Chromatogram>

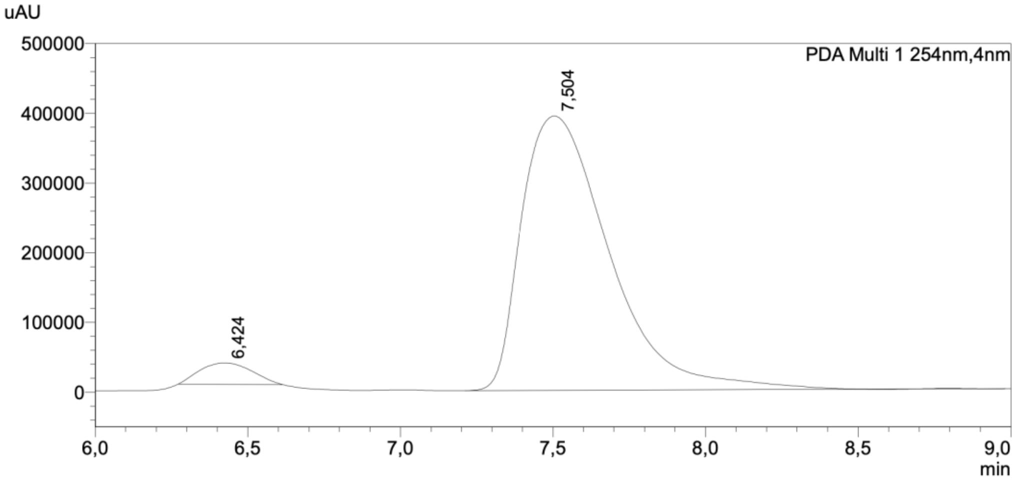

<Peak Table>

PDA Ch1 254nm

Peak#	Ret. Time	Area	Height	Area%
1	6,424	365596	30537	4,426
2	7,504	7894499	393399	95,574
Total		8260095	423937	100,000

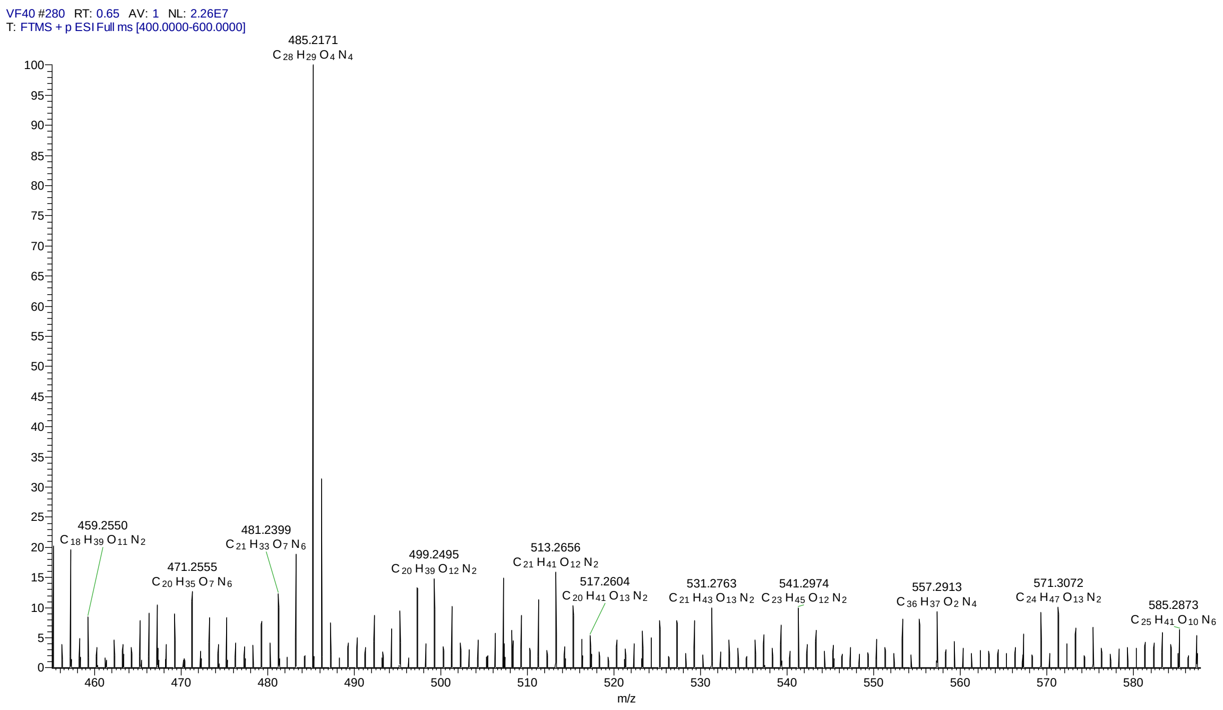

VF40 #280 RT: 0.65 AV: 1 NL: 2.26E7
T: FTMS + p ESI Full ms [400.0000-600.0000]
485.2171
C 28 H 29 O 4 N 4
459.2550
C 18 H 39 O 11 N 2
471.2555
C 20 H 35 O 7 N 6
481.2399
C 21 H 33 O 7 N 6
499.2495
C 20 H 39 O 12 N 2
513.2656
C 21 H 41 O 12 N 2
517.2604
C 20 H 41 O 13 N 2
531.2763
C 21 H 43 O 13 N 2
541.2974
C 23 H 45 O 12 N 2
557.2913
C 36 H 37 O 2 N 4
571.3072
C 24 H 47 O 13 N 2
585.2873
C 25 H 41 O 10 N 6
m/z
